# Feature-Based Molecular Networking—An Exciting Tool to Spot Species of the Genus *Cortinarius* with Hidden Photosensitizers

**DOI:** 10.3390/metabo11110791

**Published:** 2021-11-19

**Authors:** Fabian Hammerle, Luis Quirós-Guerrero, Adriano Rutz, Jean-Luc Wolfender, Harald Schöbel, Ursula Peintner, Bianka Siewert

**Affiliations:** 1 Institute of Pharmacy, Pharmacognosy, Center for Molecular Biosciences (CMBI), University of Innsbruck, CCB—Innrain 80/82, 6020 Innsbruck, Austria; fabian.hammerle@uibk.ac.at; 2 Phytochemistry and Bioactive Natural Products, School of Pharmaceutical Sciences, University of Geneva, CMU—Rue Michel-Servet 1, 1211 Geneva, Switzerland; luis.guerrero@unige.ch (L.Q.-G.); adriano.rutz@unige.ch (A.R.); jean-luc.wolfender@unige.ch (J.-L.W.); 3 Institute of Pharmaceutical Sciences of Western Switzerland, University of Geneva, CMU—Rue Michel-Servet 1, 1211 Geneva, Switzerland; 4 Department of Biotechnology, MCI—The Entrepreneurial School, Maximilianstraße 2, 6020 Innsbruck, Austria; harald.schoebel@mci.edu; 5 Institute of Microbiology, University of Innsbruck, Technikerstrasse 25, 6020 Innsbruck, Austria; ursula.peintner@uibk.ac.at

**Keywords:** FBMN, fungal photosensitizers, fungal pigments, photodynamic therapy

## Abstract

Fungi have developed a wide array of defense strategies to overcome mechanical injuries and pathogen infections. Recently, photoactivity has been discovered by showing that pigments isolated from *Cortinarius uliginosus* produce singlet oxygen under irradiation. To test if this phenomenon is limited to dermocyboid Cortinarii, six colourful *Cortinarius* species belonging to different classical subgenera (i.e., *Dermocybe*, *Leprocybe*, *Myxacium*, *Phlegmacium*, and *Telamonia*) were investigated. Fungal extracts were explored by the combination of in vitro photobiological methods, UHPLC coupled to high-resolution tandem mass spectrometry (UHPLC-HRMS^2^), feature-based molecular networking (FBMN), and metabolite dereplication techniques. The fungi *C.* *rubrophyllus* (*Dermocybe*) and *C.* *xanthophyllus* (*Phlegmacium*) exhibited promising photobiological activity in a low concentration range (1–7 µg/mL). Using UHPLC-HRMS^2^-based metabolomic tools, the underlying photoactive principle was investigated. Several monomeric and dimeric anthraquinones were annotated as compounds responsible for the photoactivity. Furthermore, the results showed that light-induced activity is not restricted to a single subgenus, but rather is a trait of *Cortinarius* species of different phylogenetic lineages and is linked to the presence of fungal anthraquinones. This study highlights the genus *Cortinarius* as a promising source for novel photopharmaceuticals. Additionally, we showed that putative dereplication of natural photosensitizers can be done by FBMN.

## 1. Introduction

Unlike animals, fungi lack a central nervous system, responding to life-threatening events by releasing catecholamines and preparing the organism for fight or flight [1,2]. However, to circumvent the disadvantage of their sessile lifestyle, they have developed ways of defending themselves from predators and parasites. Along with anatomical and mechanical characteristics, a vast array of chemical defense mechanisms evolved [3]. These mechanisms comprise diverse bioactive compounds, some of them being permanently present in the respective organism, others being only synthesized when needed [4]. An example of the so-called constitutive chemical defense is the steady production of highly toxic octapeptides (e.g., α-, γ-, and ε-amanitin) by the death cap (*Amanita phalloides*) [5]. The conversion of linoleic acid into the antifungal molecule (3*R*)-1-octen-3-ol [6] only occurs when the fruiting bodies of the button mushroom (*Agaricus bisporus*) are wounded and is thus a classic example of the wound-activated chemical defense.

Recently, hints of an overlooked chemical defense mechanism in the kingdom Fungi were discovered: photoactive metabolites in dermocyboid Cortinarii [7,8]. Fungi from this phylogenetic lineage are characterized by brightly colored fruiting bodies, which owe their hues to polyketide pigments [9]. Some of these fungal anthraquinones, e.g., 7,7′-biphyscion, exhibited a promising singlet oxygen production yield and were highly phototoxic, as they induced light-dependent apoptosis in the nanomolar range [8]. Therefore, a thorough investigation of the photoactivity phenomenon occurring in the genus *Cortinarius* combined with the identification and isolation of photoactive compounds could contribute to a more comprehensive understanding of fungal defense strategies (i.e., ecological role). Simultaneously, it could benefit the field of photodynamic therapy (PDT). PDT is a treatment modality for cancer and is based on the interaction of special drugs (photosensitizers, PSs) with light. After irradiation with a suitable light source, the PS reaches an excited state, transfers its excess energy to environmental triplet oxygen, and generates singlet oxygen (^1^O_2_/Type II reaction), inducing cell death [10,11]. PDT in its current state heavily depends on porphyrin-like structures and transition metal complexes [12,13], whereas the search for new photoactive scaffolds from nature is neglected [14].

The genus *Cortinarius* is one of the largest genera in the division of Basidiomycota. Fungi belonging to this genus produce gilled fruiting bodies with a typical cortina. They have rusty brown spores and are mycorrhizal. Due to their intensely colored fruiting bodies, they sparked the interest of chemists early on: in 1925, the isolation of the pigments dermocybin and emodin from *C. sanguineus* was already achieved [15]. Over time, countless other pigments have been isolated and identified from Cortinarii [16,17], mainly by collecting large quantities of fresh fruiting bodies of one species (kg range) followed by extraction and labor-intensive separation steps. However, some *Cortinarius* species are rarely found in nature (e.g., *C. xanthophyllus*) [18]. Therefore, it is difficult to obtain sufficient amounts of reliably identified biomaterial for mycochemical analyses. Thus, rapid progress in the field of mycochemistry is to some extent impeded.

Since the early 20th century, chemists have relied on standard procedures, such as thin-layer chromatography (TLC), to elucidate the pigment profiles of Cortinarii [9]. To continue their work, however, a new analytical approach with high sensitivity, reliability, and easy accessibility is needed that meets today’s data-driven standards [19]. A promising technique is feature-based molecular networking (FBMN), a metabolomics tool based on ultra-high performance liquid chromatography coupled to high-resolution tandem mass spectrometry (UHPLC-HRMS^2^) [20]. This method allows for visualization of the complex chemical space of metabolites present in extracts and for guessing of the underlying principle of any observed bioactivity to be started simultaneously, with just a few micrograms of material [21]. The first step of this analytical strategy involves the UHPLC-DAD-MS^2^ profiling of a set of extracts, followed by processing of the non-targeted mass spectrometry data e.g., with the open-source software MZmine [22] and the generation of a feature-based molecular network (FBMN) [20] using the Global Natural Products Social Molecular Networking (GNPS) platform [23]. The detected compounds are identified by mass spectral matching against experimental—but limited—data (e.g., GNPS database) and/or utilizing in silico annotation tools such as Sirius [24] or in silico generated libraries (e.g., ISDB [25]). Prioritization of active entities can be achieved by adding additional layers of information by merging taxonomical and chemical/biological data with the FBMN [21,26]. Thus, natural product families that exhibit desired properties (e.g., photoactivity/-cytotoxicity) are highlighted in the network.

The present study investigated the explanatory potential of FBMN on the photochemical and photobiological properties of a unique collection of *Cortinarius* fruiting bodies. In detail, six brightly colored *Cortinarius* species representing classical subgenera (i.e., *Cortinarius rubrophyllus* (*Dermocybe*), *C. venetus* (*Leprocybe*), *C. callisteus* (*Leprocybe*), *C. trivialis* (*Myxacium*), *C. xanthophyllus* (*Phlegmacium*), and *C. traganus* (*Telamonia*)) were studied. As demonstrated in the phylogenetic tree of Figure S1, species of the large subgenera of the genus *Cortinarius* were chosen. This selection was done to test whether photoactivity is restricted to one subgenus (i.e., dermocyboid Cortinarii) or rather is a common trait of the genus *Cortinarius*.

## 2. Results and Discussion

### 2.1. Study Overview

**Figure 1:**
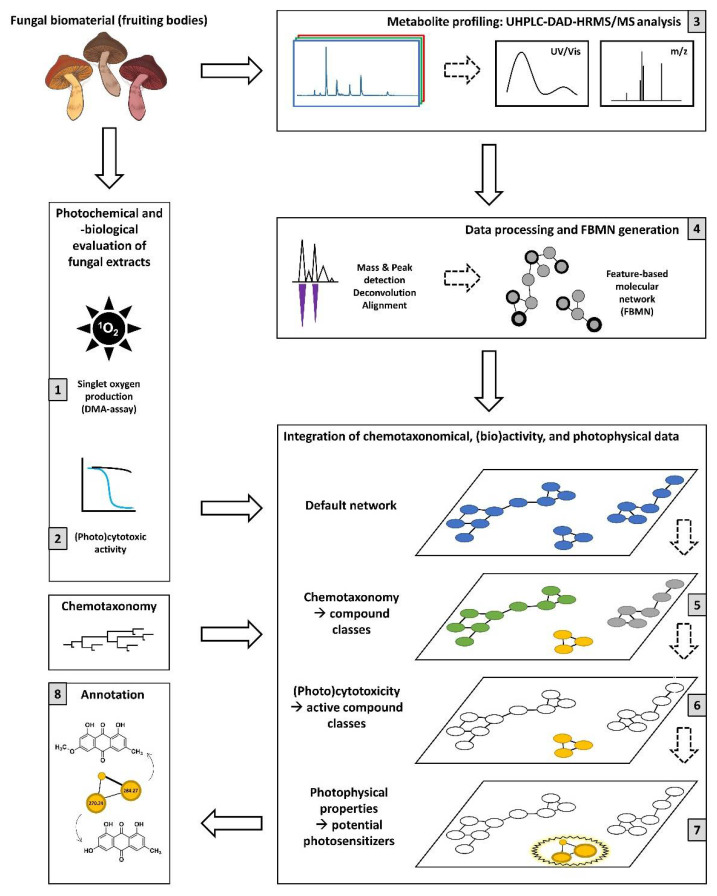
Graphical representation of the analytical strategy used in this study to explore the photochemical and biological properties of different *Cortinarius* species with feature-based molecular networking (FBMN).

To obtain an overview of the photobiological potential of the genus *Cortinarius*, fruiting bodies of the selected species were extracted with acetone. Subsequently, the extracts were submitted to a multifaceted workflow (Figure 1), allowing the recognition of the photobiological active features and the identification of new natural photosensitizers as well as the dereplication of known ones.

In detail, the extracts were submitted to photochemical (Figure 1(1)) and photobiological studies (Figure 1(2)), as well as to a comprehensive set of metabolomic analyses (Figure 1(3)–(7)). While the photochemical ranking was based on the rapid, cell-free 9,10-dimethylanthracene (DMA) assay (Figure 1(1)), the complementary photobiological assay (Figure 1(2)) validated the obtained hits in a cellular setting based on the sulforhodamine B (SRB) assay. The insights—discussed in the first part of this paper—were integrated into the metabolomic study. The latter was based on a state-of-the-art FBMN. After data recording (Figure 1(3)), processing, and generation of the FBMN (Figure 1(4)), chemotaxonomic information (i.e., compound class information based on ClassyFire [27]) was added (Figure 1(5)), and the previously obtained biological and photophysical data were incorporated (Figure 1(6)). Via the implementation of a new filtering variable, i.e., the “VIS-Signal” (Figure 1(7)), potential photosensitizers were identified by their capability to absorb light. Finally, for all features of interest, the putative annotations were reviewed (Figure 1(8)) based on their corresponding accurate mass and HRMS^2^ spectra as discussed in detail in the last part of this paper.

### 2.2. DMA Assay

**Figure 2:**
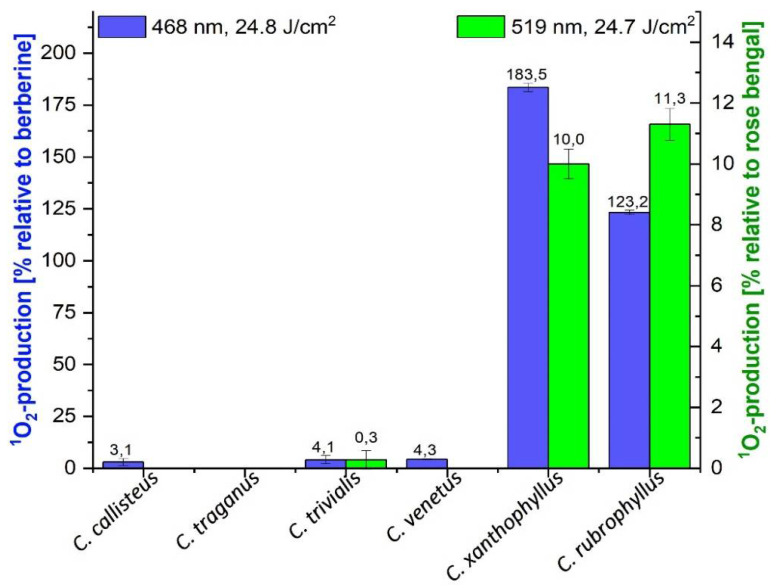
Relative singlet oxygen production of the six investigated fungal extracts calculated by irradiating the samples in ethanol with blue light (λ = 468 ± 27 nm, 1.24 J cm^−2^ min^−1^, berberine = positive control) and green light (λ = 519 ± 33 nm, 1.34 J cm^−2^ min^−1^, rose bengal = positive control). The relative yields are given with standard error.

The DMA assay [7], a low-cost, medium-throughput, photochemical assay, was conducted to rank the fungal extracts according to their photoactivity (Figure 1(1)). The assay allows the ^1^O_2_ formation to be indirectly quantified by quenching the anthracene absorbance at λ = 377 nm through the reaction of 9,10-dimethylanthracene to its peroxy-derivative. Two different light sources were applied to cover a wide range of fungal pigments’ absorption maxima. For photochemical excitation of pigments with yellow coloration, the extracts were irradiated with blue light (λ = 468 ± 27 nm), whereas a green light source (λ = 519 ± 33 nm) was used for red pigments. As reference compounds, the green light-absorbing pink pigment rose bengal (λ_max_ = 555 nm, φ_Δ,EtOH_ = 0.86, InChI key: UWBXIFCTIZXXLS-UHFFFAOYSA-L) [28] and the blue light-absorbing natural photosensitizer berberine (yellow pigment, λ_max_ = 420 nm, φ_Δ,DCM_ = 0.25, φ_Δ,EtOH_ = 0.04) [28,29] were chosen. The obtained results are displayed in detail in Figure 2.

The extracts of *Cortinarius callisteus*, *C. traganus*, *C. trivialis*, and *C. venetus* showed negligible ^1^O_2_ formation values (<5%) independent of the irradiation light source. The *C. xanthophyllus* extract exhibited the highest value (183.5%) followed by *C. rubrophyllus* with 123.2% relative to berberine upon irradiation with blue light. The green light source utilization yielded ^1^O_2_ formation values of 10.0% for *C. xanthophyllus* and 11.3% relative to rose bengal for *C. rubrophyllus*. These low values are consequences of the DMA assay’s relative response and the higher photoactivity of rose bengal (φ_Δ,EtOH_ = 0.86) [28] compared to berberine (φ_Δ,EtOH_ = 0.04) [29]. The wide range of pigments reported for the fruiting bodies of these Cortinarii [16,30,31,32] and both extracts’ intense coloration (*C. rubrophyllus*: auburn; *C. xanthophyllus*: red purple) indicate that photoactivity results from a complex mixture of different secondary metabolites.

### 2.3. (Photo)Cytotoxicity Assay

All fungal extracts were screened for their (photo)cytotoxicity to validate the experimental data from the DMA assay (Figure 1(2)). The PDT-relevant cancer cell lines A549 (non-small cell lung cancer), AGS (stomach cancer), and T24 (urinary bladder carcinoma) were chosen. Tumors of such cancer types can be efficiently irradiated as they are in inner cavities and are thus suitable for PDT treatments [33].

**Figure 3:**
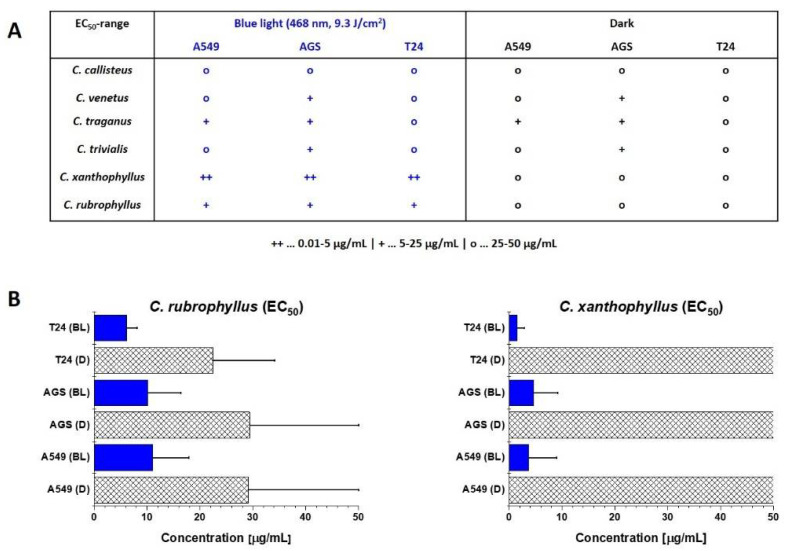
(Photo)cytotoxic activity of the fungal extracts against the cancer cell lines A549, AGS, and T24 in the presence (BL/blue light, λ = 468 ± 27 nm, 9.3 J cm^−2^) and in the absence of blue light (D/dark). Bars: EC_50_ value in µg/mL with the respective confidence interval (95%). (**A**) Results of all six extracts measured as biological duplicates given as EC_50_ ranges (++ … 0.01–5 µg/mL, + … 5–25 µg/mL, o … 25–50 µg/mL). (**B**) Detailed investigation of the most promising extracts (i.e., *C. xanthophyllus* and *C. rubrophyllus*) measured as biological triplicates. A methanolic extract of *B. ilicifolia* showed an EC_50_ of 17 µg/mL under light irradiation (blue light, λ = 468 ± 27 nm, 9.3 J cm^−2^).

All extracts that exhibited a significant level of photoactivity in the DMA-assay were able to induce light-dependent cell death in all three cancer cell lines (Figure 3A, and Supplementary Information (SI) Table S3). Under irradiation, both active extracts were able to kill 50% of the cancer cell populations in a low µg/mL range (EC_50,_*_C.__xanthophyllus_* = 0.01–5 µg/mL, EC_50,_*_C.__rubrophyllus_* = 5–25 µg/mL) (Figure 3A). Moderate cytotoxicity (EC_50_ = 25–50 µg/mL) independent from irradiation was observed for the extracts of *C. callisteus*, *C. venetus*, *C. traganus*, and *C. trivialis*. An extract of the root of *Berberis ilicifolia* containing the natural photosensitizer berberine was utilized as a positive control. This extract showed an activity in the low µg/mL range (e.g., EC_50,A549,irr_ = 17 µg/mL) [7] against cells of the chosen cell line.

In the next step, the extracts of *C. xanthophyllus* and *C. rubrophyllus* were chosen for a more detailed photobiological analysis. As shown in Figure 3B, the *C. xanthophyllus* extract was characterized by very high photocytotoxicity on all three cancer cell lines (EC_50,A549,irr_ = 3.7 ± 5.3 µg/mL (S.I._A549_ = >10.2), EC_50,AGS,irr_ = 4.6 ± 4.5 µg/mL (S.I._AGS_ = >8.1), EC_50,T24,irr_ = 1.5 ± 1.4 µg/mL (S.I._T24_ = >25.3)) with excellent selectivity indices. Hence, the concentration of the extract capable of killing 50% of T24 cancer cells in the presence of blue light was more than 25 times lower (i.e., more efficient) than the concentration showing the same effect in the dark. The *C. rubrophyllus* extract exhibited a light-induced amplification of cytotoxicity on the tested cell lines (EC_50,A549,irr_ = 11.1 ± 6.8 µg/mL (S.I._A549_ = 2.6), EC_50,AGS,irr_ = 10.1 ± 6.3 µg/mL (S.I._AGS_ = 2.9), EC_50,T24,irr_ = 6.1 ± 2.1 µg/mL (S.I._T24_ = 3.7)), but also showed a cytotoxic effect in the absence of light. Microscopical investigations (SI Figures S4–S6) suggested cell death via apoptotic processes as cells treated with the extracts of *C. rubrophyllus* or *C. xanthophyllus* in combination with blue light irradiation were shrunken, nuclei were condensed, and apoptotic bodies were present [34].

Inspired by the promising green light activity of *C. xanthophyllus* and *C. rubrophyllus* in the DMA assay, the decision was made to test the photocytotoxic effect of these two extracts under green light irradiation (λ = 519 ± 33 nm). Green light, as compared to blue light, allows for deeper tissue penetration and lower photocytotoxic side-effects (i.e., side-effects induced by the photoactivation of riboflavin-like pigments occurring in the skin) [35]. In a preliminary experiment with the *C. xanthophyllus* extract, two irradiation times (i.e., t_irr_ = 7.0 and 15.0 min) were compared. As expected, an irradiation time of t_irr_ = 15.0 min (20.1 J cm^−2^) resulted in lower EC_50_ values paired with higher selectivity indices (refer to SI Section 2.2.1 for detailed information).

**Figure 4:**
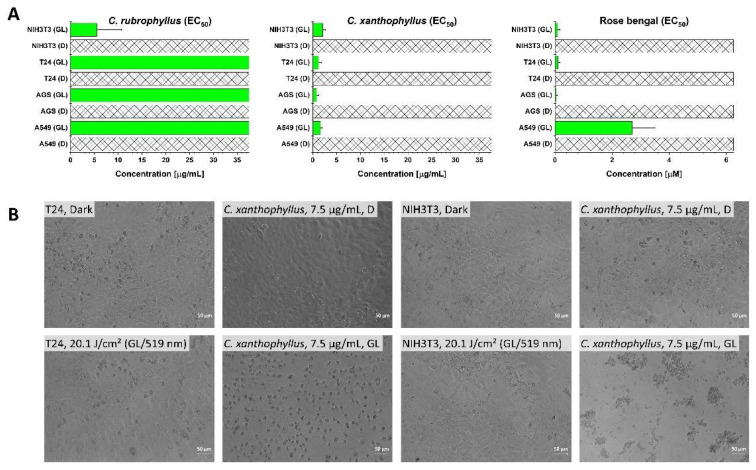
Results of the (photo)cytotoxicity assay employing green light (λ = 519 nm, 20.1 J cm^−2^). (**A**) (Photo)cytotoxic activity of the acetone extracts of *C. rubrophyllus*, *C. xanthophyllus*, and rose bengal against the three cancer cell lines T24, AGS, and A549 as well as against the non-malignant NIH3T3 cell line. Bars: EC_50_ value in µg/mL/µM with the respective confidence interval (95%). (**B**) Micrographs (200× magnification) of cells of the T24 and NIH3T3 cell lines treated (24 h) with the acetone extract of *C. xanthophyllus* (7.5 µg/mL). The upper line of pictures shows treated cells in the dark, the lower after irradiation with green light (519 nm, 20.1 J/cm^2^).

Remarkably, under green light irradiation, the *C. xanthophyllus* extract was able to induce cell death in an extremely low concentration range and with high selectivity (EC_50,A549,irr_ = 1.6 ± 0.4 µg/mL (S.I._A549_ ≥ 23.4), EC_50,AGS,irr_ = 0.8 ± 0.5 µg/mL (S.I._AGS_ ≥ 46.9), EC_50,T24,irr_ = 1.2 ± 0.6 µg/mL (S.I._T24_ ≥ 32.3)). To determine if an additional selectivity exists between cancerous and non-malignant cells, we included a test against cells of the mouse fibroblast cell line NIH3T3. The results of the *C. xanthophyllus* extract (EC_50,NIH3T3,irr_ = 2.1 ± 0.6 µg/mL (S.I._NIH3T3_ ≥ 17.9)) showed, however, only a slight, non-significant difference between the cell types. Holding true for both the *C. xanthophyllus* extract and rose bengal, the morphological changes induced by the green light treatment showed clear signs of a programmed cell death (Figure 4B, SI Figures S7–S10). This underlines the selectivity potential of light activation and further suggests the presence of highly photocytotoxic red-colored pigments (absorbance peak in the green light spectral region), which could rival the photodynamic potential of the showcase natural PS hypericin [36,37].

The acetone extract of *C. rubrophyllus*, however, failed to selectively kill the cells of the cancer cell lines upon green light irradiation in the tested concentration range (Figure 4A), despite having shown a higher singlet oxygen formation value in the DMA assay. This might be due to an insufficient cellular uptake of the pigments responsible for the green light activity. Against cells of the slower growing non-malignant cell line NIH3T3, a selectivity was observed.

Considering the results from the cell culture experiments, it can be assumed that highly photocytotoxic pigments are present in the acetone extracts of *C. xanthophyllus* and *C. rubrophyllus*. The investigation of plant extracts with comparable EC_50_ values (i.e., EC_50_ < 3 µg/mL), by the means of bioactivity-guided isolation, frequently leads to the discovery of promising cytotoxic compounds [38,39,40,41,42]. Thus, it can be assumed that both *Cortinarius* species are great starting points for the discovery of novel photocytotoxic compounds.

### 2.4. Feature-Based Molecular Network

#### 2.4.1. Generation and General Investigation

Feature-based molecular networking (FBMN) [20] represents a powerful processing method to visualize and annotate complex, high-resolution untargeted data-dependent LC-MS/MS metabolite profiling analyses of natural extracts. The terminology used in this paragraph is thoroughly discussed by Aron and colleagues in their work on reproducible molecular networking of untargeted mass spectrometry data using Global Natural Products Social Molecular Networking (GNPS) [43].

After data generation (refer to SI Section 3 for details, Figure 1(3)) and conversion [44], the resulting FBMN was visualized with Cytoscape (Figure 1(4)) [45] and characterized regarding general aspects, such as the number of nodes, edges, and clusters, the specificity of features, the annotation-hit-rate using different spectral databases, the chemical taxonomy, and the polarity of the active clusters (SI Section 3.4). The network (negative ionization mode, SI Section 3.3 for experimental details) comprised 3745 individual nodes and 4643 edges. The nodes were gathered into 461 different clusters. The number of self-loops (singletons) was 1920. GNPS spectral libraries (i.e., experimental MS^2^ data) and in silico fragmentation spectra generated from the Dictionary of Natural Products (ISDB-DNP [25]) were used to interpret the recorded mass spectra of the FBMN. The resulting candidate annotations were re-ranked using the script for taxonomically informed metabolite annotation [26], which also contained the taxonomical information for the species under investigation (Figure 1(5)). The ClassyFire chemical taxonomy [27] was automatically assigned to all the candidates. This output was used to obtain a holistic view of the extracts’ chemical composition (SI Section 3.4.8). Thereby, the chemical entities detected by UHPLC-MS^2^ metabolite profiling could be comprehensively organized into structural classes. The results revealed that 268 features of all nodes could be putatively classified as “Fatty acyls”, 160 features as “Benzene and substituted derivatives”, 153 features as “Organooxygen compounds”, 111 features as “Prenol lipids”, and 59 features as “Anthracenes”. The remaining features were scattered across different classes and amounted to less than 50 each.

As all possible features within the active extracts could be considered photoactive (Figure 1(6)), it was important to distinguish the actual active principles responsible for the observed photoactivity from the inactive ones. For this purpose, a photochemistry-based variable was included in the workflow. According to the first law of photochemistry (i.e., Gotthus–Draper law), only compounds capable of absorbing light can be considered potential photosensitizers. Thus, a variable was defined which provides features showing an absorption in the visible spectral range (λ = 468 nm) with a “1” (positive “VIS-Signal” variable/high probability of photoactivity) and features lacking this ability with a “0” (negative “VIS-Signal” variable/photoinactive) (SI Section 3.4.1) (Figure 1(7)).

The specificity of features was investigated on the following levels: >60% (major occurrence), >95% (virtual specificity), and >99% (complete specificity) (SI Section 3.4.5). The specificity of a feature was calculated as the LC peak area per extract divided by the sum of the LC peak areas of the same peak in all extracts. The extracts of *C. callisteus*, *C. traganus*, and *C. xanthophyllus* contained the greatest number of specific features on all three levels. This correlates positively with the fact that these species belong to independent, distinct, and phylogenetically distant lineages of *Cortinarius* [46]. Considering the features’ ability to absorb visible light (“VIS-Signal” variable, SI Section 3.4.1), solely *C. rubrophyllus* and *C. xanthophyllus* exhibited specific features. The number of specific (>99% level), light-absorbing features was nine for *C. rubrophyllus* and 52 for *C. xanthophyllus*. Thus, a total of 61 potentially photoactive features were found and were specific to the active extracts.

By overlaying the chemical taxonomy information with the findings from the photochemical/biological evaluation and the “VIS-Signal” variable, compound classes associated with photoactivity were selectively highlighted in the FBMN (SI Section 3.4.9). This investigation revealed that the chemical families “Anthracenes”, “Benzene and substituted derivatives”, and “Prenol lipids” comprise most features, which were present in photoactive extracts and capable of absorbing light in the visible range (λ = 468 nm). Since numerous anthraquinones and aromatic compounds are known for their ability to produce singlet oxygen after irradiation (e.g., aloe-emodin, hypericin, and phenalenone) [47,48,49,50,51], and some are even commonly used in PDT and PDT research, these chemotaxonomical results are promising for the subsequent in-depth annotation process.

#### 2.4.2. Photoactive-Feature Annotation Overview

**Figure 5:**
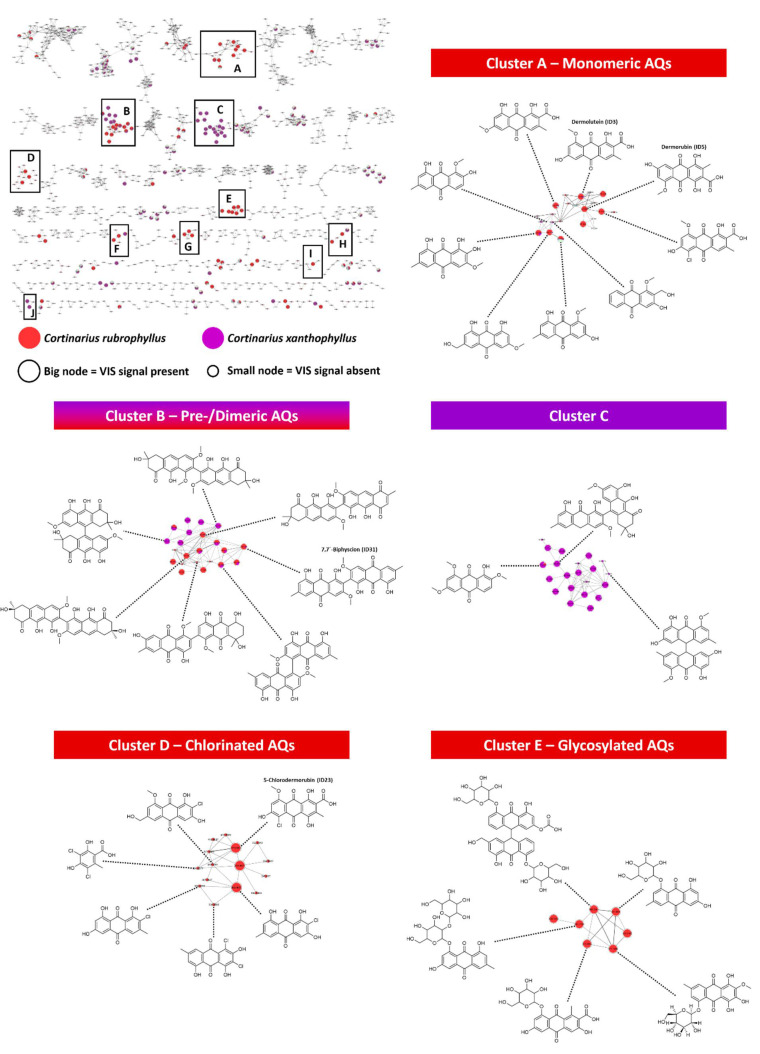
The FBMN with the marked photoactive clusters (A–J) (top left corner) as well as the annotation results for the clusters A–E. Features specific for the active extracts are highlighted as colored nodes: *C. rubrophyllus* = red, *C. xanthophyllus* = purple. The node size depicts the features’ ability to absorb visible light: big = “VIS-Signal” present, small = “VIS-Signal” absent. The marker compounds (i.e., dermolutein (ID3), dermorubin (ID5), 5-chlorodermorubin (ID23), and 7,7′-biphyscion (ID31)) are provided with a name on top of the molecular structure. Identification levels are as follows: marker compounds = 1, all other structural suggestions = 2. Refer to the SI for more details of all investigated clusters.

Ten different clusters (A–J) (SI Figure S12) containing features from *C. xanthophyllus* and *C. rubrophyllus* were highlighted with “VIS-Signal” positive nodes and subsequently submitted to a feature annotation workflow (SI Sections 3.2, 3.3, and 3.4.3). This was achieved based on spectral matching against the GNPS library [20] and an in silico database (ISDB-DNP) [25], in-house library dereplication, and the use of the in silico annotation tool Sirius 4.4.29 [24,52]. Briefly, all clusters’ putative annotation was performed by combining MS^2^-based spectral hits with taxonomical information (ISDB-DNP-Taxo and “Cortinariaceae” variable; SI Section 3.4.4) and evaluation of structural consistency (i.e., scaffold and substitution pattern) in the FBMN clusters. Molecular structures were selected hierarchically, whereby the conformity of the annotation results (spectral matching against GNPS, ISDB-DNP, and an in-house library) was more trusted than the result of solely the in-house library (fungal origin) or solely the in silico annotation. Anthraquinone-rich fractions from a previous study (i.e., the mycochemical investigation of *C. uliginosus* [8]) were integrated into the FBMN to attain the marker compounds dermolutein (ID3), dermorubin (ID5), 5-chlorodermorubin (ID23), and 7,7′-biphyscion (ID31) (Figure 5). By manually propagating the structural information provided by these markers, the dependability of the annotation for features clustering with these compounds can be greatly increased.

Since each annotation strategy generated many structural hits, the annotation process’ predictive power was limited. Additional taxonomical and genus-specific chemical knowledge was required to select meaningful spectral matches, despite the significant improvement of annotation reliability via taxonomically informed metabolite annotation [26]. To highlight this limitation, up to four spectral hits (ranked as described below) represented as their respective SMILES were listed in Tables S5–S14 (SI Sections 3.4.4.1.1–3.4.4.1.10). The four levels of accuracy reported in the Metabolomics Standard Initiative were used to describe the confidence level achieved in the feature annotation (i.e., marker compounds = 1, all other putative annotations = 2, Tables S5–S14) [53]. Spectral matching of MS^2^ spectra against the GNPS spectral library led to 148 hits (4.0%), whereas the taxonomically informed metabolite annotation against in silico ISDB-DNP afforded 2269 putative annotations (60.6%; relative to the number of nodes in the FBMN) (SI Section 3.4.6). Molecular networking studies in the field of mycochemical research and thus publicly available mass spectrometry data originating from fungal secondary metabolites could improve the GNPS-associated annotation hit rate as well as the overall annotation process.

#### 2.4.3. Specificity of Features and Phylogenetic Relevance

According to Figure 5, the active clusters A, D, E, F, G, H, and I were specific for *C. rubrophyllus* and putatively identified as clusters comprising monomeric and dimeric anthraquinones (AQs), as well as their chlorinated (confirmed by the isotopic pattern, SI Figures S32–S35), glycosylated, methylated, and esterified derivates (SI Section 3.4.4). Clusters F and H were also partly specific for *C. xanthophyllus*. Cluster B was specific for *C. rubrophyllus* and *C. xanthophyllus* alike, pointing towards the possibility of a shared evolutionary history of those two species based on their secondary metabolite profiles. For many years, chemotaxonomy was considered the key to resolving evolutionary relationships but was later replaced by molecular methods (e.g., the investigation of sequences of the rDNA internal transcribed spacer (ITS) or the rDNA large subunit (LSU)) [54]. However, it was impossible to resolve basal phylogenetic relationships in the extremely species-rich genus *Cortinarius*, although it represents one of the best-studied genera of Agaricomycetes (Basidiomycetes) and was addressed based on several multigene phylogenetic studies [46,55]. For example, rDNA ITS sequences of *C. claroflavus* and *C. xanthophyllus* are identical [56], a fact highlighting the limited resolution of molecular phylogenies based on rDNA sequences only. These two species can be clearly distinguished based on basidiome pigmentation and different macrochemical color reactions.

Furthermore, an rDNA-based tree topology including hundreds of *Cortinarius* species indicates that Phlegmacia (including *Calochroi*; *C. xanthophyllus*) might be more closely related to *Dermocybe* (*C. rubrophyllus*) than previously assumed [55]. Both groups of Cortinarii include representative species with the rather untypical feature of brightly colored basidiomes (yellow, red), while the majority of *Cortinarius* species have brown, only rarely blue basidiomes. Therefore, investigating fungal secondary metabolites with the help of novel metabolomics tools such as FBMN might add an additional level to the evolutionary history of this important group of fungi. Genome studies would certainly provide important information on genes involved in pigment biosynthesis. However, only one fully sequenced genome of a representative *Cortinarius* species is currently available. The *C. glaucopus* genome is comparatively large (20k proteins (https://www.ncbi.nlm.nih.gov/genome/96691, accessed on 15 November 2021). Explorative studies including the complete genome of all involved *Cortinarius* species are needed to investigate pigment biosynthesis and its evolutionary history in these fungi in more detail.

#### 2.4.4. Cluster Annotation and Bioactivity Prioritization

In general, features that exhibited a signal in the visible spectral region (λ = 468 nm) in the UHPLC chromatogram were also putatively identified as colored compounds (e.g., anthraquinones (AQs)) and thus provided with meaningful structural suggestions.

Throughout Cluster A, features were putatively annotated as monomeric AQs with a high degree of homogeneity (Figure 5, SI Table S5). Based on matching MS^2^ spectral data and the use of authentic reference compounds, two features were identified as the AQs dermolutein (ID3, Cluster A) and dermorubin (ID5, Cluster A). Both exhibited light-induced activity (i.e., production of ^1^O_2_) [8]. These AQ-carboxylic acids are known secondary metabolites of dermocyboid Cortinarii [9], but are unlikely to contribute to its photocytotoxic effect due to their insufficient cellular uptake [8]. Furthermore, the presence of various methylated and esterified monomeric AQs in this species can be anticipated, as the annotation results for Cluster H (SI Figure S21) suggest.

Cluster B was putatively annotated as a cluster comprising pre-AQs and dimeric AQs (Figure 5, SI Section 3.4.4.1.2). Due to the features’ ability to absorb visible light (positive “VIS-signal” variable) combined with their putative annotation as dimeric AQ-like structures, it seems likely that these features contribute to the photoactivity of both extracts. This hypothesis is further supported by the recent discovery of the light-dependent cytotoxicity of the dimeric AQ 7,7′-biphyscion and its ability to induce apoptosis in lung cancer cells under blue light irradiation [8]. The putative annotation of flavomannin-type and AQ pigments in the *C. rubrophyllus* extract is consistent with literature data documenting the occurrence of these compounds in related *Dermocybe* species [9]. Furthermore, the presence of those very pigments belonging to the flavomannin group (e.g., flavomannin-6,6′-di-O-methyl ether (FDM)), which are considered as precursors of dimeric AQs [16] (i.e., pre-AQs), might also result in the dark cytotoxic effect detected in the cell culture experiments. Pachón-Peña and colleagues were able to demonstrate the dose-dependent growth inhibition of FDM on the proliferation of human adenocarcinoma colorectal cells Caco-2 [57]. Moreover, dimeric AQs (e.g., 7,7′-biphyscion (ID31) in Cluster B, Figure 5) in the *C. rubrophyllus* extract are probably the active principle responsible for the blue light-induced cytotoxicity on the tested cell lines [8]. Since features entirely specific for *C. xanthophyllus* were gathered into the same molecular family as the flavomannin-type pigments (Cluster B, Figure 5), one could falsely assume the occurrence of AQ pigments linked at position 7 (i.e., 7,7′-dimeric AQs) in this fungus. However, *C. xanthophyllus* is rather known to produce a certain class of pigments characteristic to the subgenus *Phlegmacium*, namely, AQs of the phlegmacin group (7,10′-coupling) [16,58,59,60,61]. Therefore, it is more likely, that this cluster comprises structural isomers of flavomannins (e.g., 7,10′- or 10,10′-dimeric AQs). The absence of flavomannin-like (7,7′-coupling) compounds in the *C. xanthophyllus* extract could also be the explanation for its lower dark cytotoxic effect.

Interestingly, the annotation workflow failed to deliver meaningful results for Cluster C (SI Figure S16), which is entirely specific to *C. xanthophyllus* and consists of mainly visible light-absorbing features. Of the 21 features, only three were provided with meaningful structural suggestions (one monomeric AQ, one pre-AQ, and one dimeric AQ; SI Table S7). Since one feature was putatively identified as anhydrophlegmacin, propagation of this structural information throughout the cluster suggests the presence of other compounds with this scaffold, rather than the class of “Prenol lipids”, which was proposed by the chemical taxonomy investigation. The low annotation hit rate for this cluster might come from the fact that the subgenus *Phlegmacium*, as well as the genus *Cortinarius* in general, are relatively unexplored by mass spectrometry-based bioinformatics tools. Thus, the extract’s prominent cytotoxicity after green light irradiation cannot be explained entirely based on the current data. However, dimeric AQs or AQ-like compounds, which are structurally related to the phototoxic red pigment hypericin, are most likely responsible for this activity.

In Cluster D (Figure 5, SI Table S8) some features without a “VIS-Signal” were putatively annotated as chlorinated AQs (SI Figures S32–S35). As those features were only present in a low concentration (i.e., low signal intensity/peak area) in the extracts, they were not given a positive value by our “VIS-Signal” variable generation workflow. Therefore, despite being represented as small nodes, the putative identification of those features as chloro-substituted AQs is still valid. Currently, the photodynamic potential of chlorinated AQs is obscure; thus, future scientific efforts should be directed towards the photochemical/biological characterization of those compounds.

The features of Cluster E (Figure 5, SI Figure S18) and Cluster G (SI Figure S20, Table S11) were putatively annotated as glycosylated AQs. Especially *Cortinarius* spp. of the classical subgenus *Dermocybe*, such as *C. malicorius*, are known to produce various glycosylated derivatives of their main pigments [9]. However, their general bioactivity as well as their photoactivity remain unclear.

The annotation of features belonging to Cluster F (SI Figure S19) yielded the structures of dimeric AQ-like compounds of the Alterporriol-type, which are known secondary metabolites of endophytic fungi (e.g., *Alternaria* sp. and *Stemphylium globuliferum*) [62,63]. However, Alterporriol-type compounds have not been described for either *C. rubrophyllus* or *C. xanthophyllus*. Nevertheless, the annotation results for Cluster F entail one promising aspect: structures with similar scaffolds are present in endophytic fungi. In contrast to Cortinarii, which form mycorrhizal associations with ectotrophic trees [64], endophytic fungi can be cultivated easily on rice, for example [63]. Therefore, potentially bioactive dimeric AQs and AQ-like compounds from Agaricomycetes could become readily available as they or their structurally related compounds could be isolated from cultivatable fungi, eliminating the problem of limited amounts of biomaterial.

Since one feature of Cluster I was suspected to be the known photosensitizer emodin (SI Table S13) [65,66,67] by GNPS spectral matching and in-house library dereplication, it can be anticipated that this or similar compounds might partly account for the photoactivity of the *C. rubrophyllus* extract.

The group of phlegmacin-type compounds, which is characteristic for the classical subgenus *Phlegmacium* [16], is represented by Cluster J (SI Figure S23). There, two features were annotated as anhydrophlegmacin and anhydrophlegmacin-9,10-quinone-8′-O-methyl ether. However, the occurrence of these pigments in *C. xanthophyllus* has not yet been proven [16]. Apart from the melanogenesis inhibiting potential of phlegmacin-type compounds isolated from *Cassia auriculata* seeds and their missing cytotoxicity on human dermal fibroblasts [68], biological activities of this AQ class remain mostly unknown. Yang et al. reported a good application prospect of phlegmacin B1 and A1 in the prevention and treatment of agricultural pests because of their ability to efficiently inhibit the activities of chitinase OfChi-h and hexosaminidase OfHex1 [69]. Thus, a photochemical and biological investigation of AQs belonging to the phlegmacin group could afford the discovery of promising pharmaceutical leads for PDT.

#### 2.4.5. FBMN—Final Specific Remarks

To sum up, combining in vitro photochemical and biological data with state-of-the-art FBMN made it possible to visualize the fungal extracts’ chemical space while simultaneously extrapolating their underlying photoactive principle. Anthraquinones (AQs) were discovered as markers for photoactivity in the investigated fungal extracts. Features specific for the photocytotoxic extracts of *C. rubrophyllus* and *C. xanthophyllus* were putatively annotated as AQs with diverse substitution patterns (i.e., monomeric, dimeric, glycosylated, methylated, esterified, and chlorinated). Considering the annotation results for features mainly present in *C. xanthophyllus*, the annotation workflow suggested (Identification level 3) yet unknown fungal photosensitizers, and thus potentially new natural products. To reveal their origin, a thorough mycochemical investigation of *C. xanthophyllus* is necessary. Unfortunately, this is hindered by the rare occurrence of this species (only 110 records on the Global Biodiversity Information Facility (GBIF; https://www.gbif.org/species/2528874; accessed 15 November 2021); listed on the Swedish Red List of threatened species [18]). Further studies on the metabolites of *C. xanthophyllus* are planned but can only be carried out as soon as sufficient reliably identified voucher material is available. The high sensitivity of the metabolite profiling by UHPLC-MS^2^ and FBMN should allow for the identification of other biological sources containing similar metabolites by applying the approach developed and comparing the data with those obtained for *C. xanthophyllus*.

### 2.5. Outlook

Utilizing the knowledge gained from bioactive natural products prioritization [21] via the combination of indirect singlet oxygen quantification (i.e., DM -assay), evaluation of light-induced cytotoxicity, and FBMN in the field of mycochemistry, future efforts should be directed towards the following topics: (i) understanding of the role of fungal secondary metabolites in chemical defense, (ii) resolving the evolutionary history of the genus *Cortinarius* via the combination of bioinformatics-based chemotaxonomy and molecular phylogenetics, and (iii) the discovery of novel photopharmaceuticals. FBMN highlighted apparent similarities in the chemical composition of *C. rubrophyllus* and the previously investigated species *C. uliginosus* (*Dermocybe*) because both contain similar photoactive AQs. Thus, the *Dermocybe* group seems to be highly promising for a general investigation of photoactivity as a defense strategy (e.g., in vivo studies investigating the biological effect of photoactive AQs on insects or parasites or worms). Furthermore, our results showed that *C. xanthophyllus* is an exciting source for novel photosensitizers suitable for PDT. However, the drawback of its rarity still obstructs an in-depth mycochemical analysis. Nevertheless, FBMN proved to be a very exciting tool to find new natural photosensitizers.

## 3. Materials and Methods

### 3.1. General Instrumentation

Fungal biomaterial was dehumidified with a Dörrex^®^ drying-apparatus from Stöckli (A. & J. Stöckli AG, Netstal, Switzerland) operated at a temperature of 50 °C. Weighing of samples was done with the weighing instruments KERN ALS 220-4 (KERN & SOHN GmbH, Balingen-Frommern, Germany) and Sartorius CUBIS^®^ series equipment (Sartorius AG, Göttingen, Germany). Ultrasonic extraction was performed with the ultrasonic bath Sonorex RK 52 (BANDELIN electronic GmbH & Co. KG, Berlin, Germany). The power adaptor Agilent E3611A DC Power Supply (Agilent Technologies, Inc., Santa Clara, CA, USA) in combination with two LED-panels (λ = 468 ± 27 nm (1.24 J cm^−2^ min^−1^) and λ = 519 ± 33 nm (1.34 J cm^−2^ min^−1^)) (University Leiden, published in Hopkins et al. [70]) was utilized for the DMA assay. Absorption measurements were done with the plate reader Tecan Spark^®^ 10 M (Tecan Group Ltd., Männedorf, Switzerland). Adjustment of pH values was carried out with the Mettler Toledo Seven Multi pH meter (Mettler Toledo GmbH, Vienna, Austria). The vortex mixer Vortex-Genie 2 (Scientific Industries, Inc., Bohemia, NY, USA) was employed and the pipettes, as well as tips, were either from Eppendorf AG (Hamburg, Germany) or from STARLAB International GmbH (Hamburg, Germany). Reagent reservoirs were obtained from Thermo Fischer Scientific (Waltham, MA, USA). Specific instruments are listed in the respective sections.

### 3.2. Chemicals

All solvents for the extraction procedures and thin-layer-chromatographic analyses were sourced from VWR International (Vienna, Austria). Acetone was distilled prior to use. Solvents used for HPLC experiments were at least pro analysis (p.a.) quality and were purchased from Merck (Merck KGaA, Darmstadt, Germany). Ultrapure water was obtained via the Sartorius arium^®^ 611 UV purification system (Sartorius AG, Göttingen, Germany). The reagents 9,10-dimethylanthracene (product number: D0252), acid red 94 (rose bengal, product number: R0041), and acid red 52 (sulforhodamine B, product number: A0600) were sourced from TCI Deutschland GmbH (Eschborn, Germany). Expendable materials (e.g., flasks), media, and supplements (i.e., FCS, penicillin/streptomycin, trypsin, PBS) used for cell culture maintenance and the (photo)cytotoxicity assay were purchased from Thermo Fischer Scientific (Waltham, MA, USA).

### 3.3. Phylogenetic Analysis

Data analysis was carried out based on rDNA ITS sequences. For this fungal barcoding region, the best reference database available was used, including sequences from holotypes. Reference sequences from the most closely related species were downloaded from GenBank, by restricting the search to type sequences only, as far as possible. A total of 60 rDNA ITS sequences were aligned and manually adjusted in MEGA X [71]. The evolutionary history was inferred by using the maximum likelihood method based on the Hasegawa–Kishino–Yano model +G, parameter = 0.2332. All positions with less than 95% site coverage were eliminated. To evaluate the robustness of the branches in the phylogenetic trees, parsimony-based bootstrap analyses were applied. The bootstrap analyses were conducted using 500 replications, the SPR search method, and search level 5. The tree was drawn using InkSpace 1.0.2 (e86c8708, 15 January 2021).

### 3.4. Fungal Material

Voucher specimens of all *Cortinarius* species (see also SI) are deposited in the Natural Sciences Collections of the Tiroler Landesmuseen (IBF), Krajnc-Straße 1, 6060 Hall, Austria (http://www.tiroler-landesmuseen.at, accessed on 15 November 2021) and were provided for mycochemical analysis.

### 3.5. Sample Preparation and Extraction Procedures

The hot air-dried (50 °C) fruiting bodies of *Cortinarius callisteus*, *C. rubrophyllus*, *C. traganus*, *C. trivialis*, *C. venetus*, and *C. xanthophyllus* were ground with mortar and pestle to yield fine powders and stored separately in small paper bags. Ultrasonic extraction of the powdered biomaterials was performed under the exclusion of sunlight at room temperature (22 °C). In detail, ground fruiting bodies (approx. 2× *g* each) were extracted (10 min) with acidic acetone (5 mL, 1% HCl). The extracts were centrifuged (5 min, 14,000 rpm = 20,817× *g*, 4 °C) and decanted. This procedure was done three times and the supernatants were combined. The extracts were dried in the dark under an air stream and kept in a desiccator before use.

### 3.6. Feature-Based Molecular Networking (FBMN)

Please refer to the electronic Supplementary Information (SI Section 3).

### 3.7. DMA Assay

For the DMA assay, two stock solutions, an ethanolic DMA solution (1.4 mM) (S1) and an L-ascorbic acid solution (100 mM, pH = 7.0–7.4) (S2), were prepared. Using the stock solutions and pure ethanol (S3), four working solutions (i.e., pure ethanol, a DMA solution ((S1) 1250 µL + (S2) 3750 µL) an L-ascorbic acid solution ((S2) 500 µL + (S3) 4500 µL), and a mixture of DMA and L ascorbic acid-solution ((S1) 1250 µL + (S2) 500 µL + (S3) 3250 µL)) were generated. First, solutions of the fungal extracts (1 mg/mL, DMSO, 10 µL) were pipetted in a 96-well plate. Then, the four working solutions were added (190 µL in each well). DMSO (10 µL) was used as a negative control and berberine (1 mg/mL, 2.97 mM, DMSO, 10 µL) and rose bengal (0.1 mg/mL, 0.10 mM, DMSO, 10 µL) were used as positive controls. Thereafter, optical densities at the wavelengths 377 nm, 468 nm, and 519 nm were measured with a plate reader (t = 0 min), followed by four cycles of irradiating the plate with blue light (λ = 468 nm, 1.24 J cm^−2^ min^−1^, berberine = positive control) for five minutes or with green light (λ = 519 nm, 1.34 J cm^−2^ min^−1^, acid red 94 = positive control) for 4.6 min. All measurements were done as technical duplicates. The singlet oxygen production was calculated relative to berberine/rose bengal with the formula described previously [7]. The results of the DMA assay were presented as the mean ± standard error.

### 3.8. Cell Culture Maintenance and (Photo)Cytotoxicity Assay

Cells of the adherent cancer cell lines A549 (non-small lung cancer, ATCC, Merck KGaA, Darmstadt, Germany), AGS (stomach cancer, CLS, Eppelheim, Germany), T24 (urinary bladder carcinoma, CLS, Eppelheim, Germany), and of the mouse embryonic fibroblast cell line NIH3T3 (ATCC, Manassas, Virginia, CRL 1658) were cultivated in Nunc EasYFlasks (product number: 51985042, 75 cm^2^) with Gibco™ MEM™ medium (product number: 42360081) supplemented with fetal calf serum (FCS, 10% *v*/*v*) and penicillin/streptomycin (P/S, 1% *v*/*v*). Cells were trypsinized when reaching 70–80% confluency and used for approximately 8–12 weeks. Freezing and thawing of cell cultures were done according to standard procedures. Microscopic investigations were done employing a Leica DMi1 microscope (Leica, Wetzlar, Germany). A 10× objective was used and a 10× ocular, as well as a digital, zoom. The (photo)cytotoxicity assay was conducted as published previously [70].

Briefly, cells (AGS: 2500 cells/well, T24 and A549: 2000 cells/well, NIH3T3: 4000 cells/well) were seeded in Gibco™ Opti-MEM™ (OMEM, product number: 11058021) containing FCS (2.4% *v*/*v*) and P/S (1% *v*/*v*) at 37 °C in 5% CO_2_ atmosphere. Firstly, to spot general photocytotoxicity, the fungal extracts of the six selected Cortinarii were dissolved in DMSO (stock solutions: 10 mg/mL) and then further diluted with OMEM. Then, 24 h after seeding the cells, they were treated with the working solutions (100 µL, final concentrations: 5, 25, and 50 µg/mL) of each extract and incubated for another 24 h. Subsequently, the medium was aspirated and replaced with fresh OMEM (+2.5% *v*/*v* FCS, +1% *v*/*v* P/S). After that, the respective plates were irradiated for 7.5 min with blue light (λ = 468 nm, 9.3 J cm^−2^). For experiments that used a green light source (λ = 519 nm), an irradiation duration of 15.0 min was chosen (20.1 J cm^−2^). The cells were fixed by gently adding cold trichloroacetic acid (10% *w*/*v* in water, 100 µL) 48 h after the irradiation step (total experiment time = 96 h) and stored in a refrigerator at 8 °C for at least 24 h. The fixed cell-monolayers were washed with slow running deionized tap water and stained with sulforhodamine B (SRB) (V = 100 µL, acid red 52, 0.4% *w*/*v* SRB in 1% *v*/*v* acetic acid) for 30 min. Thereafter, the plates were washed again (5 times, 1% *v*/*v* acetic acid) and dried at room temperature. Then, tris(hydroxymethyl)aminomethane solution (V = 100 µL, TRIS, 10 mM in water) was added to dissolve the dried dye and incubated for at least 20 min. Absorbance was measured at λ = 540 nm with a plate reader. EC_50_ values including their confidence intervals (95%) were calculated with GraphPad Prism 5 employing the relative Hill slope equation. Based on their respective EC_50_ values, different levels of (photo)cytotoxicity were defined for the fungal extracts: >20 µg/mL = no/low (photo)cytotoxicity, 5–20 µg/mL = high, <5 µg/mL = very high. The selectivity indices (S.I.), expressing the ratio of cells killed in the absence of light versus cells killed upon irradiation, were calculated from the EC_50_ values by use of Formula (1).

(1) $$S.I.={\mathrm{EC}}_{50|\mathrm{dark}}÷{\mathrm{EC}}_{50|\mathrm{irradiated}}$$

After the first experiment was conducted as biological duplicates for two consecutive weeks, the extracts of *C. xanthophyllus* and *C. rubrophyllus* were selected for further investigation. Additional working solutions (final concentrations: 37.500, 18.750, 7.500, 3.750, 1.875, and 0.375 µg/mL) were tested as biological triplicates for three consecutive weeks as described above. For the green light experiments, an additional cell line was tested (i.e., NIH3T3) and a positive control for photocytotoxicity (i.e., rose bengal) in the respective wavelength band (λ = 519 nm) was included. The complete results of the (photo)cytotoxicity investigation, as well as micrographs of the cell lines, are presented in the electronic Supplementary Information (Section 3).

## 4. Conclusions

Just recently, photoactivity was discovered in the division Basidiomycota (regnum Fungi) [7,8]. However, the general occurrence of photoactive pigments, their photopharmaceutical potential, and their ecological role are still rather unclear. In an attempt to explore this phenomenon, this study looked into the chemical space of extracts derived from Cortinarius species representing different phylogenetic lineages for the first time by means of UPLC-HRMS^2^ metabolomics tools (i.e., FBMN) [26,72] to facilitate a holistic view of the complex mixture of fungal secondary metabolites. By implementing in vitro data in the generated network, an elegant way to visualize clusters of photoactive features was developed specifically.

A comprehensive annotation workflow led to the annotation of several AQs specific to the photoactive extracts of *C. rubrophyllus* and *C. xanthophyllus.* The question of whether photoactivity is a generic phenomenon in the genus *Cortinarius* or is only found in dermocyboid Cortinarii, cannot be justified entirely based on the current data. As photochemically and biologically active AQs were found in a fungus belonging to the subgenus *Phlegmacium* as well, it can be concluded that this trait is neither restricted to a single subgenus alone nor found in all subgenera. The results instead suggest that the presence of anthraquinones and structural analogs in fungal extracts involves photochemical and biological activity. Because this work examined only a small subset of the genus *Cortinarius*—one of the largest genera of gilled basidiomycete fungi with approximately 2000 different species [54]—many questions remain unanswered: the ecological role of photoactive AQs as a chemical defense strategy against insects and pests, the suitability of fungal photosensitizers for PDT, and the potential of FBMN as a sophisticated chemotaxonomy approach for aiding the elucidation of the genus’ evolutionary history.

## Data Availability

ITS sequences of the freshly collected species were submitted to the GenBank (refer to SI for details). The FBMN can be downloaded via https://gnps.ucsd.edu/ProteoSAFe/status.jsp?task=ff9b52921d0a4867b81b7373de209a68, accessed on 15 November 2021. The raw data is available via MASSIVE (https://massive.ucsd.edu/ProteoSAFe/static/massive.jsp) ftp://MSV000088332@massive.ucsd.edu or [doi:10.25345/C5TK3Z], accessed on 15 November 2021.

## Supplementary Materials

The following are available online at https://www.mdpi.com/article/10.3390/metabo11110791/s1, Figure S1: Phylogenetic placement of the six investigated *Cortinarius* species. Figure S2: UV/Vis-spectra of the fungal extracts in ethanol. Figure S3: Investigation of the green light-dependent cytotoxicity of the *C. xanthophyllus* acetone extract under two different irradiation durations. Figures S4–S11: Micrographs of treated cells. Figures S12 and S13: FBMN with highlighted active clusters and a legend with the color code used. Figures S14–S23: Annotated active clusters. Figures S24–S27: Bar plots and charts regarding general aspects of the FBMN (i.e., specificity of features and annotation-hit-rate). Figure S28: The FBMN with features related to *Cortinarius* species highlighted as big red dots based on the findings from the ISDB-DNP spectral annotation process. Figure S29: FBMN including the chemical taxonomy (ClassyFire) information (pie chart and network representation). Figure S30: FBMN with highlighted photoactive compound classes. Figure S31: Visual representation of the active clusters’ polarity in the liquid-chromatographic experiment. Figures S32–S35: Mass spectra of putatively annotated chlorinated anthraquinones. Table S1: *Cortinarius* collections used in this study with respective voucher numbers and collection data. Table S2: Extract yields. Table S3: Results of the (photo)cytotoxicity screening of the acetone extracts of *Cortinarius callisteus*, *C. venetus*, *C. traganus*, *C. trivialis*, *C. xanthophyllus*, and *C. rubrophyllus* using blue light irradiation. Table S4: Results of the (photo)cytotoxicity assay of the acetone extracts of *Cortinarius xanthophyllus* and *C. rubrophyllus* employing green light irradiation. Tables S5–S14: Annotation results for the active clusters.
