# Supplementary material for: Feature-Based Molecular Networking—An Exciting Tool to Spot Species of the Genus Cortinarius with Hidden Photosensitizers
Source: Metabolites. 2021 Nov 19;11(11):791. doi: 10.3390/metabo11110791 (PMC8619139; doi:10.3390/metabo11110791)
Supplement: Supplementary file 1 [file metabolites-11-00791-s001.zip › metabolites-1459374-supplementary.pdf]

Supplementary Information

Feature-Based Molecular Networking—An Exciting Tool to Spot Species of the Genus *Cortinarius* with Hidden Photosensitizers

Fabian Hammerle [1], Luis Quirós-Guerrero [2,3], Adriano Rutz [2,3], Jean-Luc Wolfender [2,3], Harald Schöbel [4], Ursula Peintner [5] and Bianka Siewert [1,*]

Abstract: Fungi have developed a wide array of defense strategies to overcome mechanical injuries and pathogen infections. Recently, photoactivity has been discovered by showing that pigments isolated from *Cortinarius uliginosus* produce singlet oxygen under irradiation. To test if this phenomenon is limited to dermocyboid Cortinarii, six colourful *Cortinarius* species belonging to different classical subgenera (i.e., *Dermocybe*, *Leprocybe*, *Myxacium*, *Phlegmacium*, and *Telamonia*) were investigated. Fungal extracts were explored by the combination of *in vitro* photobiological methods, UHPLC coupled to high-resolution tandem mass spectrometry (UHPLC-HRMS^2), feature-based molecular networking (FBMN), and metabolite dereplication techniques. The fungi *C. rubrophyllus* (*Dermocybe*) and *C. xanthophyllus* (*Phlegmacium*) exhibited promising photobiological activity in a low concentration range (1–7 µg/mL). Using UHPLC-HRMS^2-based metabolomic tools, the underlying photoactive principle was investigated. Several monomeric and dimeric anthraquinones were annotated as compounds responsible for the photoactivity. Furthermore, the results showed that light-induced activity is not restricted to a single subgenus, but rather is a trait of *Cortinarius* species of different phylogenetic lineages and is linked to the presence of fungal anthraquinones. This study highlights the genus *Cortinarius* as a promising source for novel photopharmaceuticals. Additionally, we showed that putative dereplication of natural photosensitizers can be done by FBMN.

Supplementary Information

Table of contents

1 Mycochemical part 1

1.1 Phylogenetic analysis 1

1.2 Fungal biomaterial 2

1.3 Extraction 2

1.4 DMA-assay: comparability of different light sources (green vs. blue) used for irradiation 3

2 (Photo)cytotoxicity evaluation 4

2.1 (Photo)cytotoxicity assay using a blue light source (468 nm) 4

2.1.1 EC_{50} values and selectivity indices 4

2.2 (Photo)cytotoxicity experiments using a green light source (519 nm) 4

2.2.1 Comparison of different light doses 4

2.2.2 EC_{50} values and selectivity indices 6

2.3 Micrographs of treated cells (48 hours after irradiation) 7

2.3.1 Blue light experiments 7

2.3.2 Green light experiments 10

3 Feature-based molecular networking (FBMN) 15

3.1 UPLC-HRMS analysis 15

3.2 MS data pretreatment 15

3.3 Molecular network generation and taxonomically informed metabolite annotation (ISDB-DNP-Taxo) 16

3.4 Characterization of the Feature-Based Molecular Network (FBMN) 16

3.4.1 Generation of the variable “VIS-Signal” 16

3.4.2 Identification of photoactive clusters 17

3.4.3 *In silico* annotation by Sirius 18

3.4.4 Annotation results 18

3.4.4.1 General remarks 18

3.4.5 Specificity of features 34

3.4.6 Annotation-hit-rate 36

3.4.7 ISDB-DNP-annotations originating from the family of Cortinariaceae 36

3.4.8 Chemical taxonomy (ClassyFire) representation 38

3.4.9 Identification of compound classes associated with photoactivity 40

3.4.10 Active clusters sorted by their polarity 40

3.4.11 Mass spectral data: Cluster D – Chlorinated anthraquinones 42

4 Literature 46

1 Mycochemical part

1.1 Phylogenetic analysis

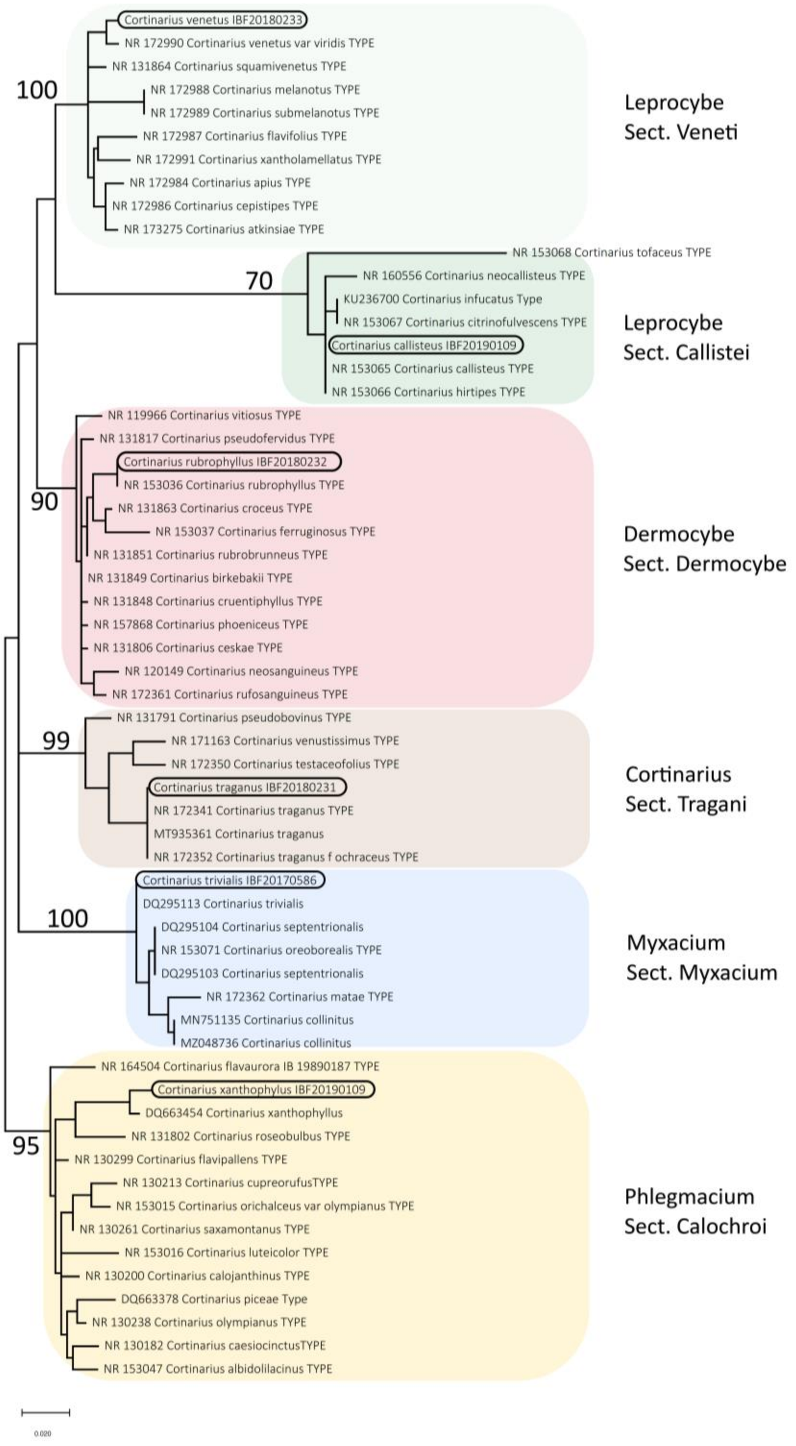

Figure S1. Phylogenetic placement of the six investigated *Cortinarius* species (highlighted) into six lineages representing sections in subgenera of *Cortinarius.* The phylogenetic analysis is based on 60 rDNA ITS sequences. Maximum parsimony – based bootstrap support is provided besides the respective lineages.

1.2 Fungal biomaterial

Table S1. *Cortinarius* collections used in this study with respective voucher numbers and collection data. Vouchers are deposited in IBF.

Cortinarius	Authority	Subgenus / Section	Voucher/ GenBank	Collection date	Origin
C. traganus	(Fr.) Fr.	Cortinarius / Sect. Tragani	IBF20180231/ MW880290	15.10.2018	Italy, Toscana, Pistoia, Abetone
C. rubrophyllus	L. Gray	Dermocybe / Sect. Dermocybe	IBF20180232/ MW880273	15.10.2018	Italy, Toscana, Pistoia, Abetone
C. venetus	(Fr.) Fr.	Leprocybe / Sect. Veneti	IBF20180233/ MW880292	15.10.2018	Italy, Toscana, Pistoia, Abetone
C. callisteus	Fr.	Leprocybe / Sect. Veneti	IBF20190145/ MW871552	19.10.2019	Italy, Emilia-Romagna, Parma, Bedonia
C. xanthophyllus	(Cooke) Rob. Henry	Phlegmacium / Sect. Calochroi Subsect. Rufoolivacei	IBF20190109/ MW898453	19.10.2019	Italy, Emilia-Romagna, Parma, Borgotaro
C. trivialis	J.E. Lange	Myxacium / Sect. Myxacium	IBF20170586/ MW880291	17.10.2017	Italy, Massa Carrara Passo, Parma, Bedonia

1.3 Extraction

Table S2. Yields of the extracts prepared by repeated ultra-sonification with acidified acetone.

	Mass biomaterial [mg]	Solvent	Extract yield [mg (dw)]
C. traganus	1709.7	Acidified acetone	18.2 (1.1%)
C. rubrophyllus	1099.2		35.3 (3.2%)
C. venetus	611.1		17.5 (2.9%)
C. callisteus	1643.7		14.6 (0.9%)
C. xanthophyllus	1051.8		26.6 (2.5%)
C. trivialis	1788.1		22.0 (1.1%)

1.4 DMA-assay: comparability of different light sources (green vs. blue) used for irradiation

Since the correction factor used for calculating the singlet oxygen yield is based on the probability of absorption [1], the fungal extracts´ optical densities at 468 and 519 nm need to be looked at to get a grasp on the comparability of results. Figure S2 shows that both active extracts (i.e. *C. rubrophyllus* and *C. xanthophyllus*) have similar absorption probabilities and thus similar correction factors. Therefore, the results from the green light irradiation group are not directly, but rather indirectly comparable to results from experiments which employed a blue light source, when the singlet oxygen quantum yields of the respective reference compounds are kept in mind.

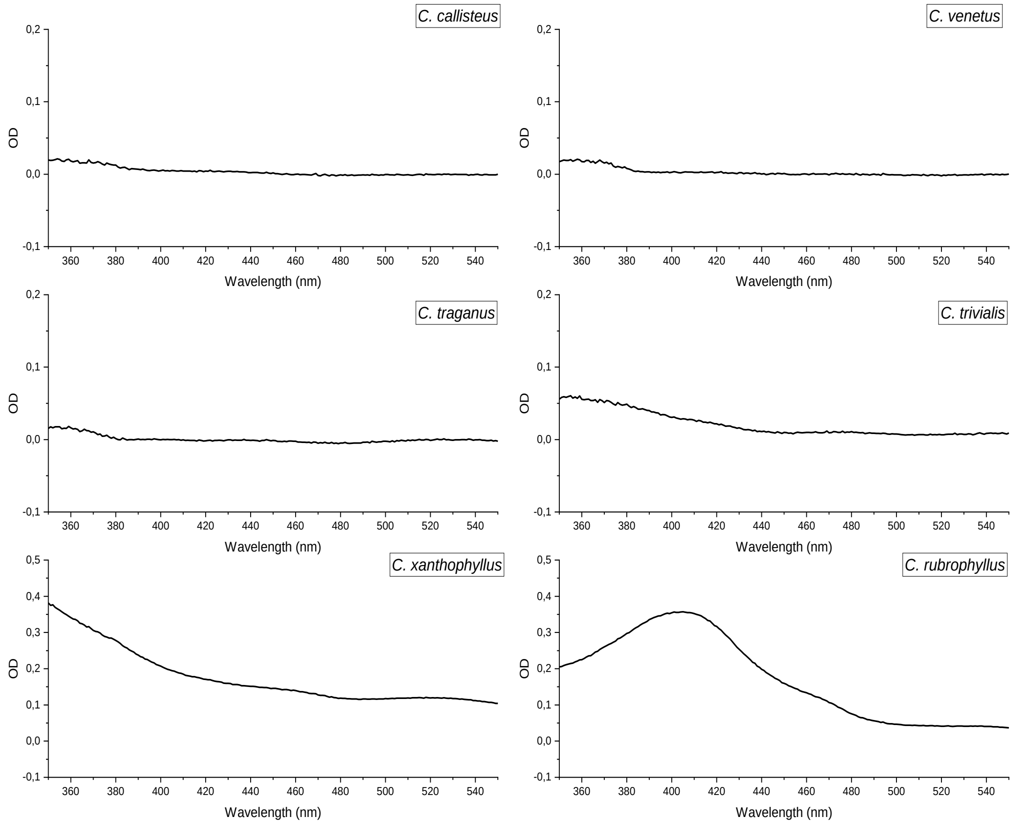

Figure S2. UV/Vis-spectra of the fungal extracts (c = 1 mg/mL) in ethanol.

2 (Photo)cytotoxicity evaluation

2.1 (Photo)cytotoxicity assay using a blue light source (468 nm)

2.1.1 EC_{50} values and selectivity indices

The results of all (photo)cytotoxicity experiments are listed in the tables below (Table S3, Table S4). EC_{50} values including their 95% confidence intervals were calculated with GraphPad Prism 5 employing the "log(inhibitor) vs. normalized response"-equation (= relative Hill-Slope equation).

Table S3. The results of the (photo)cytotoxicity screening of the acetone extracts of *Cortinarius callisteus*, *C. venetus*, *C. traganus*, *C. trivialis*, *C. xanthophyllus*, and *C. rubrophyllus*. The extracts' dark cytotoxicity as well as the amplification of their cytotoxic behavior via irradiation with a blue light source (λ = 468 ± 27 nm. 9.3 J/cm²) against the three cancer cell lines A549, AGS, and T24 was evaluated. EC_{50} values in combination with their 95% confidence intervals are given in µg/mL. The ratio of cells killed in the dark versus cells killed under irradiation (i.e. selectivity index) is depicted as well.

EC_{50} [µg/mL]	A549 (BL, 468 nm)	A549 (D)	S.I. A549	AGS (BL, 468 nm)	AGS (D)	S.I. AGS	T24 (BL, 468 nm)	T24 (D)	S.I. T24
C. callisteus^{+}	>20	>20		>20	>20		>20	>20	
C. venetus^{+}	>20	>20		13.0 (17.4; 7.5)	5.2 (4.3; 2.4)		>20	>20	
C. traganus^{+}	15.4 (11.4; 6.6)	>20		12.4 (22.9; 8.0)	5.4 (2.4; 1.6)		>20	>20	
C. trivialis^{+}	>20	>20		12.2 (25.1; 8.2)	5.1 (6.5; 2.8)		>20	>20	
C. xanthophyllus^{++}	3.7 (5.3; 2.2)	>20	>10.2	4.6 (4.5; 2.3)	>20	>8.1	1.5 (1.4; 0.7)	>20	>25.3
C. rubrophyllus^{++}	11.1 (6.8; 4.2)	>20	>1.8	10.1 (6.3; 3.9)	>20	>2.0	6.1 (2.1; 1.5)	>20	>3.3
B. ilicifolia***	17	>50							

*data adapted from [1]; $^{+}$ measured as biological duplicate; $^{++}$ measured as triplicate.

2.2 (Photo)cytotoxicity experiments using a green light source (519 nm)

2.2.1 Comparison of different light doses

In order to decide on an appropriate light dose for our photocytotoxicity investigations employing green light (λ = 519 ± 33 nm), we tested following irradiation durations: 7 minutes (= 9.4 J/cm^2) and 15 minutes (= 20.1 J/cm^2). Hence, the light-dependent cytotoxicity of the *Cortinarius xanthophyllus* acetone extract was tested against cells of three cancer cell lines (i.e., AGS, T24, and A549) as well as against cells of the murine fibroblast cell line NIH3T3. Figure S3 shows that by using the higher light

dose (= 20.1 J/cm^2) the dose-response curve is clearly shifted towards lower EC_{50}-values (i.e. left shift). Therefore, an irradiation duration of 15 minutes was chosen for our final green light experiments. Green light in the absence of a fungal extract showed no signs of photocytotoxicity.

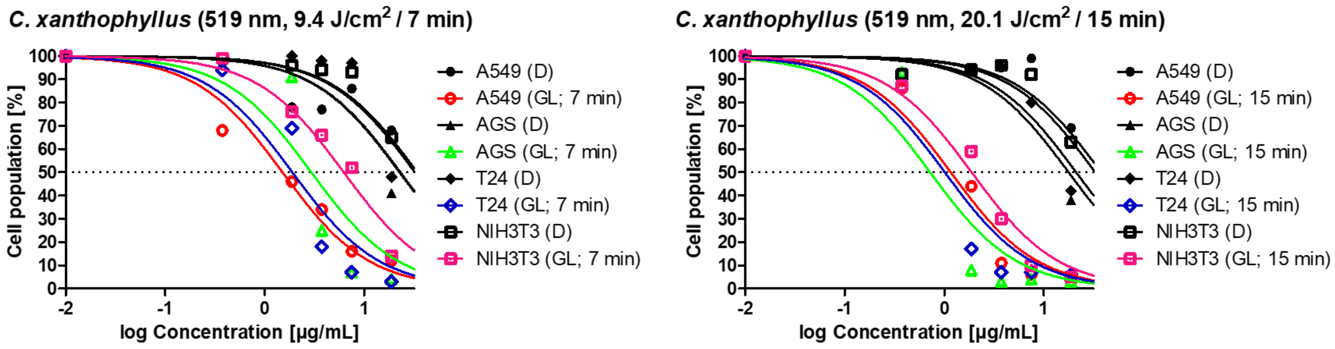

Figure S3. Investigation of the green light-dependent cytotoxicity of the *C. xanthophyllus* acetone extract under two different irradiation durations (= light doses: 9.4 versus 20.1 J/cm^2) against the cell lines A549, AGS, T24, and NIH3T3. Applying the higher light dose resulted in a clear shift in the dose-response curve towards the left side (i.e. lower EC_{50} values). D: dark, GL: green light.

2.2.2 EC_{50} values and selectivity indices

Table S4. Results of the (photo)cytotoxicity assay of the acetone extracts of *C. xanthophyllus* and *C. rubrophyllus* employing green light (λ = 519 ± 33 nm, 20.1 J/cm^2). Rose bengal was tested for validation and comparative purposes. EC_{50} values [µg/mL] including their 95% confidence intervals as well as the selectivity indices are depicted.

EC_{50} [Extracts: µg/mL; Rose Bengal: µM]	A549 (GL, 519 nm)		A549 (D)	S.I. A549	AGS (GL, 519 nm)		AGS (D)	S.I. AGS	T24 (GL, 519 nm)		T24 (D)	S.I. T24	NIH3T3 (GL, 519 nm)		NIH3T3 (D)		S.I. NIH3T3
C. xanthophyllus	1.6	0.4<br>0.3	> 37.5	> 23.4	0.8	0.5<br>0.3	> 37.5	> 46.9	1.2	0.6<br>0.4	> 37.5	> 32.3	2.1	0.6<br>0.5	> 37.5		> 17.9
C. rubrophyllus	>37.5		> 37.5	-	> 37.5		> 37.5	-	> 37.5		> 37.5	-	5.5	5.2<br>2.7	> 37.5	0.0<br>0.0	> 6.8
**Rose bengal**	2.7	0.8<br>0.6	> 6.25	> 2.3	4.0E-2	3.9E-2<br>2.0E-2	> 6.25	> 155.9	0.1	0.1<br>0.0	> 6.25	> 58.1	0.1	0.1<br>0.0	> 6.25		> 73.4

2.3 Micrographs of treated cells (48 hours after irradiation)

2.3.1 Blue light experiments

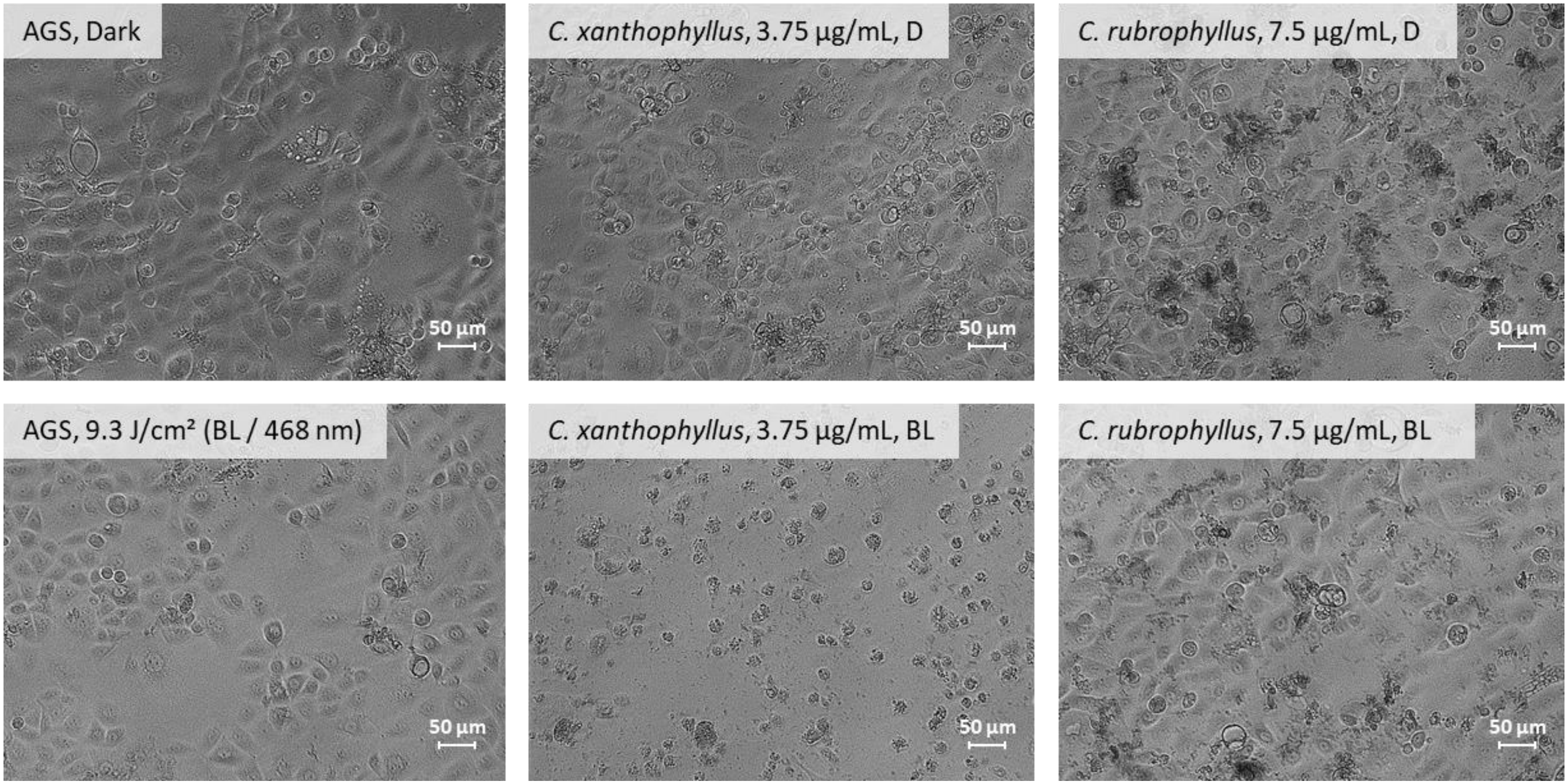

Figure S4. Micrographs of cells of the stomach cancer cell line (AGS / human Caucasian gastric adenocarcinoma, 200x magnification) treated (24 h) with the acetone extracts of *C. xanthophyllus* (3.75 µg/mL) and *C. rubrophyllus* (7.5 µg/mL). The upper line of pictures shows treated cells in the dark, the lower after irradiation with blue light (468 nm, 9.3 J/cm²).

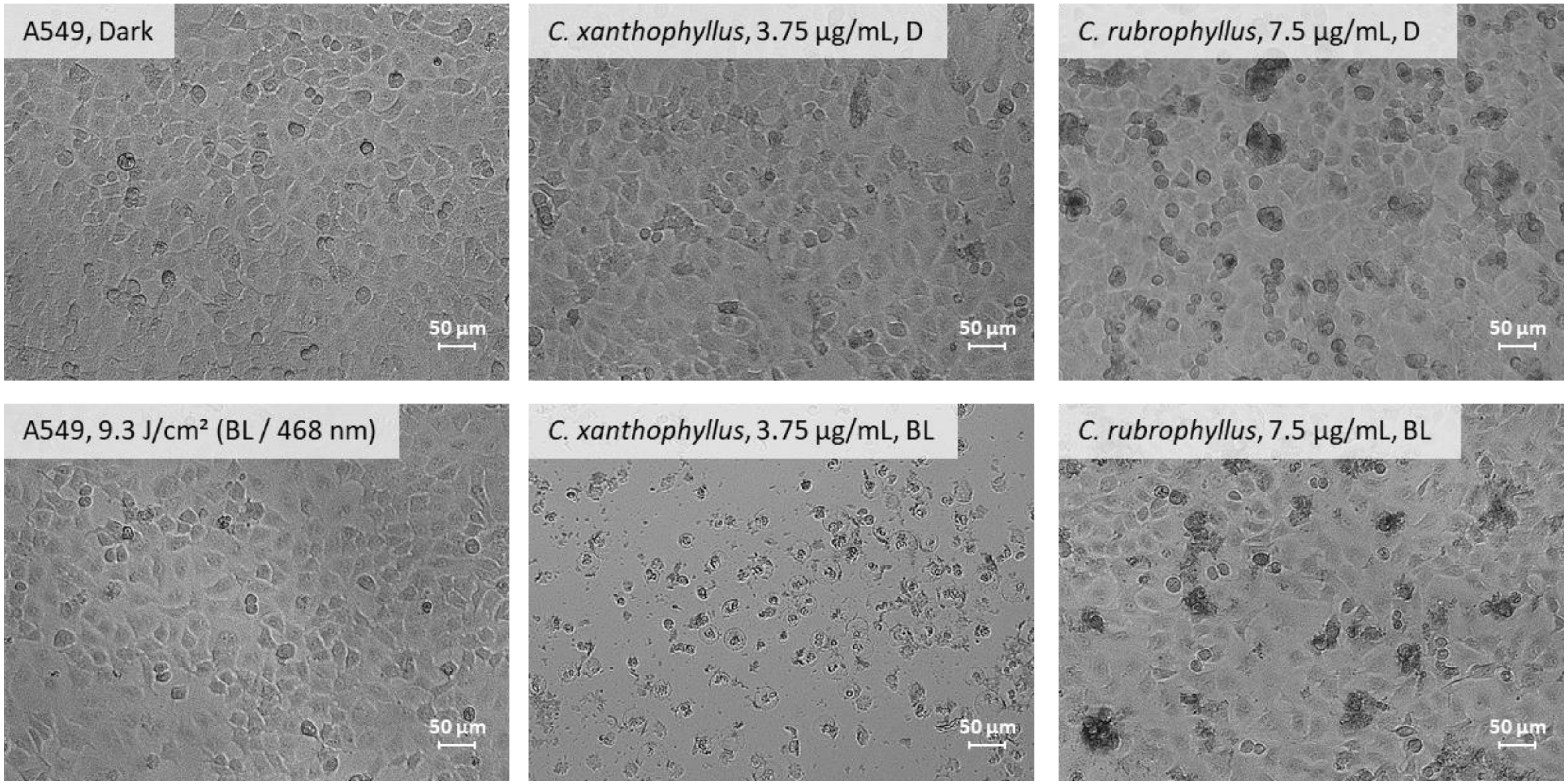

Figure S5. Micrographs of cells of the lung cancer cell line (A549 / human Caucasian lung carcinoma, 200x magnification) treated (24 h) with the acetone extracts of *C. xanthophyllus* (3.75 µg/mL) and *C. rubrophyllus* (7.5 µg/mL). The upper line of pictures shows treated cells in the dark, the lower after irradiation with blue light (468 nm, 9.3 J/cm²).

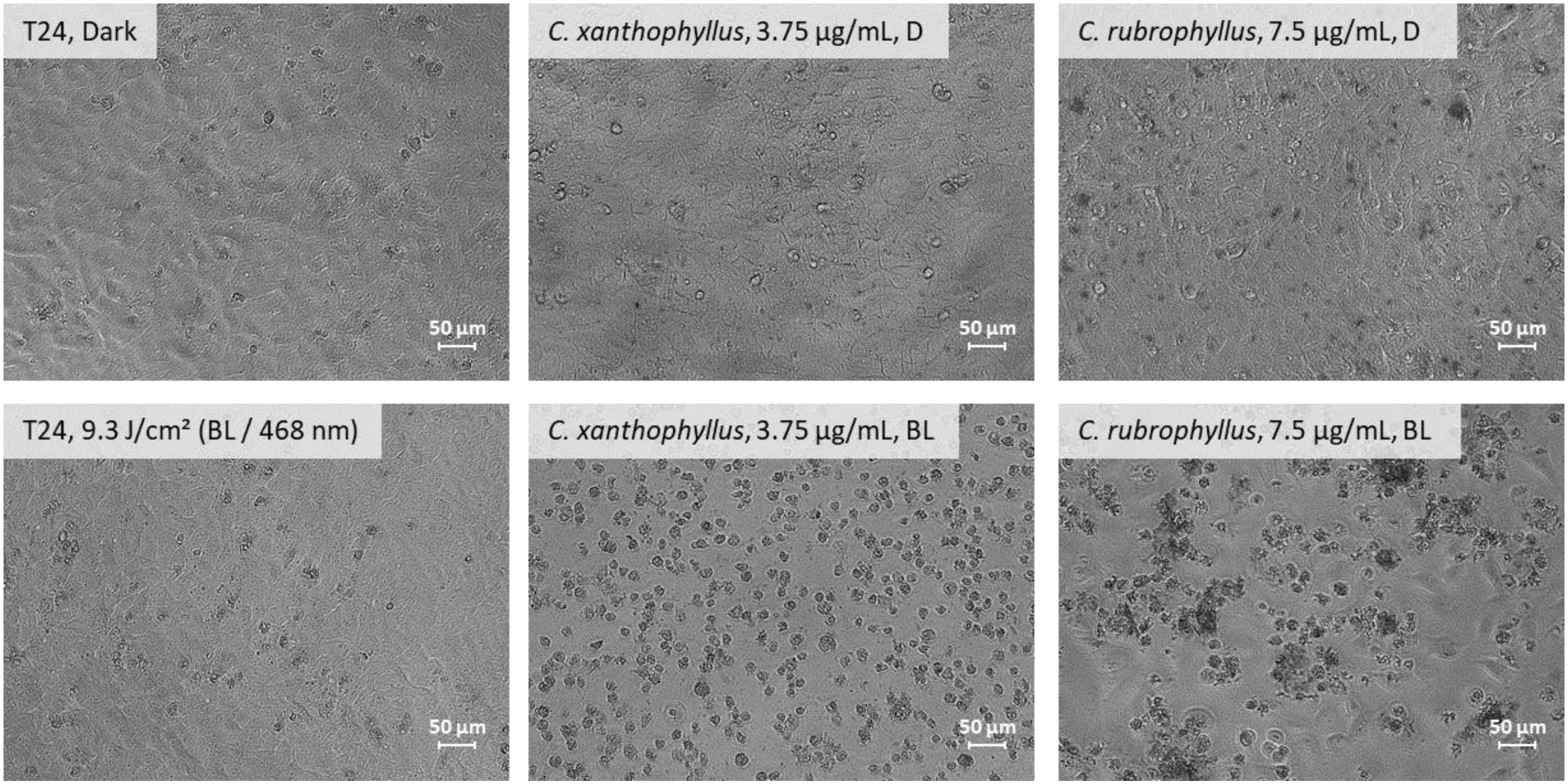

Figure S6. Micrographs of cells of the bladder cancer cell line (T24 / human bladder carcinoma, 200x magnification) treated (24 h) with the acetone extracts of *C. xanthophyllus* (3.75 µg/mL) and *C. rubrophyllus* (7.5 µg/mL). The upper line of pictures shows treated cells in the dark, the lower after irradiation with blue light (468 nm, 9.3 J/cm²).

2.3.2 Green light experiments

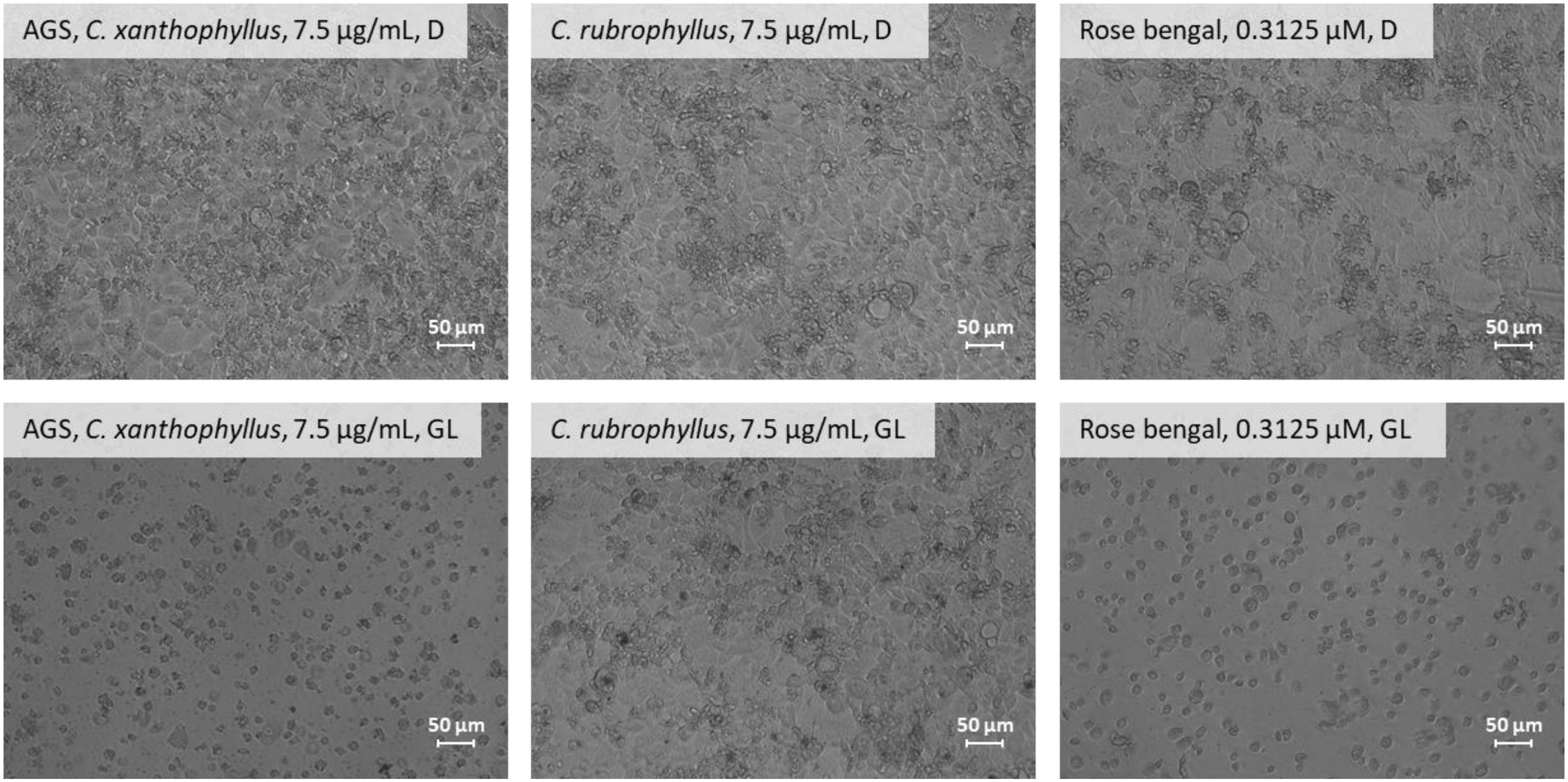

Figure S7. Micrographs of cells of the stomach cancer cell line (AGS / human Caucasian gastric adenocarcinoma, 200x magnification) treated (24 h) with the acetone extracts of *C. xanthophyllus* (7.5 µg/mL), *C. rubrophyllus* (7.5 µg/mL), and a DMSO solution of rose bengal (0.3125 µM). The upper line of pictures shows treated cells in the dark, the lower after irradiation with green light (519 nm, 20.1 J/cm²).

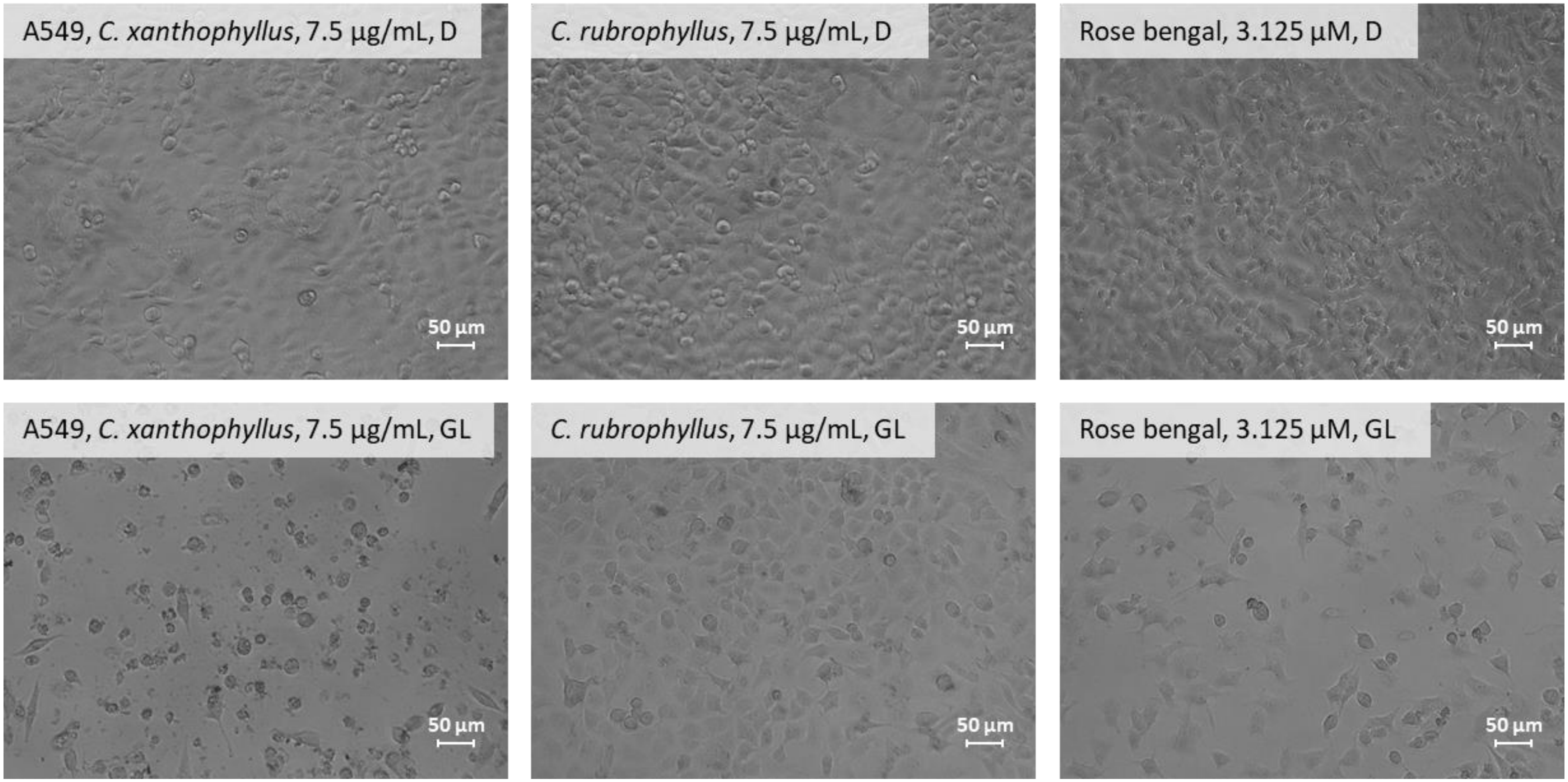

Figure S8. Micrographs of cells of the lung cancer cell line (A549 / human Caucasian lung carcinoma, 200x magnification) treated (24 h) with the acetone extracts of *C. xanthophyllus* (7.5 µg/mL), *C. rubrophyllus* (7.5 µg/mL), and a DMSO solution of rose bengal (3.125 µM). The upper line of pictures shows treated cells in the dark, the lower after irradiation with green light (519 nm, 20.1 J/cm²).

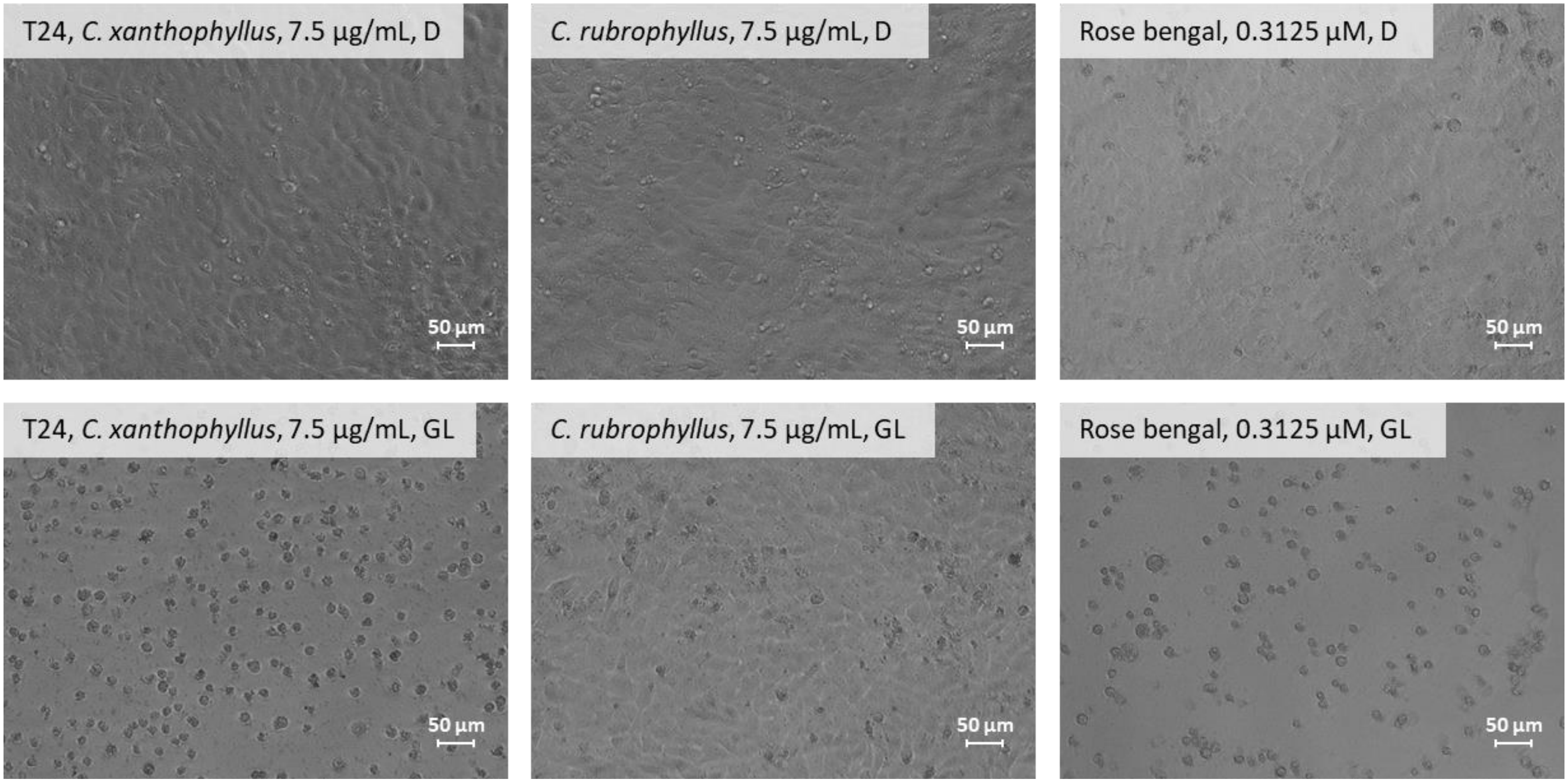

Figure S9. Micrographs of cells of the bladder cancer cell line (T24 / human bladder carcinoma, 200x magnification) treated (24 h) with the acetone extracts of *C. xanthophyllus* (7.5 µg/mL), *C. rubrophyllus* (7.5 µg/mL), and a DMSO solution of rose bengal (0.3125 µM). The upper line of pictures shows treated cells in the dark, the lower after irradiation with green light (519 nm, 20.1 J/cm²).

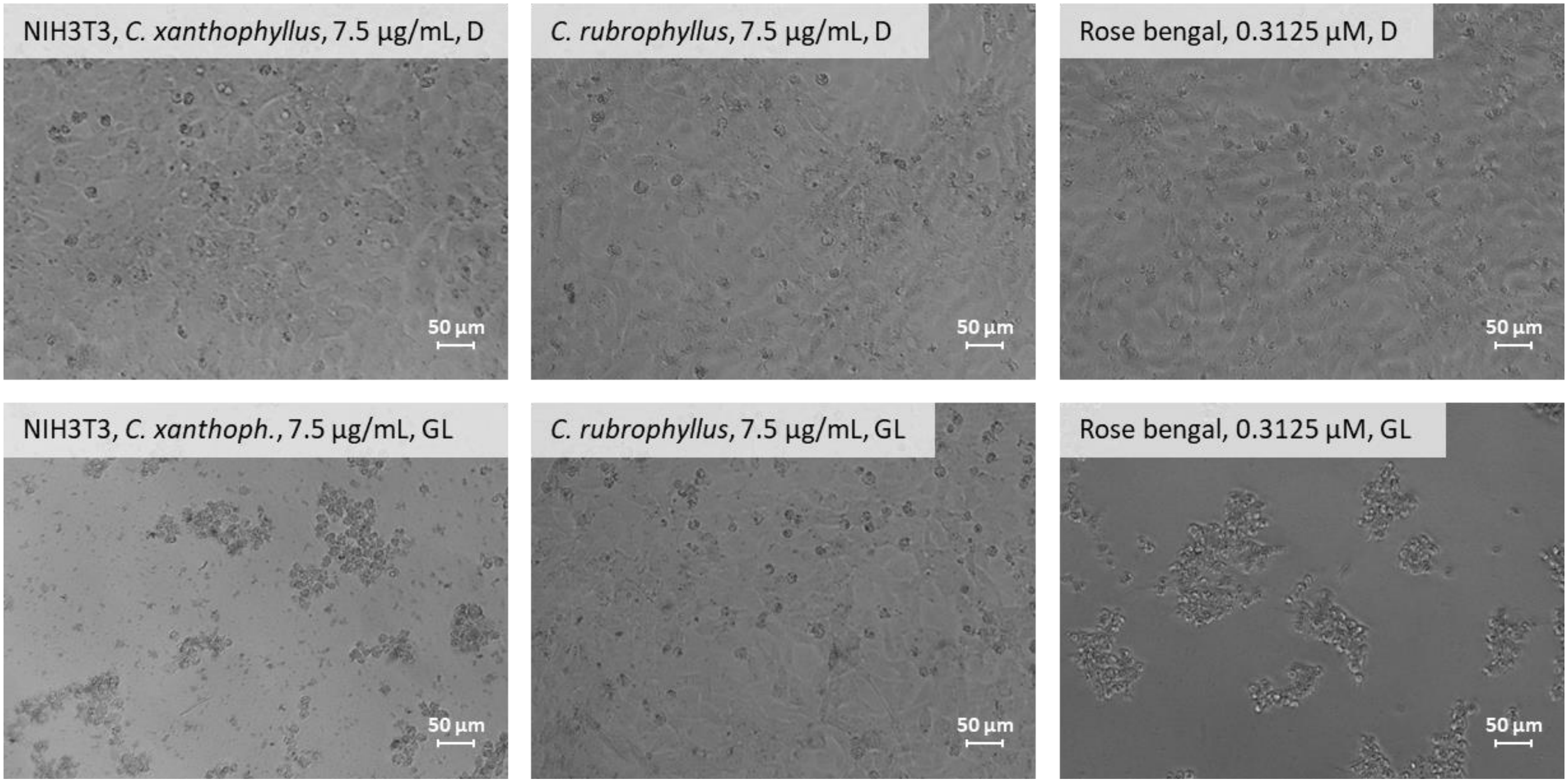

Figure S10. Micrographs of cells of the mouse embryonic fibroblast cell line (NIH3T3 / murine fibroblasts, 200x magnification) treated (24 h) with the acetone extracts of *C. xanthophyllus* (7.5 µg/mL), *C. rubrophyllus* (7.5 µg/mL), and a DMSO solution of rose bengal (0.3125 µM). The upper line of pictures shows treated cells in the dark, the lower after irradiation with green light (519 nm, 20.1 J/cm²).

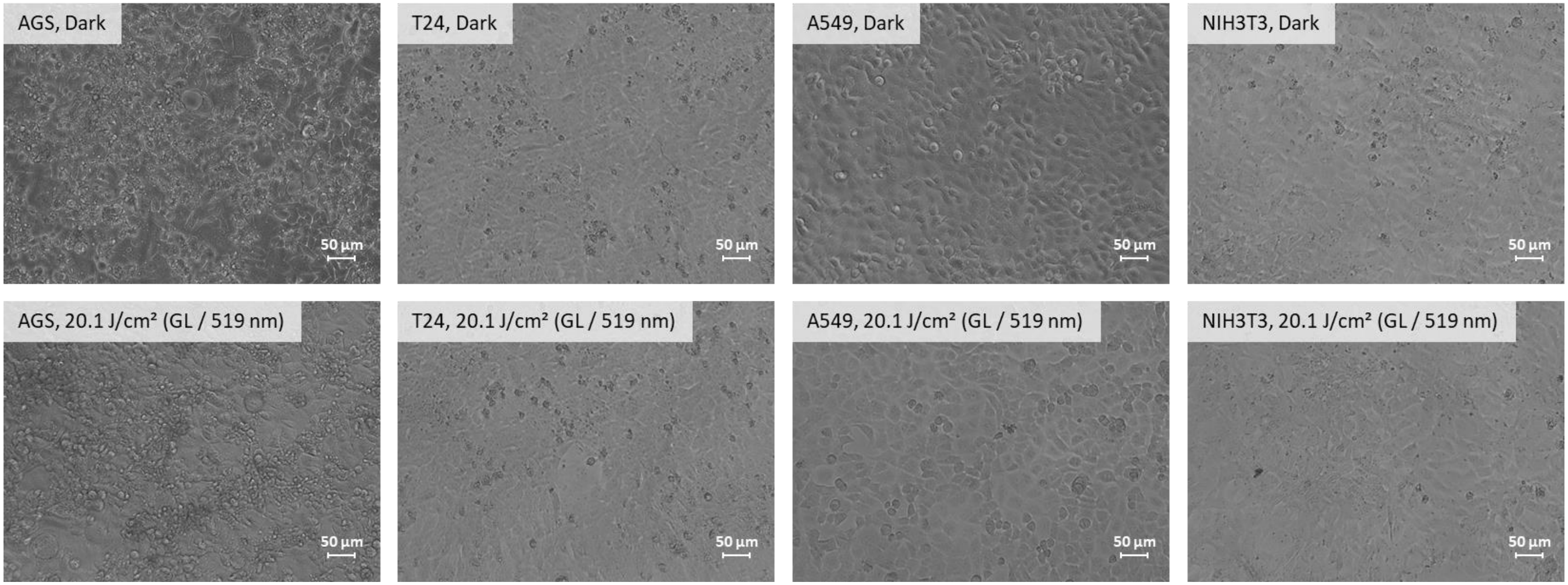

Figure S11. Micrographs of all cell lines used in this study (AGS, T24, A549, and NIH3T3, 200x magnification) after treatment (24 h) with the solvent control (i.e., medium only). The upper line of pictures shows treated cells in the dark, the lower after irradiation with green light (519 nm, 20.1 J/cm²).

3 Feature-based molecular networking (FBMN)

3.1 UPLC-HRMS analysis

Liquid-chromatographic profiling of the fungal extracts (chapter 1.3) and two enriched fractions of a methanolic *C. uliginosus* extract [2] was done on a Waters Acquity UPLC system hyphenated to a Q-Exactive Focus mass spectrometer (Thermo Scientific, Bremen, Germany), using a heated electrospray ionization (HESI-II) source and a CAD detector (Thermo Scientific, Bremen, Germany). The instrument was controlled using Thermo Scientific Xcalibur 3.1 software. A Waters Acquity BEH C18 50 x 2.1 mm, 1.7 µm column was used as stationary phase, water with 0.1 % formic acid (A) and acetonitrile with 0.1 % formic acid (B) were chosen as mobile phases. The analysis was performed using a linear gradient of 5-100 % B over 7 min and an isocratic step at 100 % B for 1 min. Flow rate and injection volume were set to 600 µL/min and 2 µl, respectively. ESI parameters were as follows: source voltage, 3.5 kV (neg); sheath gas flow rate (N_2), 55 units; auxiliary gas flow rate, 15 untis; spare gas flow rate, 3.0; capillary temperature, 350.00 °C, S-Lens RF Level, 45. The mass analyzer was calibrated using a mixture of caffeine, methionine-arginine-phenylalanine-alanine-acetate (MRFA), sodium dodecyl sulfate, sodium taurocholate, an Ultramark 1621 in an acetonitrile/methanol/water solution containing 1 % formic acid by direct injection. The data-dependent MS/MS events were performed on the three most intense ions detected in full scan MS. The MS/MS isolation window width was 1 Da, and the stepped normalized collision energy (NCE) was set to 15, 30, and 45 units. In data-dependent MS/MS experiments, full scans were acquired at a resolution of 35 000 FWHM (at m/z 200) and MS/MS scans at 17 500 FWHM both with an automatically determined maximum injection time. After being acquired in a MS/MS scan, parent ions were placed in a dynamic exclusion list for 2.0 s.

3.2 MS data pretreatment

The data was converted from .RAW (Thermo) format to .mzXML with MSConvert software [3]. The converted files were treated using the MZmine 2 software (v2.53) [4]. The parameters were set as follows (negative mode): the mass detection was done using the centroid mass detector with minimum noise level of 1.5E5 for MS level 1, and 0 for MS level 2. Chromatograms were built using the ADAP algorithm with a minimum group size of scans of 5, minimum group intensity threshold of 1.5E5, minimum highest intensity of 1.5E5 and m/z tolerance of 8.0 ppm. Chromatogram deconvolution was achieved using the wavelets (ADAP) algorithm. The intensity window S/N was used as S/N estimator and the S/N threshold was set to 80. The minimum feature height was set to 1.5E5, the coefficient area threshold was 100, the peak duration range used was from 0.01 to 0.80 min and the RT wavelet range from 0.01 to 0.04 min. Isotopes were detected using the isotopes peaks grouper with a m/z tolerance of 8.0 ppm, a RT tolerance of 0.05 min (absolute), the maximum charge set to 1 and the representative isotope used was the most intense. Peak alignment was done with the join aligner algorithm, m/z tolerance at 8 ppm, absolute RT tolerance 0.05 min, weight for m/z 70 Da and weight for RT 30. The peak list was gap-filled with the same RT and m/z range gap filler (m/z tolerance at 8.0 ppm). The resulting aligned peak list was filtered using the peak-list rows filter option in order to keep only features associated with MS2 scans. An adduct search (Na^+, K^+, NH_4^+, CH_3CN^+, CH_3OH^+, $C_3H_8O^+$ (IPA^+)) was performed with the RT tolerance set at 0.1 min and the maximum relative peak

height at 500%. Utilizing an in-house database of fungal pigments based on the work of Gill and Steglich [5,6], dereplication was done whereby a m/z tolerance of 8.0 ppm was applied.

3.3 Molecular network generation and taxonomically informed metabolite annotation (ISDB-DNP-Taxo)

A feature-based molecular network (FBMN) [7] was created utilizing the .mgf file exported from the MZmine data preprocessing. The spectral data was uploaded on the GNPS platform (https://gnps.ucsd.edu/) [8]. The precursor ion mass tolerance was set to 0.02 Da and the MS/MS fragment ion tolerance to 0.02 Da. The edges were filtered to have a cosine score above 0.6 and more than 4 matched peaks. The edges between two nodes were kept in the network if and only if each of the nodes appeared in each other's respective top 10 most similar nodes. The maximum size of a molecular family was set to 100, and the lowest scoring edges were removed from molecular families until the molecular family size was below this threshold. The spectra in the network were then searched against GNPS spectral libraries. All matches kept between network spectra and library spectra were required to have a cosine score above 0.7 and at least 6 matched peaks. The job is available in the following hyperlink: https://gnps.ucsd.edu/ProteoSAFe/status.jsp?task=ff9b52921d0a4867b81b7373de209a68. The output data from GNPS was used to annotate against the *in silico* database (ISDB-DNP) [9]. The resulting candidate annotations were re-ranked using the script for taxonomically informed metabolite annotation [10]. The script included the taxonomical information for the species studied. Visualization of acquired results was achieved with Cytoscape (v3.8.2) software [11].

3.4 Characterization of the Feature-Based Molecular Network (FBMN)

The FBMN based on the six investigated fungal extracts, two enriched fractions resulting from the mycochemical investigation of *Cortinarius uliginosus* [2], and an extraction blank comprises 3745 nodes and 4643 edges. Out of the 3745 nodes, 1920 nodes are self-loops. The 1825 non-self-loops were gathered into 461 different clusters. It was further characterized concerning following aspects: number of nodes, edges, and clusters, the specificity of features, the annotation-hit-rate using different spectral databases (GNPS vs. ISDB-DNP), ISDB-DNP-annotations based on compounds isolated from species belonging to the Cortinariaceae family, the compound classes representing the chemical space covering all extracts (chemical taxonomy done by ClassyFire [12]), and the polarity (based on retention time) of the clusters associated with photoactivity.

3.4.1 Generation of the variable "VIS-Signal"

For each extract investigated, the chromatogram (λ_{det} = 468 nm) was extracted from the DAD scan. Via Origin 2020, all peaks of each spectrum were integrated and thus the start and the endpoint of each peak defined. The threshold value was set at 0.5% of the largest peak area. Next, utilizing Excel 365, for each feature of the combined features list it was identified in which extract this specific feature was most abundant. Thereafter, the features' retention time was correlated to the retention time range in which the peaks of that specific extract occurred. The dead volume between DAD and MS detector was negligible. When it was possible to assign the feature to a peak, the value was set to "1". In case it was not assigned, the value of this variable was set to "0". The result was exported as .csv and later imported into Cytoscape.

3.4.2 Identification of photoactive clusters

Photoactive molecular clusters were identified by combining the extract specificity-information (chapter 3.4.5) and the “VIS-Signal”-variable (chapter 3.4.1). An informational layer was created where features belonging to the *C. rubrophyllus* extract were highlighted in red and *C. xanthophyllus* features were presented in purple color (mapping of the respective “Peak area” as Image/Chart 1). The node size was coupled to the “VIS-Signal” information, meaning features capable of absorbing blue light (λ = 468 nm) were bigger in size than features lacking this ability (“VIS-Signal”: “Yes” = node size 70 – “No” = node size 20). Ten putatively photoactive clusters were identified and highlighted in the figure below (Figure S12). The clusters were provided with the identifiers A-J according to their appearance in the FBMN (left → right, top → bottom).

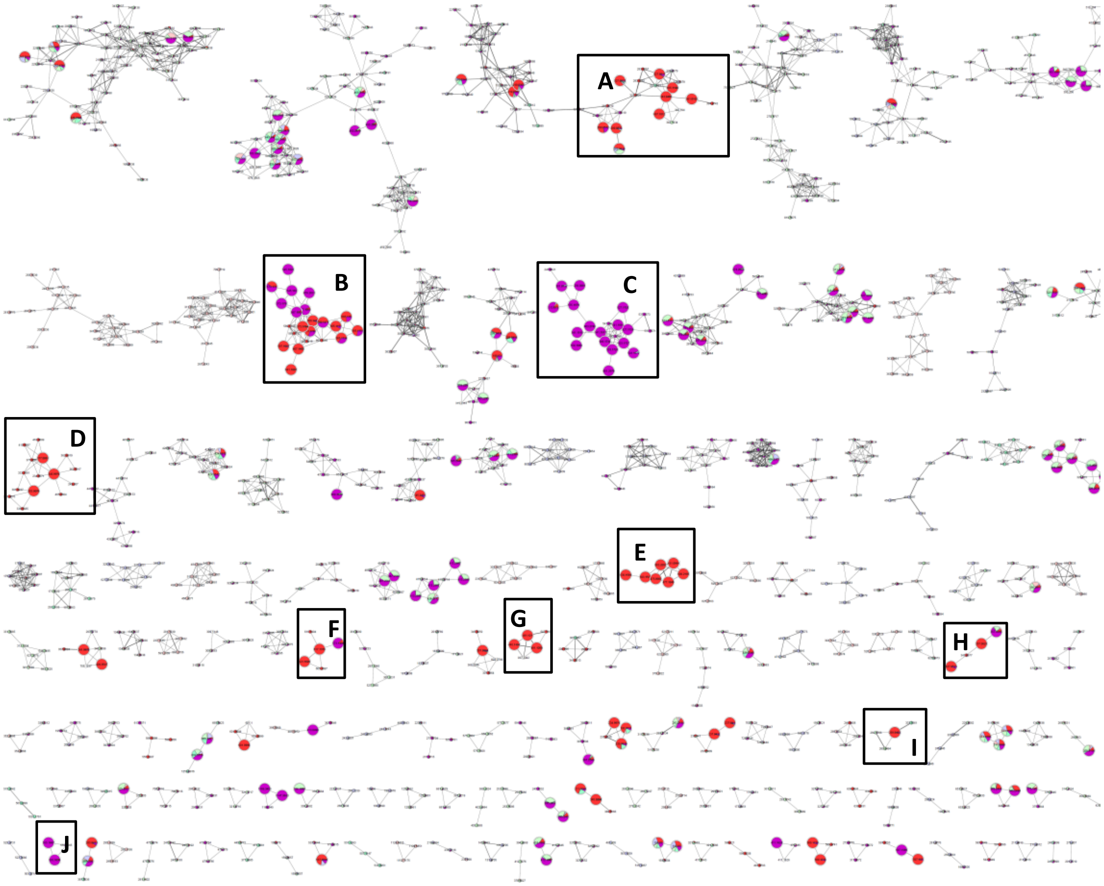

Figure S12. The FBMN embedded with an informational layer comprising the features´ abundance in both photoactive extracts (i.e. *C. rubrophyllus* = red, *C. xanthophyllus* = purple). Features showing an absorption in the visible range (λ = 468 nm) are drawn as big dots (“VIS-Signal” present = node size

70, “VIS-Signal” absent = node size 20), whereas features missing this quality are smaller in size. Ten putatively active clusters are highlighted with boxes including an alphabetic letter (A-J) as identifier.

3.4.3 *In silico* annotation by Sirius

The *in silico* annotation tool Sirius 4.4.29 [13] was employed to annotate the features present in the photoactive clusters. The search algorithm is based on molecular formula calculation and the prediction of a molecular fingerprint of a query compound from its fragmentation tree. Following parameters were used for the molecular formula calculation: Possible ionizations – $[M+H]^-$, $[M+Cl]^-$, $[M+Br]^-$; Instrument – Orbitrap, MS2 MassDev (ppm) – 5 ppm, molecular formula candidates: 10; Consider only formulas in DBs: All included DBs. The CSI:FingerID algorithm [14] was carried out with subsequent parameters: Search in DBs – All included DBs, Fallback Adducts: $[M-H]^-$, $[M+Cl]^-$, and $[M+Br]^-$. Sirius 4.4.29 is available at the following address: https://bio.informatik.uni-jena.de/software/sirius/.

3.4.4 Annotation results

3.4.4.1 General remarks

The annotation results for the active clusters are depicted in the following chapters (3.4.4.1.1-3.4.4.1.10). FBMN figures, visualized with Cytoscape [11], combined with tables showing relevant information generated via spectral annotation processes (In-house library, GNPS, ISDB-DNP-Taxo, and Sirius) are presented. The FBMN figures were embedded with an informational layer comprising the “Peak area”-variable of the fungal extracts as pie chart (Image/Chart 1; color code presented in Figure S13), the “VIS-Signal”-variable (chapter 3.4.1; “Yes” = node size 70 – “No” = node size 20), as well as the “originating from Cortinariaceae”-variable (chapter 3.4.7; “Yes” = octagonal shape; Figure S13). To give an overview of the confidence level achieved in the feature annotation, the four levels of accuracy reported in the Metabolomics Standard Initiative were adopted (i.e. “Identification level”: Table S5-Table S14) [15].

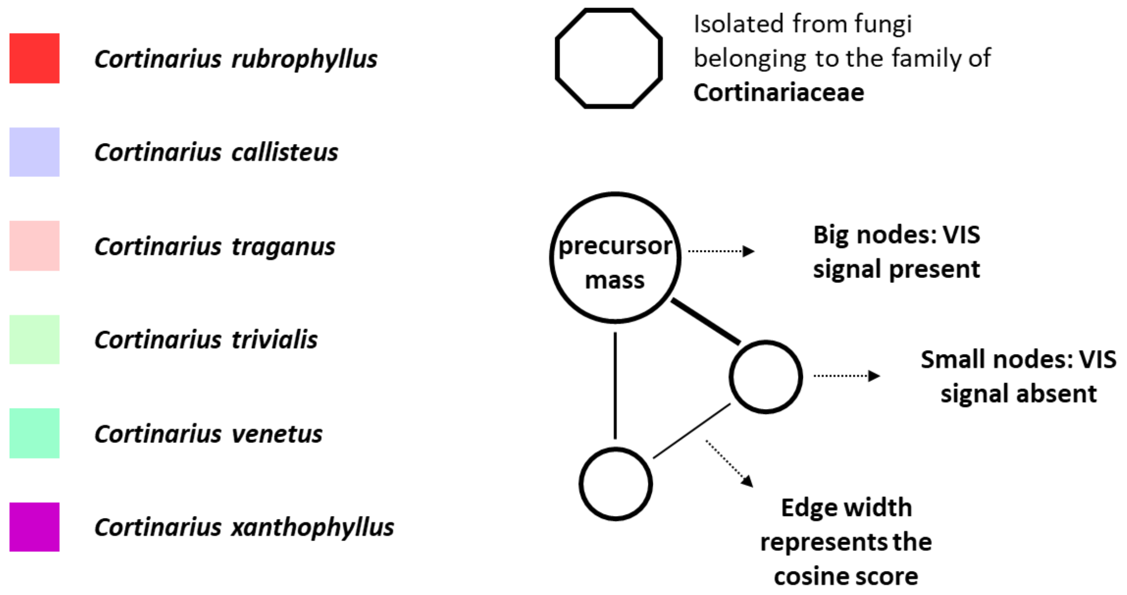

Figure S13. Color code used for visual representation of the "Peak area"-variable as pie chart in the figures below (Figure S14-Figure S23) as well as general remarks concerning the FBMN.

3.4.4.1.1 Cluster A – Monomeric anthraquinones

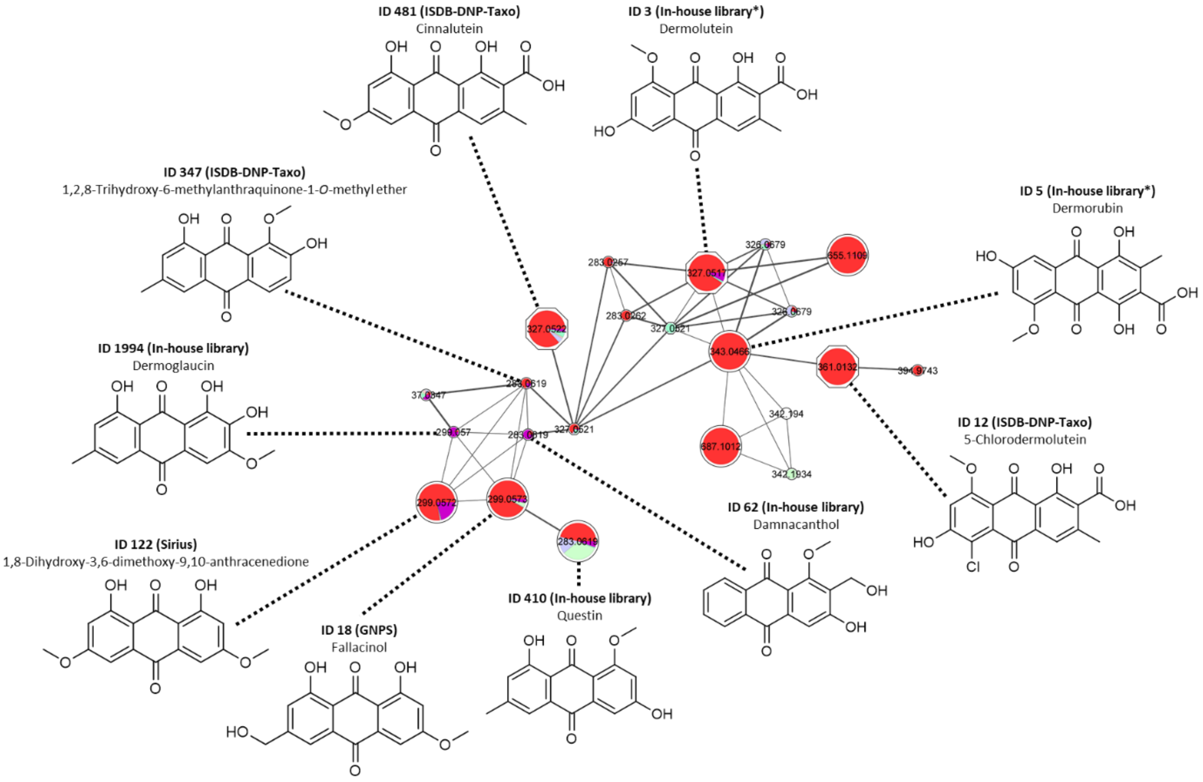

Figure S14. Annotated Cluster A. Each node displays the precursor mass (please zoom in). Feature annotations belonging to the family of Cortinariaceae are drawn as octagons. Out of the different molecule annotations listed in the table below (Table S5), the chemical structure was depicted, which was deemed most probable. Isolated compounds are highlighted with an asterisk (*).

Table S5. The annotation results for Cluster A presented as the respective ID (shared name), the Row m/z, the Row RT in minutes, the SMILES (top → bottom: In-house library, GNPS, ISDB-DNP-Taxo, Sirius; Black color … SMILES belonging to the chemical structure drawn in the figure, Grey color … additional hits, Empty … no hit at all), and the name. Isolated compounds are highlighted in red color.

ID (shared name)	Row m/z	Row RT [min]	SMILES (In-house library | GNPS | ISDB-DNP-Taxo | Sirius) [Rank]	Name [Identification level]
**18**	299.0572	3.69		Fallacinol [2]
			O=C1C2=C(O)C=C(OC)C=C2C(C3=C1C(O)=CC(CO)=C3)=O	
			COC1=C(O)C2=C(C(=O)C3=CC=CC=C3C2=O)C(OC)=C1O [2]	
			COC1=CC(=C2C(=C1)C(=O)C3=CC(=CC(=C3C2=O)O)OC)O [2]	
**3**	327.0517	2.39	O=C1C2=C(C=C(C)C(C(O)=O)=C2O)C(C3=CC(O)=CC(OC)=C31)=O	Dermolutein (In-house library) [1]
			COC1=CC2=C(C(O)=C1)C(=O)C1=C(C=C(C)C(C(O)=O)=C1O)C2=O [1]	
			CC1=C2C(=CC(=C1C(=O)O)O)C(=O)C3=CC(=CC(=C3C2=O)O)OC [1]	
**410**	283.0619	2.39	O=C1C2=C(OC)C=C(O)C=C2C(C3=C1C(O)=CC(C)=C3)=O	Questin (In-house library) [2]
			COC1=CC(O)=CC2=C1C(=O)C1=C(C=C(C)C=C1O)C2=O [1]	
			CC1=CC(=C2C(=C1)C(=O)C3=C(C=C(C=C3C2=O)OC)O)O [1]	
**122**	299.0571	3.63		1,8-Dihydroxy-3,6-dimethoxy-9,10-anthracenedione (Sirius) [2]
			O=C1C2=C(O)C=C(OC)C=C2C(C3=C1C(O)=CC(CO)=C3)=O	
			CC1=CC(O)=C2C(=O)C3=C(O)C(C)=CC(O)=C3C(=O)C2=C1O [2]	
			COC1=CC(=C2C(=C1)C(=O)C3=CC(=CC(=C3C2=O)O)OC)O [1]	
**1994**	299.0569	3.52	O=C1C2=C(C=C(C)C=C2O)C(C3=CC(OC)=C(O)C(O)=C31)=O	Dermoglaucin (In-house library) [2]
			COC1=CC(=CC2=C1C(=O)C3=C(C2=O)C=C(C=C3O)O)CO	
			COC1=C2OCOC2=C(OC)C2=C1OC1=CC=CC=C1C2=O [1]	
			COC1=CC(=C2C(=C1)C(=O)C3=CC(=CC(=C3C2=O)O)OC)O [1]	
**481**	327.0521	3.53		Cinnalutein (ISDB-DNP-Taxo) [2]
			COC1=CC2=C(C(O)=C1)C(=O)C1=C(C=C(C)C(C(O)=O)=C1O)C2=O [1]	
			COC1=CC=C(C=C1)N=NC2=C(C=CC(=C2)C(=O)OC)Cl [1]	
**5**	343.0465	2.54	O=C1C2=C(C(O)=C(C)C(C(O)=O)=C2O)C(C3=CC(O)=CC(OC)=C31)=O	Dermorubin (In-house library) [1]
			COC1=C(OC)C2=C3C(=C1)C(=O)OC1=C3C(=CC(O)=C1OC)C(=O)O2 [1]	
			CC1=C(C2=C(C(=C1C(=O)O)O)C(=O)C3=C(C=C(C=C3C2=O)OC)O)O [6]	
**12**	361.0131	2.60		5-Chlorodermolutein (ISDB-DNP-Taxo) [2]
			COC1=C2C(=O)C3=C(C=C(C)C(C(O)=O)=C3O)C(=O)C2=C(Cl)C(O)=C1 [1]	
			CC1=C(C(=C2C(=C1)C(=O)C3=C(C2=O)C(=CC(=C3Cl)O)OC)O)C(=O)O [1]	
**62**	283.0619	3.40	O=C1C2=C(C=C(O)C(CO)=C2OC)C(C3=CC=CC=C31)=O	Damnacanthol (In-house library) [2]
			COC1=CC(=CC2=C1C(=O)C(=CO2)C3=CC=C(C=C3)O)O	
			COC1=CC(O)=CC2=C1C(=O)C1=C(C=C(C)C=C1O)C2=O [1]	
			CC1=CC(=C2C(=C1)C(=O)C3=C(C=C(C=C3C2=O)OC)O)O [1]	
**347**	283.0618	3.22	O=C1C2=C(O)C=C(OC)C(O)=C2C(C3=C1C=CC(C)=C3)=O	1,2,8-Trihydroxy-6-methylanthraquinone-1-*O*-methyl ether (ISDB-DNP-Taxo) [2]
			COC1=CC(=CC2=C1C(=O)C(=CO2)C3=CC=C(C=C3)O)O	
			COC1=C(O)C=CC2=C1C(=O)C1=C(C=C(C)C=C1O)C2=O [1]	
			CC1=CC(=C2C(=C1)C(=O)C3=C(C=C(C=C3C2=O)OC)O)O [1]	

3.4.4.1.2 Cluster B – Preanthraquinones and dimeric anthraquinones

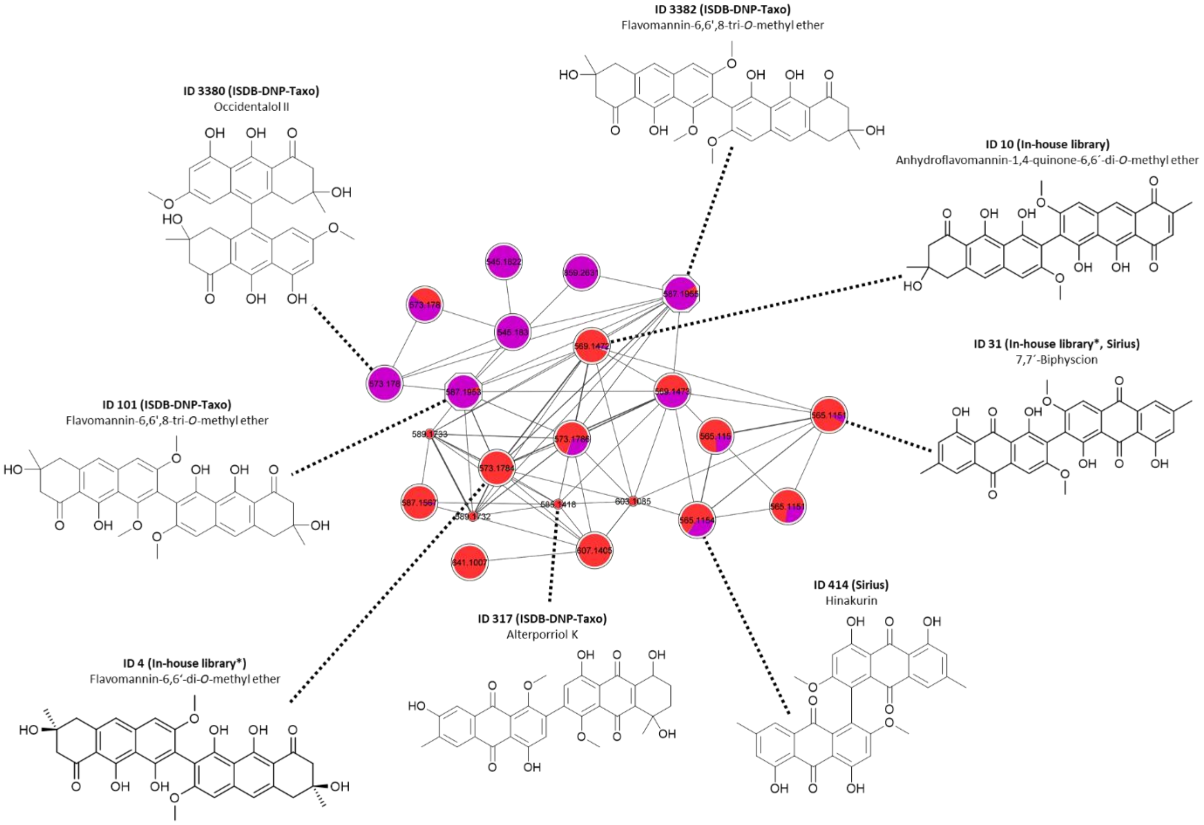

Figure S15. Annotated Cluster B. Each node displays the precursor mass (please zoom in). Feature annotations belonging to the family of Cortinariaceae are presented as octagons. Out of the different molecule annotations listed in the table below (Table S6), the chemical structure was depicted, which was deemed most probable. Isolated compounds are highlighted with an asterisk (*).

Table S6. The annotation results for Cluster B presented as the respective ID (shared name), the Row m/z, the Row RT in minutes, the SMILES (top → bottom: In-house library, GNPS, ISDB-DNP-Taxo, Sirius; Black color … SMILES belonging to the chemical structure drawn in the figure, Grey color … additional hits, Empty … no hit at all), and the name. Isolated compounds are highlighted in red color.

ID (shared name)	Row m/z	Row RT [min]	SMILES (In-house library | GNPS | ISDB-DNP-Taxo | Sirius) [Rank]	Name [Identification level]
**36**	589.1731	3.18		4-Hydroxy-flavomannin-6,6'-dimethyl ether A_1 (ISDB-DNP-Taxo, Sirius) [2]
			COC1=CC2=CC3=C(C(=O)CC(C)(O)C3)C(O)=C2C(O)=C1C1=C(O)C2=C(O)C3=C(C=C2C=C1OC)C(O)C(C)(O)CC3=O [1]	
			CC1(CC2=C(C(=O)C1)C(=C3C(=C2)C=C(C(=C3O)C4=C(C5=C(C6=C(C=C5C=C4OC)C(C(CC6=O)(C)O)O)O)O)OC)O)O [3]	
**31**	565.1151	6.10	O=C1C2=C(C(O)=C(C3=C(OC)C=C(C(C(C=C(C)C=C4O)=C4C5=O)=O)C5=C3O)C(OC)=C2)C(C6=C(O)C=C(C)C=C61)=O	7,7´-Biphyscion (In-house library, Sirius) [1]
			COC1=CC2=C(C(=O)C=C(O2)C2=CC=C(O)C=C2)C(O)=C1C1=C(O)C2=C(OC(=CC2=O)C2=CC=C(O)C=C2)C=C1OC [1]	
			CC1=CC(=C2C(=C1)C(=O)C3=CC(=C(C(=C3C2=O)O)C4=C(C=C5C(=C4O)C(=O)C6=C(C=C(C=C6C5=O)C)O)OC)OC)O [5]	
**414**	565.1153	6.23		Hinakurin (Sirius) [3]
			COC1=CC2=C(C(=O)C=C(O2)C2=CC=C(O)C=C2)C(O)=C1C1=C(O)C2=C(OC(=CC2=O)C2=CC=C(O)C=C2)C=C1OC [1]	
			CC1=CC(=C2C(=C1)C(=O)C3=C(C(=CC(=C3C2=O)O)OC)C4=C5C(=C(C=C4OC)O)C(=O)C6=C(C=C(C=C6C5=O)C)O)O [3]	
**38**	589.1733	3.20		4-Hydroxy-flavomannin-6,6'-dimethyl ether A_1 (ISDB-DNP-Taxo, Sirius) [2]
			COC1=CC2=CC3=C(C(=O)CC(C)(O)C3)C(O)=C2C(O)=C1C1=C(O)C2=C(O)C3=C(C=C2C=C1OC)C(O)C(C)(O)CC3=O [1]	
			CC1(CC2=C(C(=O)C1)C(=C3C(=C2)C=C(C(=C3O)C4=C(C5=C(C6=C(C=C5C=C4OC)C(C(CC6=O)(C)O)O)O)O)OC)O)O [3]	
**4**	573.1784	3.58	OC1=C(C2=C(OC)C=C(C=C(C[C@](C)(O)CC3=O)C3=C4O)C4=C2O)C(OC)=CC5=CC6=C(C(O)=C51)C(C[C@](O)(C)C6)=O	Flavomannin-6,6´-di-*O*-methyl ether (In-house library) [2]
			COC1=CC2=C(C3=C(C(=O)CC(C)(O)C3)C(O)=C2C(O)=C1)C1=C2C=C(OC)C=C(O)C2=C(O)C2=C1CC(C)(O)CC2=O [1]	
			CC1(CC2=C(C(=O)C1)C(=C3C(=C2)C=C(C(=C3O)C4=C(C5=C(C6=C(CC(CC6=O)(C)O)C=C5C=C4OC)O)O)OC)O)O [20]	
**101**	587.1953	3.65		Flavomannin-6,6´,8-tri-O-methyl ether (ISDB-DNP-Taxo) [2]
			COC1=CC2=C(C3=C(C(=O)CC(C)(O)C3)C(O)=C2C(O)=C1)C1=C2CC(C)(O)CC(=O)C2=C(O)C2=C(O)C(C)=C(OC)C=C12 [1]	
			C[C@@]1(CC2=C(C3=CC(=CC(=C3C(=C2C(=O)C1)O)O)OC)C4=C5C[C@@](CC(=O)C5=C(C6=C(C=C(C=C46)OC)OC)O)(C)O)O [2]	
**3380**	573.1780	3.99		Occidentalol II (ISDB-DNP-Taxo) [2]
			COC1=CC2=C(C3=C(C(=O)CC(C)(O)C3)C(O)=C2C(O)=C1)C1=C2C=C(OC)C=C(O)C2=C(O)C2=C1CC(C)(O)CC2=O [1]	
			CC1(CC2=C(C3=CC(=CC(=C3C(=C2C(=O)C1)O)O)OC)C4=C5CC(CC(=O)C5=C(C6=C(C=C(C=C46)OC)O)O)(C)O)O [1]	
**3382**	587.1954	3.65		Flavomannin-6,6´,8-tri-O-methyl ether (ISDB-DNP-Taxo) [2]
			COC1=CC2=C(C3=C(C(=O)CC(C)(O)C3)C(O)=C2C(O)=C1)C1=C2CC(C)(O)CC(=O)C2=C(O)C2=C(O)C(C)=C(OC)C=C12 [1]	
			C[C@@]1(CC2=C(C3=CC(=CC(=C3C(=C2C(=O)C1)O)O)OC)C4=C5C[C@@](CC(=O)C5=C(C6=C(C=C(C=C46)OC)OC)O)(C)O)O [2]	
**10**	569.1472	4.96	OC1=C(C2=C(OC)C=C(C=C(C(C(C)=CC3=O)=O)C3=C4O)C4=C2O)C(OC)=CC5=CC6=C(C(O)=C51)C(CC(O)(C)C6)=O	Anhydroflavomannin-1,4-quinone-6,6´-di-*O*-methyl ether (In-house library) [2]
			COC1=C(C=CC(O)=C1O)C1C(C(=O)C2=CC=C(O)C=C2)C(=CC2=CC(O)=C(O)C(OC)=C12)C(=O)C1=CC=C(O)C=C1 [1]	
			CC1=C(C=C2C(C3=CC(=C(C(=C3C(=O)C2=C1O)O)C)O)C4C5=CC(=C(C(=C5C(=O)C6=C(C(=C(C=C46)O)C)O)O)C)O)O [2]	

3.4.4.1.3 Cluster C

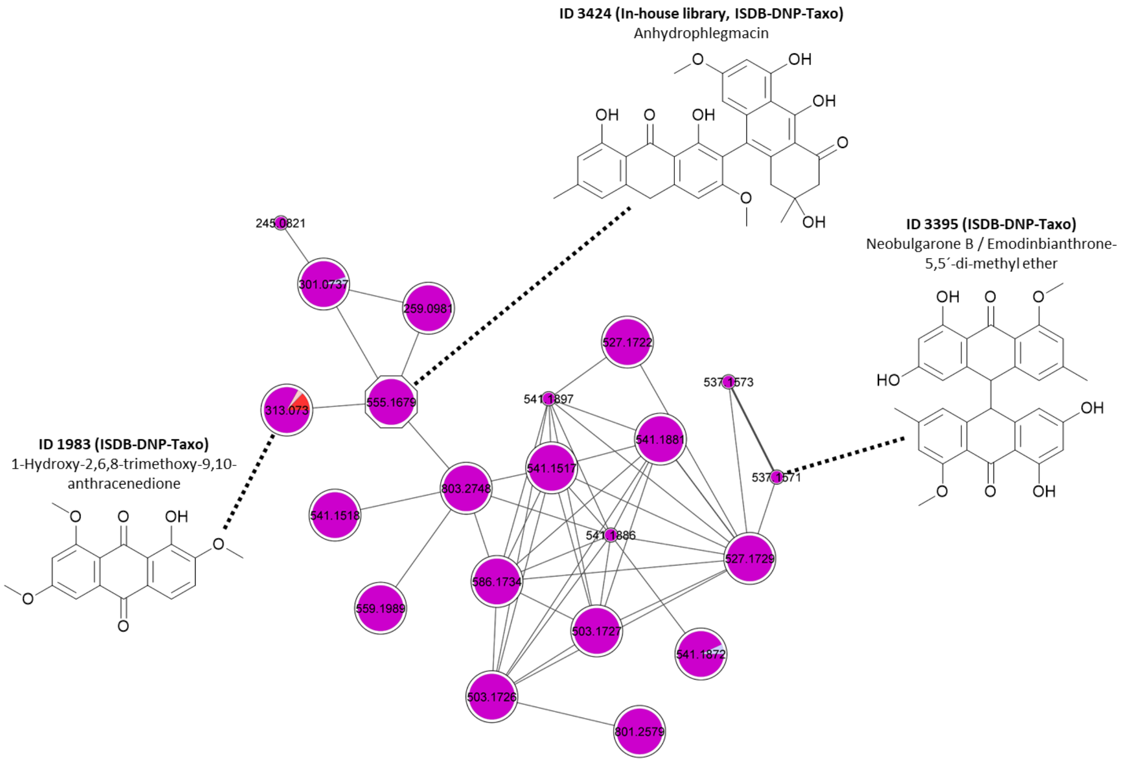

Figure S16. Annotated Cluster C. Each node displays the precursor mass. Feature annotations belonging to the family of Cortinariaceae are presented as octagons. Out of the different molecule annotations listed in the table below (Table S7), the chemical structure was depicted, which was deemed most probable.

Table S7. The annotation results for Cluster C presented as the respective ID (shared name), the Row m/z, the Row RT in minutes, the SMILES (top → bottom: In-house library, GNPS, ISDB-DNP-Taxo, Sirius; Black color … SMILES belonging to the chemical structure drawn in the figure, Grey color … additional hits, Empty … no hit at all), and the name.

ID (shared name)	Row m/z	Row RT [min]	SMILES (In-house library | GNPS | ISDB-DNP-Taxo | Sirius) [Rank]	Name [Identification level]
3395	537.1571	5.93		Neobulgarone B / Emodinbianthrone-5,5´-di-methyl ether (ISDB-DNP-Taxo) [2]
			COC1=CC(C)=CC2=C1C(=O)C1=C(C=C(O)C=C1O)C2C1C2=C(C(OC)=CC(C)=C2)C(=O)C2=C1C=C(O)C=C2O [1]	
			COC1=CC(=C(C2=C1C3=CC(=C(C=C3C=C2)OC)O)C4=C(C=C(C5=C4C=CC6=CC(=C(C=C65)O)OC)OC)O)O [3]	
**1983**	313.0730	3.67		1-Hydroxy-2,6,8-trimethoxy-9,10-anthracenedione (ISDB-DNP-Taxo) [2]
			COC1=C(C(=C2C(=C1)OC(=CC2=O)C3=CC=C(C=C3)O)O)OC	
			COC1=CC2=C(C(=O)C3=C(C=CC(OC)=C3O)C2=O)C(OC)=C1 [2]	
			COC1=CC(=C2C(=C1)C(=O)C3=CC(=CC(=C3C2=O)OC)OC)O [1]	
**3424**	555.1679	4.27	O=C1CC(C)(O)CC2=C(C3=C(O)C4=C(CC(C=C(C)C=C5O)=C5C4=O)C=C3OC)C6=CC(OC)=CC(O)=C6C(O)=C21	Anhydrophlegmacin (In-house library, ISDB-DNP-Taxo) [2]
			COC1=CC2=C(C3=C(C(=O)CC(C)(O)C3)C(O)=C2C(O)=C1)C1=C(OC)C=C2CC3=C(C(O)=CC(C)=C3)C(=O)C2=C1O [2]	
			CC1=CC(=C2C(=C1)CC3=CC(=C(C(=C3C2=O)O)C4=C(C=C(C5=C(C6=C(CC(CC6=O)(C)O)C=C54)O)O)OC)OC)O [2]	

3.4.4.1.4 Cluster D – Chlorinated anthraquinones

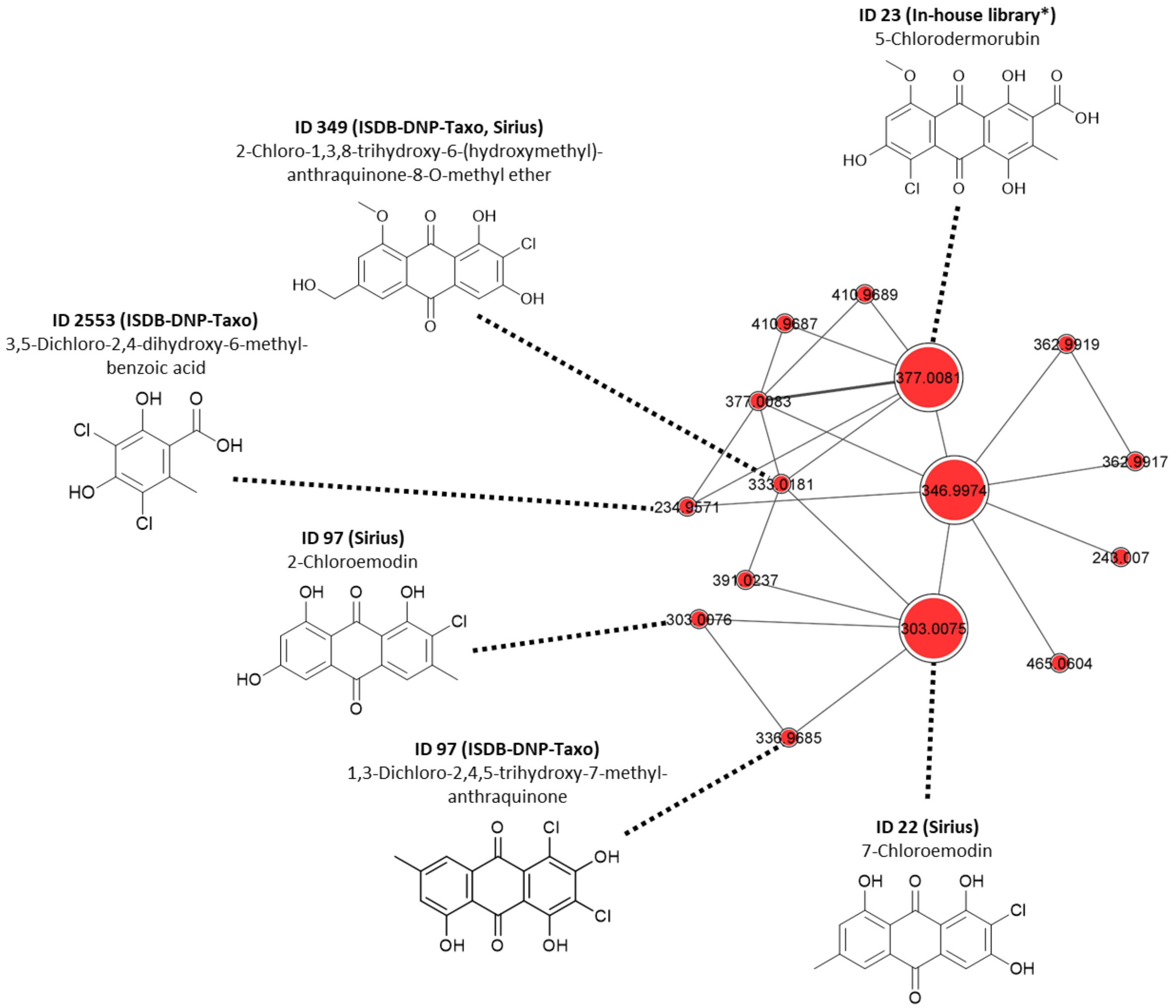

Figure S17. Annotated Cluster D. Each node displays the precursor mass. Out of the different molecule annotations listed in the table below (Table S8), the chemical structure was depicted, which was deemed most probable.

Table S8. The annotation results for Cluster D presented as the respective ID (shared name), the Row m/z, the Row RT in minutes, the SMILES (top → bottom: In-house library, GNPS, ISDB-DNP-Taxo, Sirius; Black color … SMILES belonging to the chemical structure drawn in the figure, Grey color … additional hits, Empty … no hit at all), and the name. Isolated compounds are highlighted in red color.

ID (shared name)	Row m/z	Row RT [min]	SMILES (In-house library | GNPS |ISDB-DNP-Taxo | Sirius) [Rank]	Name [Identification level]
**2553**	234.9571	3.29		3,5-Dichloro-2,4-dihydroxy-6-methylbenzoic acid (ISDB-DNP-Taxo, Sirius) [2]
			CC1=C(C(O)=O)C(O)=C(Cl)C(O)=C1Cl [1]	
			CC1=C(C(O)=O)C(O)=C(Cl)C(O)=C1Cl [1]	
**130**	303.0076	4.12		2-Chloroemodin (Sirius) [2]
			OC1=CC=C(C=C1)C1=COC2=C(C(O)=C(Cl)C(O)=C2)C1=O [1]	
			CC1=C(C(=C2C(=C1)C(=O)C3=CC(=CC(=C3C2=O)O)O)O)Cl [1]	
**349**	333.0181	3.92		2-Chloro-1,3,8-trihydroxy-6-(hydroxymethyl)-anthraquinone-8-*O*-methyl ether (Sirius) [2]
			COC1=CC(CO)=CC2=C1C(=O)C1=C(O)C(Cl)=C(O)C=C1C2=O [1]	
			COC1=C2C(=CC(=C1)CO)C(=O)C3=CC(=C(C(=C3C2=O)O)Cl)O [1]	
**22**	303.0075	4.27		7-Chloroemodin (Sirius) [2]
			OC1=CC=C(C=C1)C1=COC2=C(C(O)=CC(O)=C2Cl)C1=O [1]	
			CC1=CC(=C2C(=C1)C(=O)C3=CC(=C(C(=C3C2=O)O)Cl)O)O [2]	
**23**	377.0081	2.82	O=C1C2=C(C(O)=C(C)C(C(O)=O)=C2O)C(C3=C(Cl)C(O)=CC(OC)=C31)=O	5-Chlorodermorubin (In-house library) [1]
			CC1=CN(C(=O)NC1=O)CCOCP(=O)(CP(=O)(O)O)O [1]	
**97**	336.9685	4.43		1,3-Dichloro-2,4,5-trihydroxy-7-methylanthraquinone (ISDB-DNP-Taxo) [2]
			CC1=CC2=C(C(O)=C1)C(=O)C1=C(C(Cl)=C(O)C(Cl)=C1O)C2=O [1]	
			CC1=C2C(=CC(=C1Cl)O)C(=O)C3=C(C2=O)C(=CC(=C3Cl)O)O [1]	

3.4.4.1.5 Cluster E – Glycosylated anthraquinones

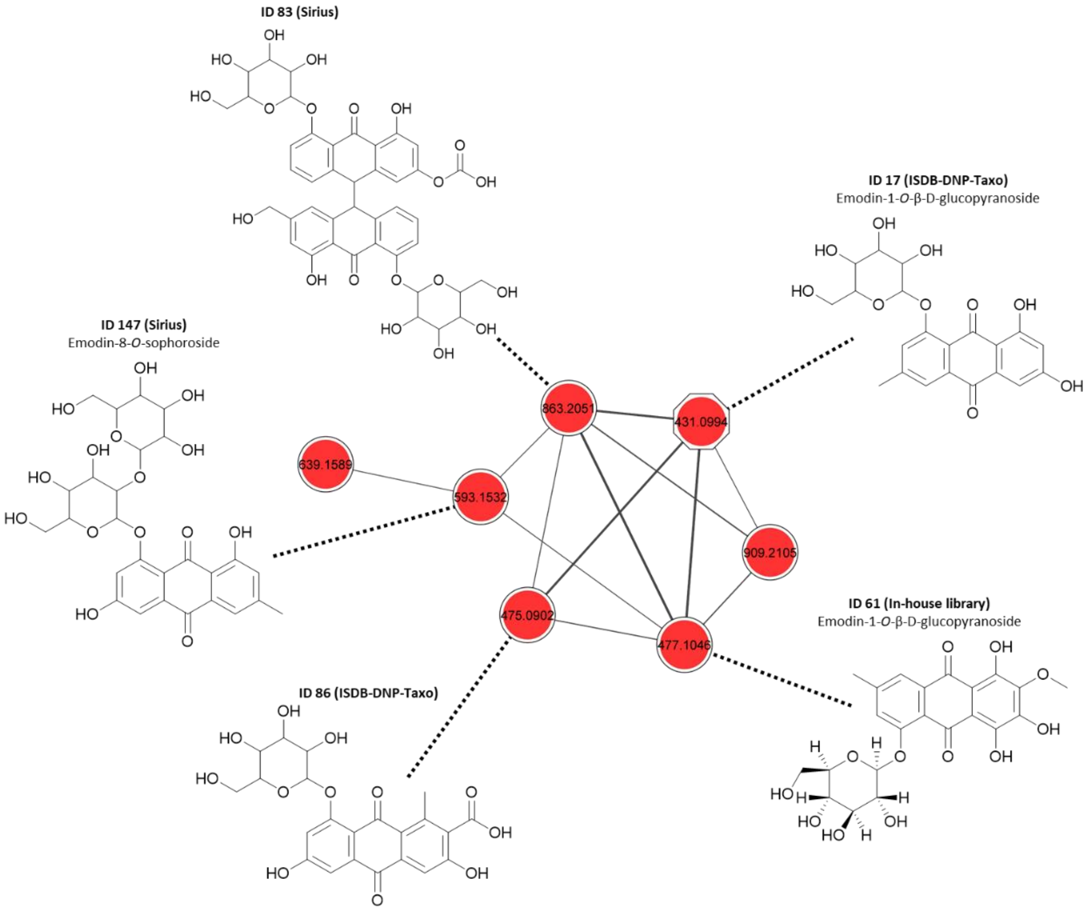

Figure S18. Annotated Cluster E. Each node displays the precursor mass. Feature annotations belonging to the family of Cortinariaceae are presented as octagons. Out of the different molecule annotations listed in the table below (Table S9), the chemical structure was depicted, which was deemed most probable.

Table S9. The annotation results for Cluster E presented as the respective ID (shared name), the Row m/z, the Row RT in minutes, the SMILES (top → bottom: In-house library, GNPS, ISDB-DNP-Taxo, Sirius; Black color … SMILES belonging to the chemical structure drawn in the figure, Grey color … additional hits, Empty … no hit at all), and the name.

ID (shared name)	Row m/z	Row RT [min]	SMILES (In-house library | GNPS | ISDB-DNP-Taxo | Sirius) [Rank]	Name [Identification level]
**17**	431.0994	2.85		Emodin-1-*O*-β-D-glucopyranoside (ISDB-DNP-Taxo) [2], Emodin-6-*O*- β-D-glucopyranoside (Sirius) [2]
			CC1=CC2=C(C(=O)C3=C(C=C(O)C=C3O)C2=O)C(OC2OC(CO)C(O)C(O)C2O)=C1 [1]	
			CC1=CC(=C2C(=C1)C(=O)C3=CC(=CC(=C3C2=O)O)OC4C(C(C(C(O4)CO)O)O)O)O [1]	
**83**	863.2051	2.85		4,4'-dihydroxy-2'-(hydroxymethyl)-10,10'-dioxo-5,5'-bis((3,4,5-trihydroxy-6-(hydroxymethyl)tetrahydro-2H-pyran-2-yl)oxy)-9,9',10,10'-tetrahydro-[9,9'-bianthracen]-2-yl hydrogen carbonate (Sirius) [2]
			C1=CC2=C(C(=C1)OC3C(C(C(C(O3)CO)O)O)O)C(=O)C4=C(C=C(C=C4C2C5C6=C(C(=CC=C6)OC7C(C(C(C(O7)CO)O)O)O)C(=O)C8=C(C=C(C=C58)OC(=O)O)O)CO)O [1]	
**86**	475.0902	1.72		2-Anthracenecarboxylic acid, 8-(β-D-glucopyranosyloxy)-9, 10-dihydro-3,6-dihydroxy-1-methyl-9, 10-dioxo- (ISDB-DNP-Taxo) [2]
			CC1=C2C(=O)C3=C(C=C(O)C=C3OC3OC(CO)C(O)C(O)C3O)C(=O)C2=CC(O)=C1C(O)=O [1]	
			CC1=C2C(=CC(=C1C(=O)O)O)C(=O)C3=CC(=CC(=C3C2=O)O)OC4C(C(C(C(O4)CO)O)O)O [1]	
**147**	593.1532	1.54		Emodin-8-*O*-sophoroside (Sirius) [2]
			CC1=CC2=C(C(=O)C3=C(C=C(O)C=C3O)C2=O)C(OC2OC(CO)C(O)C(O)C2OC2OC(CO)C(O)C(O)C2O)=C1 [2]	
			CC1=CC(=C2C(=C1)C(=O)C3=CC(=CC(=C3C2=O)OC4C(C(C(C(O4)CO)O)O)OC5C(C(C(C(O5)CO)O)O)O)O)O [3]	
**61**	477.1046	2.86	O=C1C2=C(C=C(C)C=C2O[C@@]([H])([C@](O)3[H])O[C@]([H])(CO)[C@@]([H])(O)[C@@]3(O)[H])C(C4=C(O)C(OC)=C(O)C(O)=C41)=O	Dermocybin-1- β-D-glucopyranoside (In-house library) [2]
			OCC1OC(OC2=CC(=CC(C(O)=O)=C2C(=O)CC2=CC=C(O)C=C2)C(O)=O)C(O)C(O)C1O [1]	
			CC1=C(C(=C2C(=C1)C(=O)C3=CC(=C(C(=C3C2=O)O)OC)O)O)OC4C(C(C(C(O4)CO)O)O)O [1]	

3.4.4.1.6 Cluster F – Dimeric anthraquinone-like structures

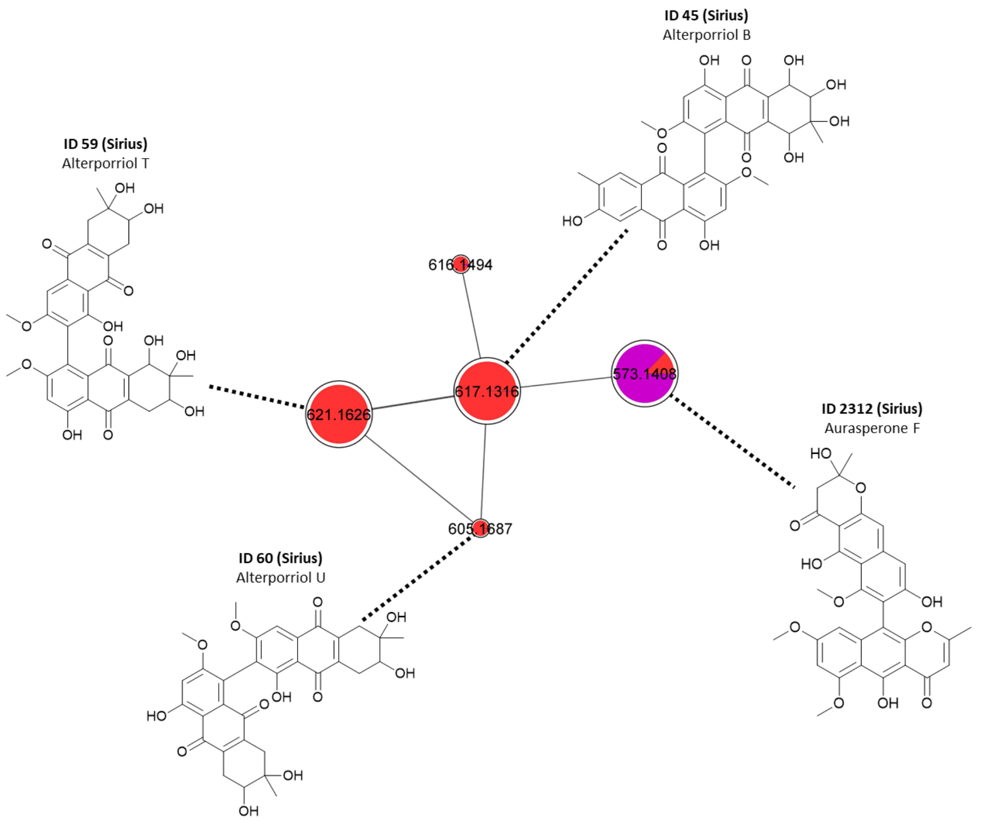

Figure S19. Annotated Cluster F. Each node displays the precursor mass. Out of the different molecule annotations listed in the table below (Table S10), the chemical structure was depicted, which was deemed most probable.

Table S10. The annotation results for Cluster F presented as the respective ID (shared name), the Row m/z, the Row RT in minutes, the SMILES (In-house library, GNPS, ISDB-DNP-Taxo, Sirius; Black color … SMILES belonging to the chemical structure drawn in the figure, Grey color … additional hits, Empty … no hit at all), and the name.

ID (shared name)	Row m/z	Row RT [min]	SMILES (In-house library | GNPS | ISDB-DNP-Taxo | Sirius) [Rank]	Name [Identification level]
**59**	621.1626	3.13		Alterporriol T (Sirius) [2]
			CC12CC3(OC1=O)C1=CC(=O)C4C5(C)CC(Br)(Br)C(=O)C(C)(C)C5CCC4(C)C1(C)CCC3(C)CC2 [1]	
			CC1(CC2=C(CC1O)C(=O)C3=C(C(=C(C=C3C2=O)OC)C4=C5C(=C(C=C4OC)O)C(=O)C6=C(C5=O)C(C(C(C6)O)(C)O)O)O)O [5]	
**60**	605.1687	3.07		Alterporriol U (Sirius) [2]
			CC1(CC2=C(CC1O)C(=O)C3=C(C(=C(C=C3C2=O)OC)C4=C5C(=C(C=C4OC)O)C(=O)C6=C(C5=O)CC(C(C6)O)(C)O)O)O [13]	
**2312**	573.1408	4.85		Aurasperone F (Sirius) [2]
			COC1=CC2=C(C(O)=C3C(=O)C=C(C)OC3=C2C2=C(O)C=C3C=C4OC(C)(O)CC(=O)C4=C(O)C3=C2OC)C(OC)=C1 [1]	
			CC1=CC(=O)C2=C(C3=C(C=C(C=C3C(=C2O1)C4=C(C5=C(C6=C(C=C5C=C4O)OC(CC6=O)(C)O)O)OC)OC)OC)O [3]	
**45**	617.1316	4.38		Alterporriol B (Sirius) [2]
			CC1=C(C=C2C(=C1)C(=O)C3=C(C(=CC(=C3C2=O)O)OC)C4=C5C(=C(C=C4OC)O)C(=O)C6=C(C5=O)C(C(C(C6O)O)(C)O)O)O [2]	

3.4.4.1.7 Cluster G – Glycosylated & methylated anthraquinones

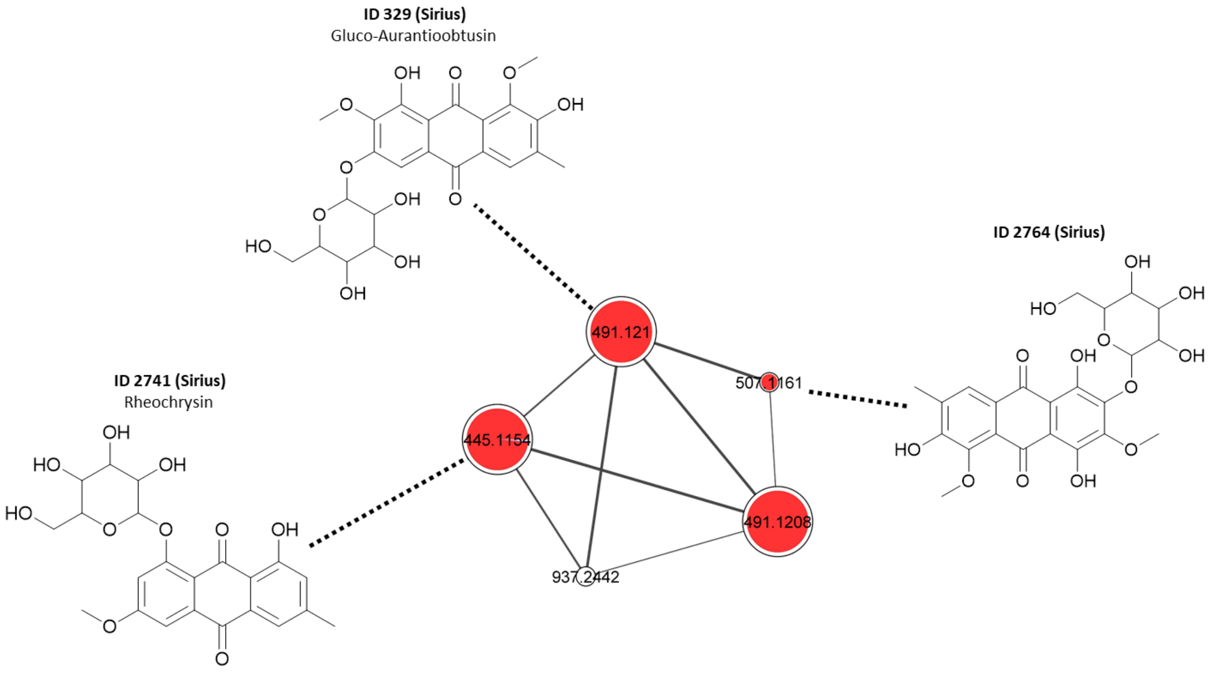

Figure S20. Annotated Cluster G. Each node displays the precursor mass. Out of the different molecule annotations listed in the table below (Table S11), the chemical structure was depicted, which was deemed most probable.

Table S11. The annotation results for Cluster G presented as the respective ID (shared name), the Row m/z, the Row RT in minutes, the SMILES (top → bottom: In-house library, GNPS, ISDB-DNP-Taxo, Sirius; Black color … SMILES belonging to the chemical structure drawn in the figure, Grey color … additional hits, Empty … no hit at all), and the name.

ID (shared name)	Row m/z	Row RT [min]	SMILES (In-house library | GNPS | ISDB-DNP-Taxo | Sirius) [Rank]	Name [Identification level]
**2028**	491.1207	2.36		Gluco-Aurantioobtusin (Sirius) [2]
			COC1=CC(=CC(O)=C1OC)C1=COC2=CC(OC3OC(CO)C(O)C(O)C3O)=CC(O)=C2C1=O [1]	
			CC1=C(C(=C2C(=C1)C(=O)C3=CC(=C(C(=C3C2=O)O)OC)OC4C(C(C(C(O4)CO)O)O)O)OC)O [1]	
**2764**	507.1161	2.69		1,4,6-trihydroxy-3,5-dimethoxy-7-methyl-2-((3,4,5-trihydroxy-6-(hydroxymethyl)tetrahydro-2H-pyran-2-yl)oxy)anthracene-9,10-dione [2]
			COC1=C(O)C=C(C=C1O)C1=COC2=C(O)C(OC)=C(OC3OC(CO)C(O)C(O)C3O)C=C2C1=O [1]	
			CC1=C(C(=C2C(=C1)C(=O)C3=C(C2=O)C(=C(C(=C3O)OC4C(C(C(C(O4)CO)O)O)O)OC)O)OC)O [3]	
**2741**	445.1154	2.37		Rheochrysin (Sirius) [2]
			CC1=CC(=CC=C1O)C1=CC(=O)C2=C(O)C=C(OC3OC(CO)C(O)C(O)C3O)C=C2O1 [1]	
			CC1=CC(=C2C(=C1)C(=O)C3=CC(=CC(=C3C2=O)OC4C(C(C(C(O4)CO)O)O)O)OC)O [3]	
**329**	491.1210	2.59		Gluco-Aurantioobtusin (Sirius) [2]
			COC1=CC(=CC(O)=C1OC)C1=COC2=CC(OC3OC(CO)C(O)C(O)C3O)=CC(O)=C2C1=O [1]	
			CC1=C(C(=C2C(=C1)C(=O)C3=CC(=C(C(=C3C2=O)O)OC)OC4C(C(C(C(O4)CO)O)O)O)OC)O [1]	

3.4.4.1.8 Cluster H – Monomeric anthraquinones (methylated / esterified)

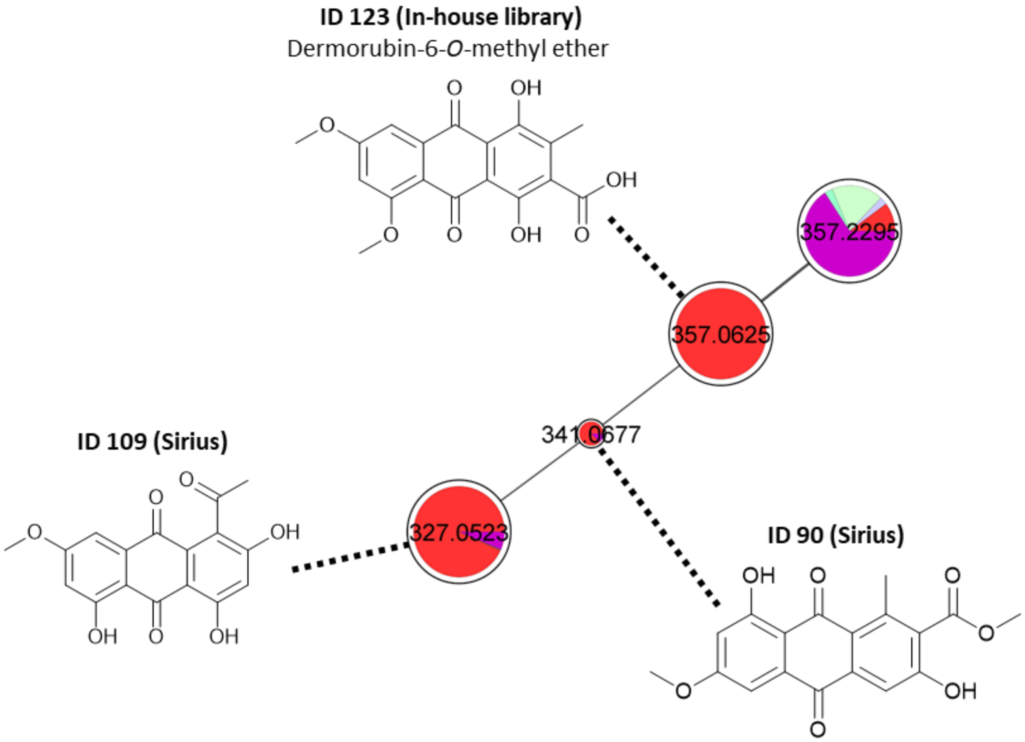

Figure S21. Annotated Cluster H. Each node displays the precursor mass. Out of the different molecule annotations listed in the table below (Table S12), the chemical structure was depicted, which was deemed most probable.

Table S12. The annotation results for Cluster H presented as the respective ID (shared name), the Row m/z, the Row RT in minutes, the SMILES (top → bottom: In-house library, GNPS, ISDB-DNP-Taxo, Sirius; Black color … SMILES belonging to the chemical structure drawn in the figure, Grey color … additional hits, Empty … no hit at all), and the name.

ID (shared name)	Row m/z	Row RT [min]	SMILES (In-house library | GNPS | ISDB-DNP-Taxo | Sirius) [Rank]	Name [Identification level]
**123**	357.0624	3.60	O=C1C2=C(OC)C=C(OC)C=C2C(C3=C1C(O)=C(C(O)=O)C(C)=C3O)=O<br><br>COC(=O)C1=C(O)C2=C(C(O)=C1C)C(=O)C1=C(C(O)=CC(OC)=C1)C2=O [3]<br>CC1=C(C2=C(C(=C1C(=O)OC)O)C(=O)C3=C(C=C(C=C3C2=O)OC)O)O [3]	Dermorubin-6-*O*-methyl ether (In-house library) [2], Cinnarubin methyl ester (Sirius) [2]
**109**	327.0522	3.73	 <br>COC(=O)C1=C2OC3=C(C4=C(C(=O)OC4)C(O)=C3)C2=C(O)C=C1C [1]<br>CC(=O)C1=C(C=C(C2=C1C(=O)C3=CC(=CC(=C3C2=O)O)OC)O)O [3]	9,10-Anthracenedione, 1-acetyl-2,4,5-trihydroxy-7-methoxy- [2}
**90**	341.0676	3.33	 <br>COC(=O)C1=CC(OC)=C2C(=O)C3=C(C=CC=C3OC2=C1)C(=O)OC [1]<br>CC1=C2C(=CC(=C1C(=O)OC)O)C(=O)C3=CC(=CC(=C3C2=O)O)OC [1]	Methyl 3,8-dihydroxy-6-methoxy-1-methylanthraquinone-2-carboxylate [2]

3.4.4.1.9 Cluster I – Emodin

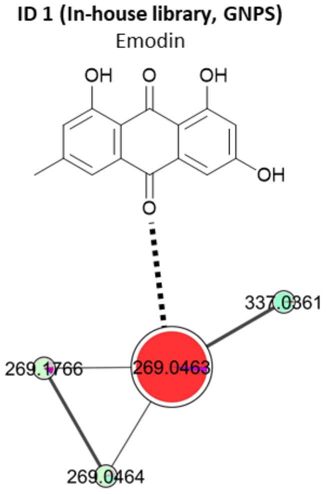

Figure S22. Annotated Cluster I. Each node displays the precursor mass. Out of the different molecule annotations listed in the table below (Table S13), the chemical structure was depicted, which was deemed most probable.

Table S13. The annotation results for Cluster I presented as the respective ID (shared name), the Row m/z, the Row RT in minutes, the SMILES (top → bottom: In-house library, GNPS, ISDB-DNP-Taxo, Sirius; Black color … SMILES belonging to the chemical structure drawn in the figure, Grey color … additional hits, Empty … no hit at all), and the name.

ID (shared name)	Row m/z	Row RT [min]	SMILES (In-house library | GNPS | ISDB-DNP-Taxo | Sirius) [Rank]	Name [Identification level]
**1**	269.0462	4.01	O=C1C2=C(O)C=C(O)C=C2C(C3=C1C(O)=CC(C)=C3)=O	Emodin (In-house library) [2]
			O=C1C2=C(O)C=C(O)C=C2C(C3=C1C(O)=CC(C)=C3)=O	
			COC(=O)C1=C(O)C2=C(C(O)=C1C)C(=O)C1=C(C(O)=CC(OC)=C1)C2=O [3]	

3.4.4.1.10 Cluster J – Anhydrophlegmacin family

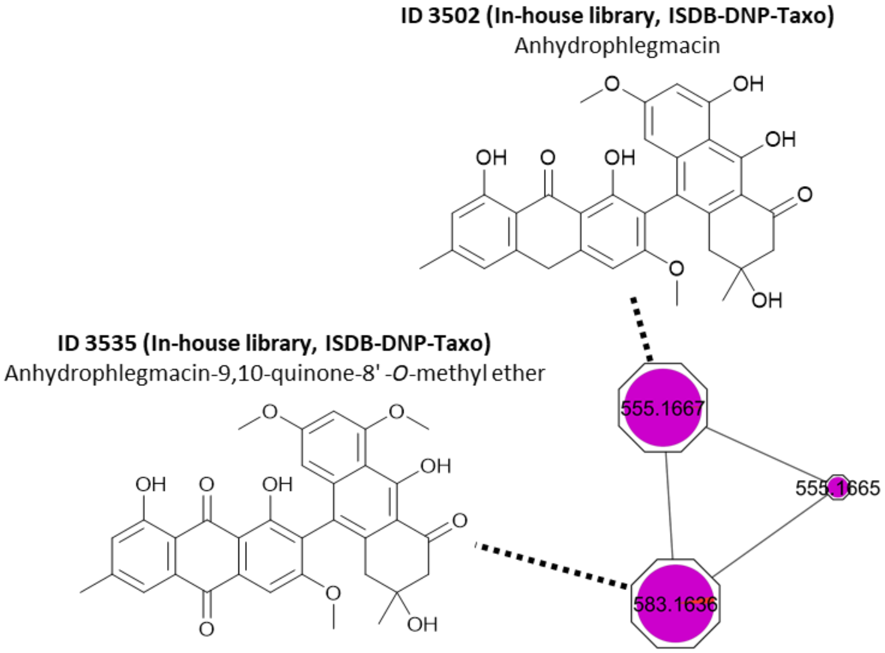

Figure S23. Annotated Cluster J. Each node displays the precursor mass. Feature annotations belonging to the family of Cortinariaceae are presented as octagons. Out of the different molecule annotations listed in the table below (Table S14), the chemical structure was depicted, which was deemed most probable.

Table S14. The annotation results of Cluster J presented as the respective ID (shared name), the Row m/z, the Row RT in minutes, the SMILES (top → bottom: In-house library, GNPS, ISDB-DNP-Taxo, Sirius; Black color … SMILES belonging to the chemical structure drawn in the figure, Grey color … additional hits, Empty … no hit at all), and the name.

ID (shared name)	Row m/z	Row RT [min]	SMILES (In-house library | GNPS | ISDB-DNP-Taxo | Sirius) [Rank]	Name [Identification level]
**3502**	555.1667	3.97	O=C1CC(C)(O)CC2=C(C3=C(O)C4=C(CC(C=C(C)C=C5O)=C5C4=O)C=C3OC)C6=CC(OC)=CC(O)=C6C(O)=C21<br><br>COC1=CC2=C(C3=C(C(=O)CC(C)(O)C3)C(O)=C2C(O)=C1)C1=C(OC)C=C2CC3=C(C(O)=CC(C)=C3)C(=O)C2=C1O [4]<br>CC1=CC(=C2C(=C1)CC3=CC(=C(C(=C3C2=O)O)C4=C(C=C(C5=C(C6=C(CC(CC6=O)(C)O)C=C54)O)O)OC)OC)O [1]	Anhydrophlegmacin (In-house library, ISDB-DNP-Taxo) [2]
**3535**	583.1635	4.81	O=C1CC(C)(O)CC2=C(C3=C(O)C4=C(C(C(C=C(C)C=C5O)=C5C4=O)=O)C=C3OC)C6=CC(OC)=CC(OC)=C6C(O)=C21<br><br>COC1=CC2=C(C3=C(C(=O)CC(C)(O)C3)C(O)=C2C(OC)=C1)C1=C(OC)C=C2C(=O)C3=C(C(O)=CC(C)=C3)C(=O)C2=C1O [3]<br>CC1=CC(=C2C(=C1)C(=O)C3=C(C(=CC(=C3C2=O)O)OC)C4=C5CC(CC(=O)C5=C(C6=C(C=C(C=C46)OC)OC)O)(C)O)O [1]	Anhydrophlegmacin-9,10-quinone-8' -*O*-methyl ether (In-house library, ISDB-DNP-Taxo) [2]
**3714**	555.1665	4.05	O=C1CC(C)(O)CC2=C(C3=C(O)C4=C(CC(C=C(C)C=C5O)=C5C4=O)C=C3OC)C6=CC(OC)=CC(O)=C6C(O)=C21<br><br>COC1=CC2=C(C3=C(C(=O)CC(C)(O)C3)C(O)=C2C(O)=C1)C1=C(OC)C=C2CC3=C(C(O)=CC(C)=C3)C(=O)C2=C1O [3]<br>CC1=CC(=C2C(=C1)CC3=CC(=C(C(=C3C2=O)O)C4=C(C=C(C5=C(C6=C(CC(CC6=O)(C)O)C=C54)O)O)OC)OC)O [1]	Anhydrophlegmacin (In-house library, ISDB-DNP-Taxo) [2]

3.4.5 Specificity of features

The extract specificity of features was calculated as peak area of the respective extract divided by the sum of all peak areas of all extracts. Following specificity levels were investigated: > 60%, > 95%, and > 99%. Additionally, the number of extract specific features absorbing light in the visible range (λ = 468 nm) was investigated. The results are depicted as bar plots generated with the program Origin 2020 (Figure S24, Figure S25, Figure S26).

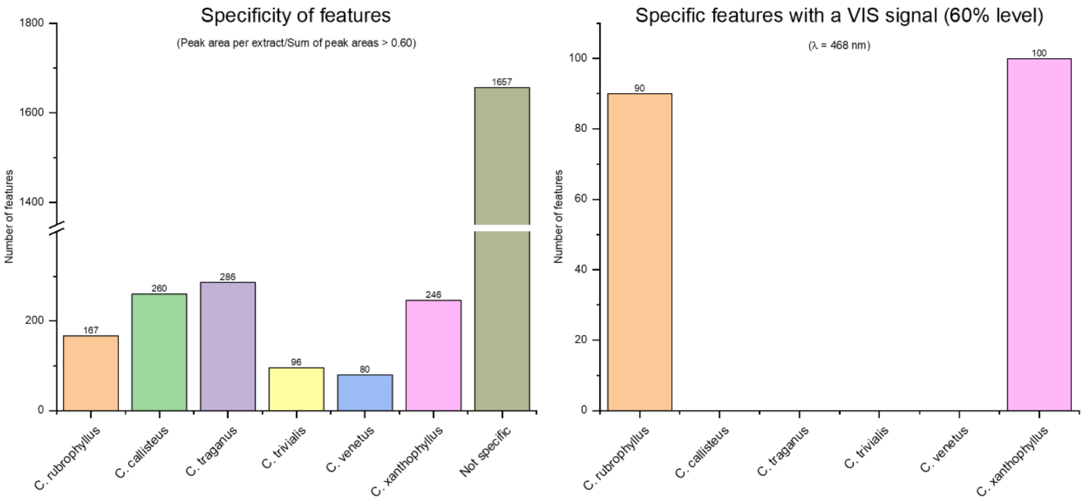

Figure S24. Bar plots representing the specificity of features (level > 60%) (left figure) as well as the extract-specific features that absorb light at 468 nm (right figure). The specificity was calculated as peak area per extract divided by the sum of all peak areas > 60%.

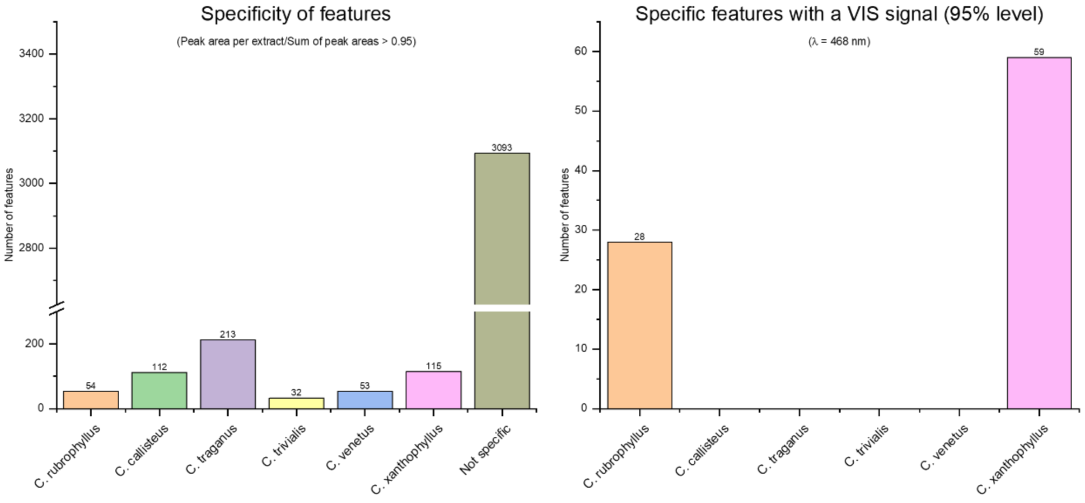

Figure S25. Bar plots representing the specificity of features (level > 95%) (left figure) as well as the extract-specific features that absorb light at 468 nm (right figure). The specificity was calculated as peak area per extract divided by the sum of all peak areas > 95%.

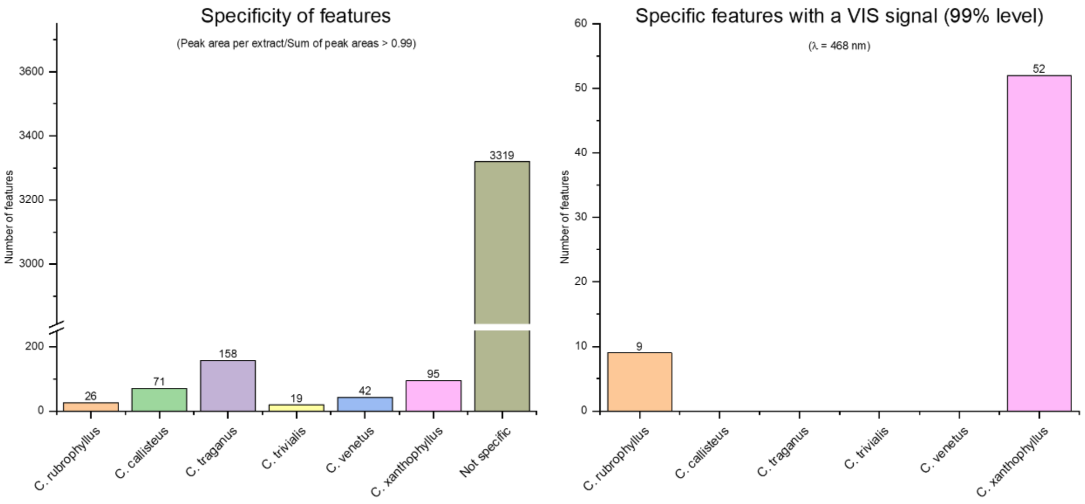

Figure S26. Bar plots representing the specificity of features (level > 99%) (left figure) as well as the extract-specific features that absorb light at 468 nm (right figure). The specificity was calculated as peak area per extract divided by the sum of all peak areas > 99%.

3.4.6 Annotation-hit-rate

The annotation-hit-rate was calculated as number of putative annotations (GNPS = Analog:Compound_Name; DNP = Molecule_Name_DNP) divided by the number of nodes in the FBMN. The figure below represents the results of this investigation (Figure S27).

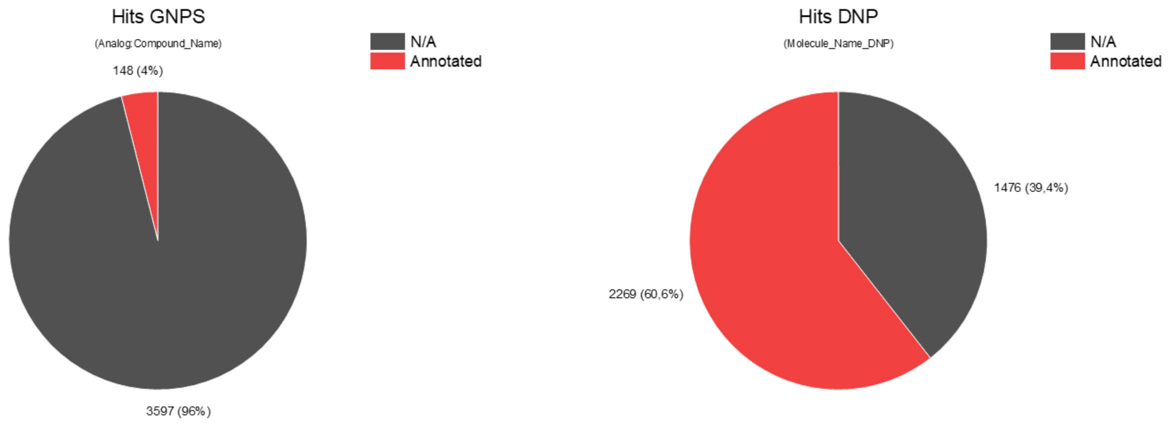

Figure S27. Pie charts depicting the annotation-hit-rates via spectral annotation against the GNPS database and the DNP-ISDB. The hit rates were calculated as number of putative annotations divided by the number of nodes being present in the FBMN.

3.4.7 ISDB-DNP-annotations originating from the family of Cortinariaceae

Using the "Family_cof_DNP"-information generated via spectral annotation against the ISDB-DNP (chapter 3.3) the number of putative annotations associated with the Cortinariaceae family was calculated. The figure below (Figure S28) shows the entire FBMN with 35 nodes highlighted as big red dots as their MS^2 spectra showed spectral similarity towards compounds previously isolated from *Cortinarius* species.

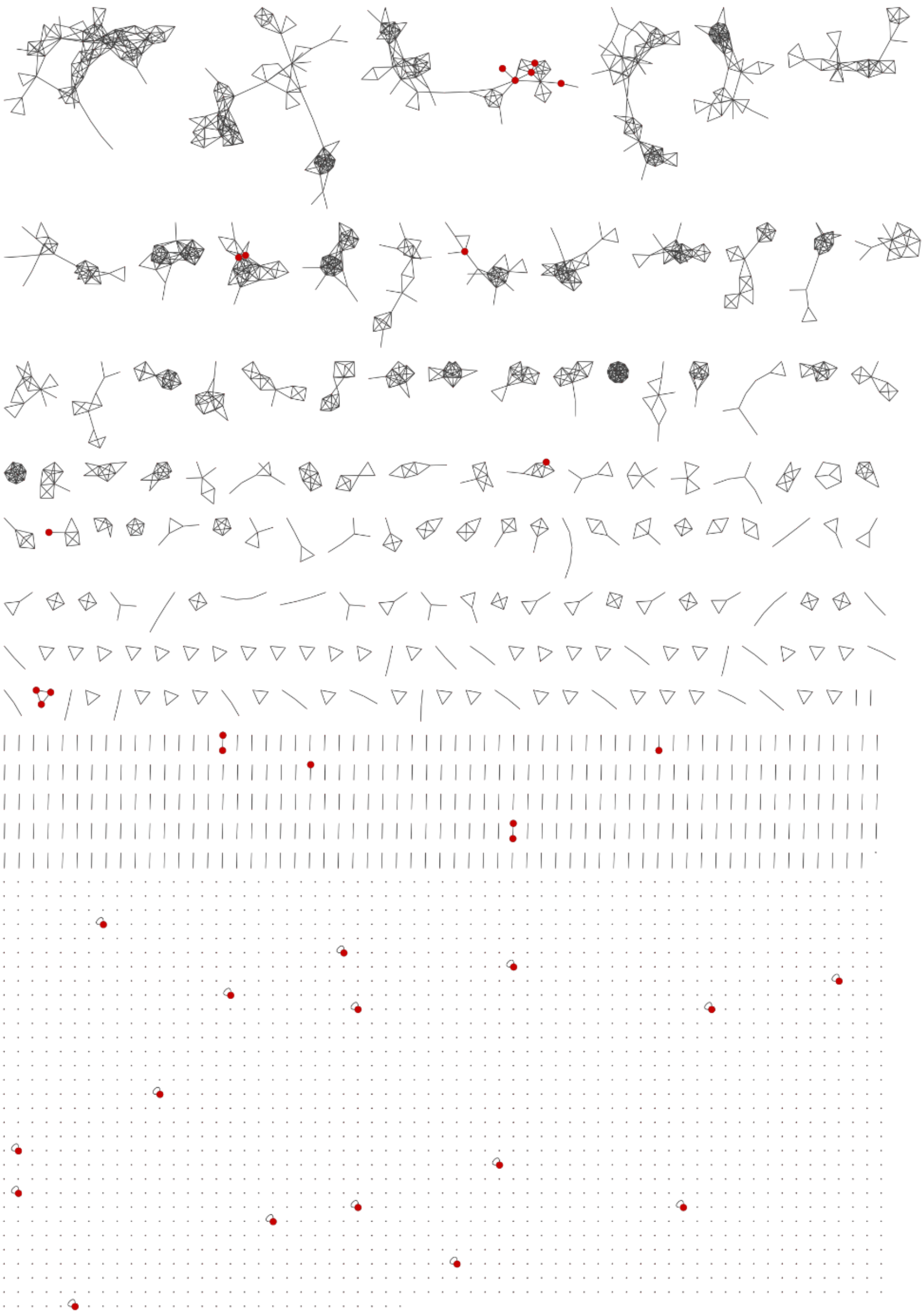

Figure S28. The FBMN with features related to *Cortinarius* species highlighted as big red dots based on the findings from the ISDB-DNP spectral annotation process.

3.4.8 Chemical taxonomy (ClassyFire) representation

The "Consensus_ci_cf"-information generated via taxonomically informed metabolite annotation (ClassyFire; chapter 3.3) was used to calculate the number of nodes associated with different compound classes. The classes being represented the most are as follows: "Fatty Acyls" (268 features, 24.0%). "Benzene and substituted derivatives" (160 features, 14.3%), "Organooxygen compounds" (153 features, 13.7%), "Prenol lipids" (111 derivatives, 10.0%), "Benzopyrans" (59 features. 5.3%), and "Anthracenes" (59 features, 5.3%). The remaining features are distributed between many different compound classes and amount to less than 5.0% (relative to the sum of all nodes in the FBMN), respectively. **Figure S29** (A = pie chart, B = FBMN) depicts the chemical space covering all extracts. The FBMN is visualized with the software Cytoscape (**Figure S29**, B) employing the "Class ClassyFire"-layer, which has been generated in the framework of the chemical taxonomy investigation (Fill color: Column = Consensus_ci_cf, Mapping type = Discrete Mapping, Mapping Value Generators = Rainbow OSC; chapter 3.3).

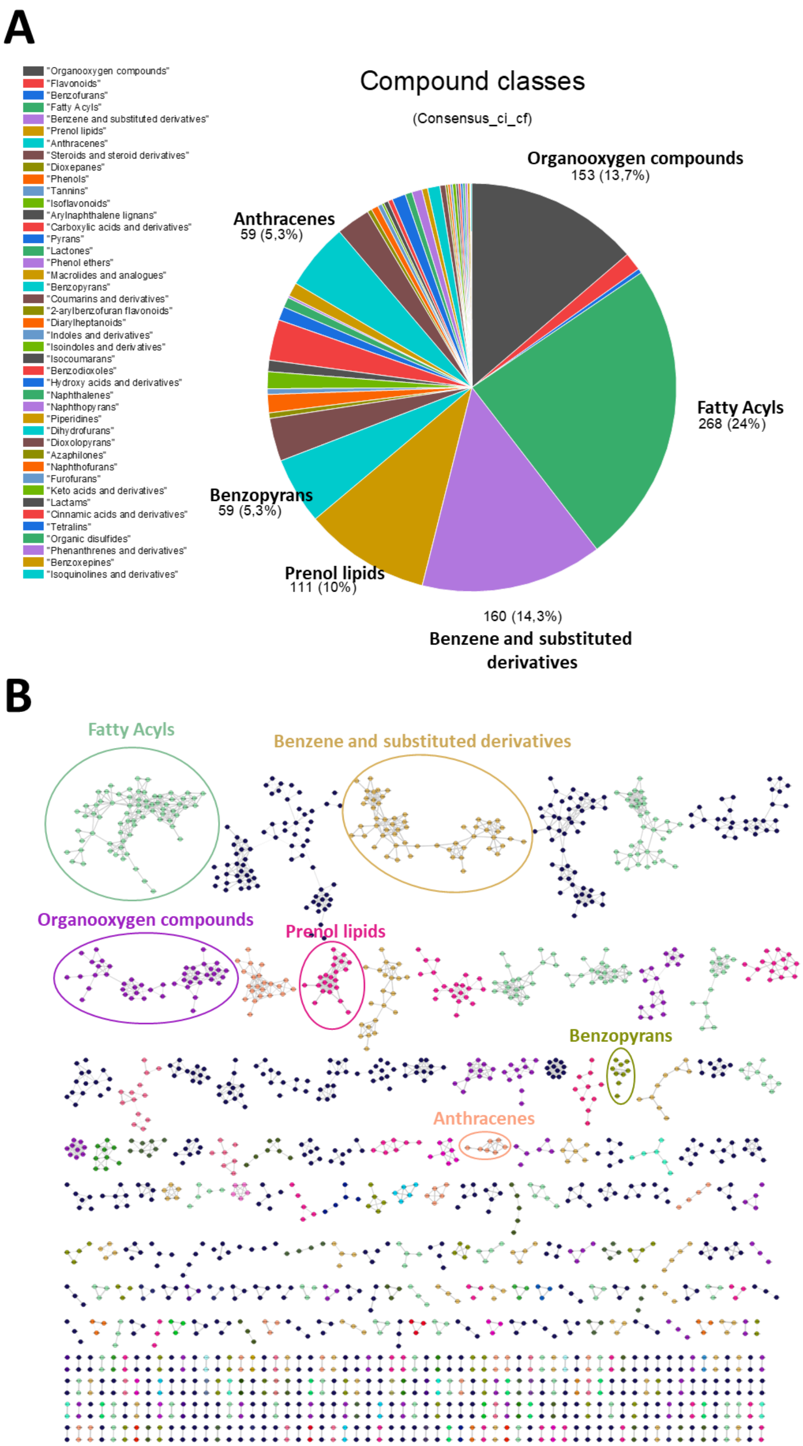

Figure S29. A) Pie chart depicting the "Consensus_ci_cf"-information. Compound classes amounting to more than 5.0% (relative to the total number of nodes) are highlighted. B) The FBMN visualized via Cytoscape embedded with a layer containing the chemical taxonomy information generated with the taxonomically informed metabolite annotation workflow (chapter 3.3). The six most abundant classes ("Fatty Acyls", "Benzene and substituted derivatives", "Organooxygen compounds", "Prenol lipids". "Benzopyrans", and "Anthracenes") are highlighted in color.

3.4.9 Identification of compound classes associated with photoactivity

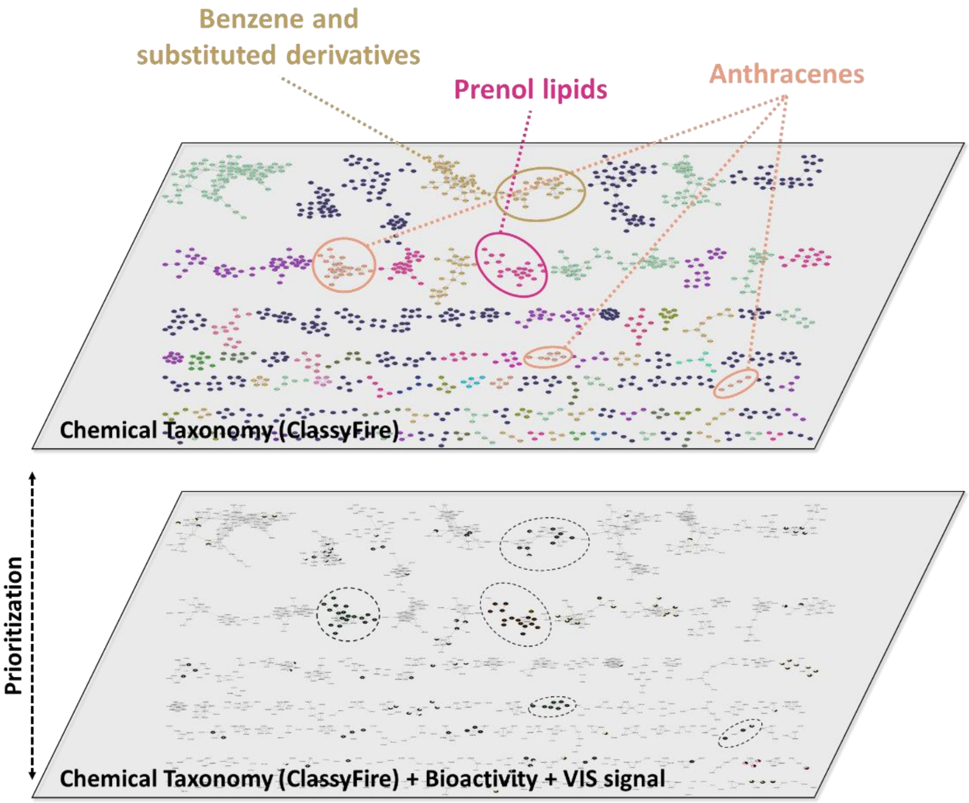

Figure S30. Identification of photoactive compound classes. The information regarding the fungal extracts´ chemical space generated with the taxonomically informed metabolite annotation workflow (ClassyFire; chapter 3.3) was combined with their bioactivity ("Peak area"-variable as ring chart; photoactive = black, photoinactive = white) and the individual features´ ability to absorb light in the visible range ("VIS-Signal"-variable mapped as node size: "Yes" = node size 50, "No" = node size 5).

3.4.10 Active clusters sorted by their polarity

The clusters A-J (chapter 3.4.2) were investigated regarding their polarity. For this purpose, the FBMN was visualized with Cytoscape and the "row retention time"-information was mapped as node fill color. The mapping type was set to continuous (RT range = 0.8544-6.9997 min) and the "Viridis Plasma perceptually balanced palette" was chosen for visual representation. This color code was combined with the solvent gradient used for liquid-chromatographic separation (linear gradient: 0-7 min – 5-100% ACN + 0.1% FA. isocratic step: 7-8 min 100% ACN + 0.1% FA) to depict the polarity of respective features. The figure below (**Figure S31**) shows the FBMN embedded with the described informational layer coupled to the total ion chromatograms resulting from the chromatographic separation of the fungal extracts. A "consensus retention time" was defined for every cluster (A-J) by calculating the mean retention time of all features including their standard deviation for the respective clusters. Clusters showing similar retention times (= similar polarities) were combined and marked in the chromatogram.

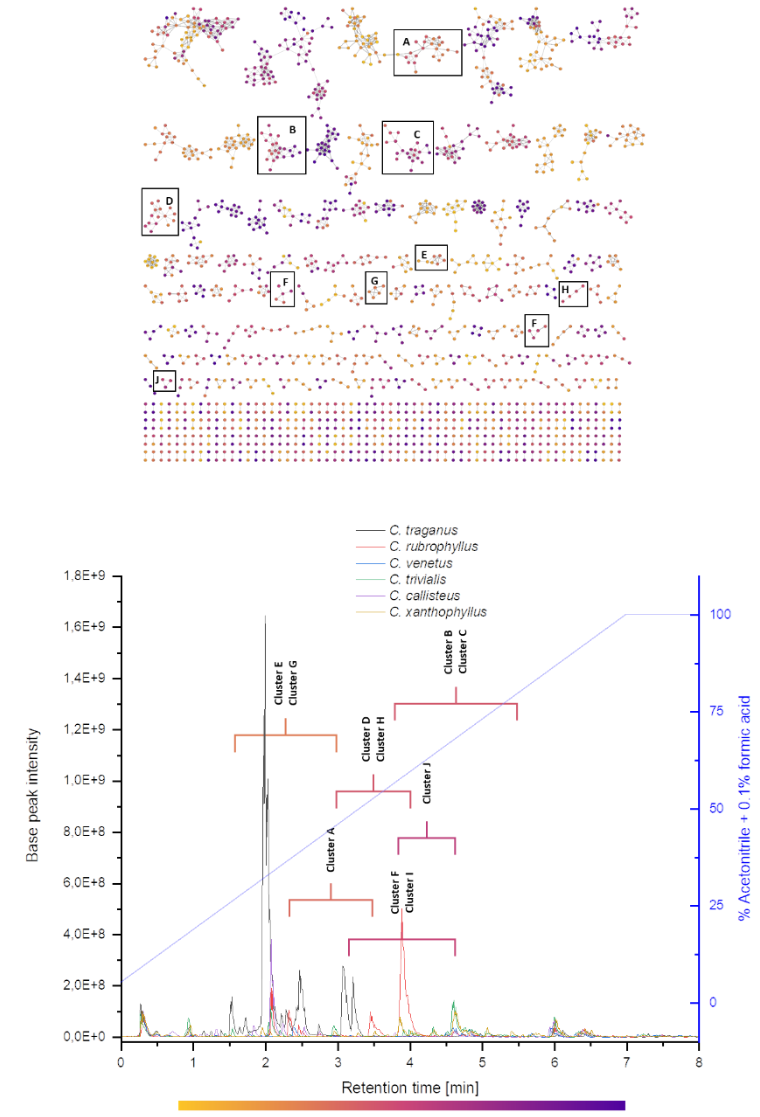

Figure S31. The FBMN embedded with a layer that depicts the features´ polarity (“row retention time”-information). Photoactive clusters (A-J) are highlighted. The consensus polarity of each cluster, which is defined by the “consensus retention time” (mean RT ± standard deviation), is shown in **c**ombination with an overlay of all total ion chromatograms of the fungal extracts. The clusters´ polarity is highlighted with brackets covering their respective retention time-ranges.

3.4.11 Mass spectral data: Cluster D – Chlorinated anthraquinones

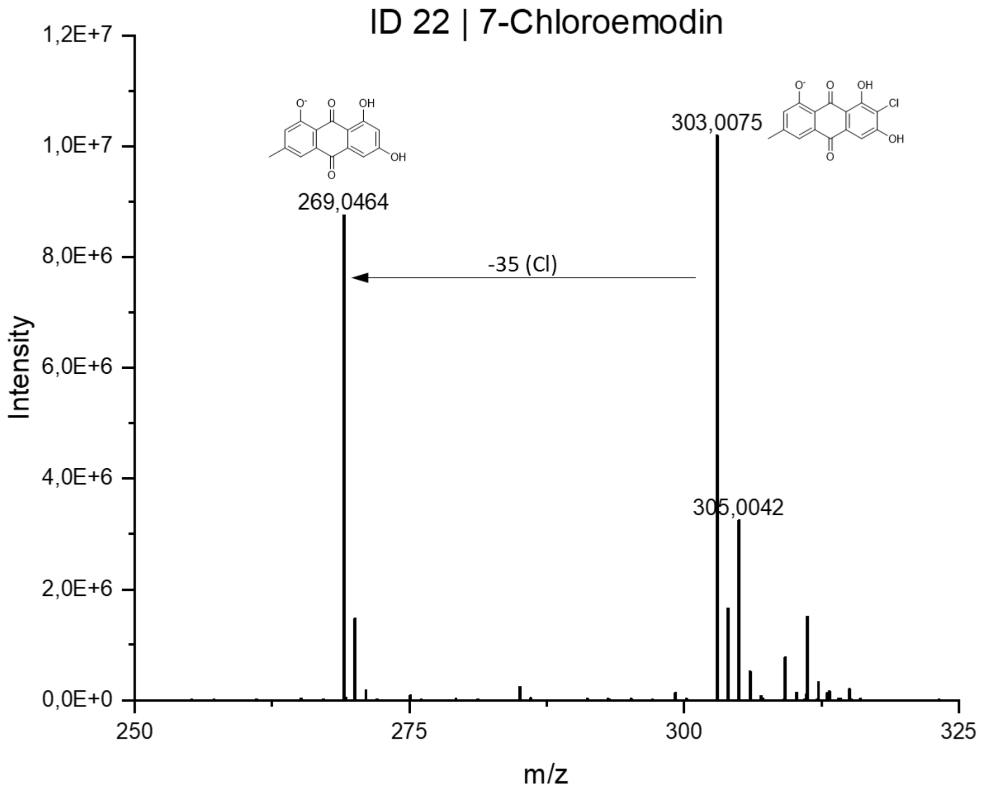

Figure S32. Mass spectrum of ID 22 (7-chloroemodin).

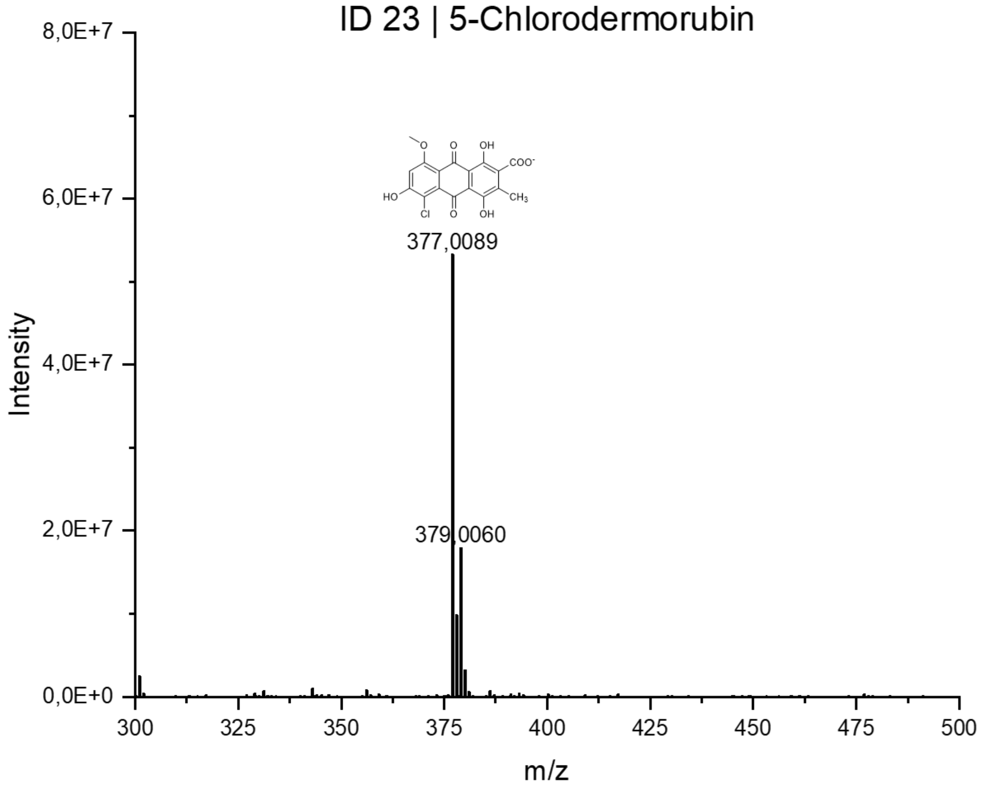

Figure S33. Mass spectrum of ID 23 (5-chlorodermorubin).

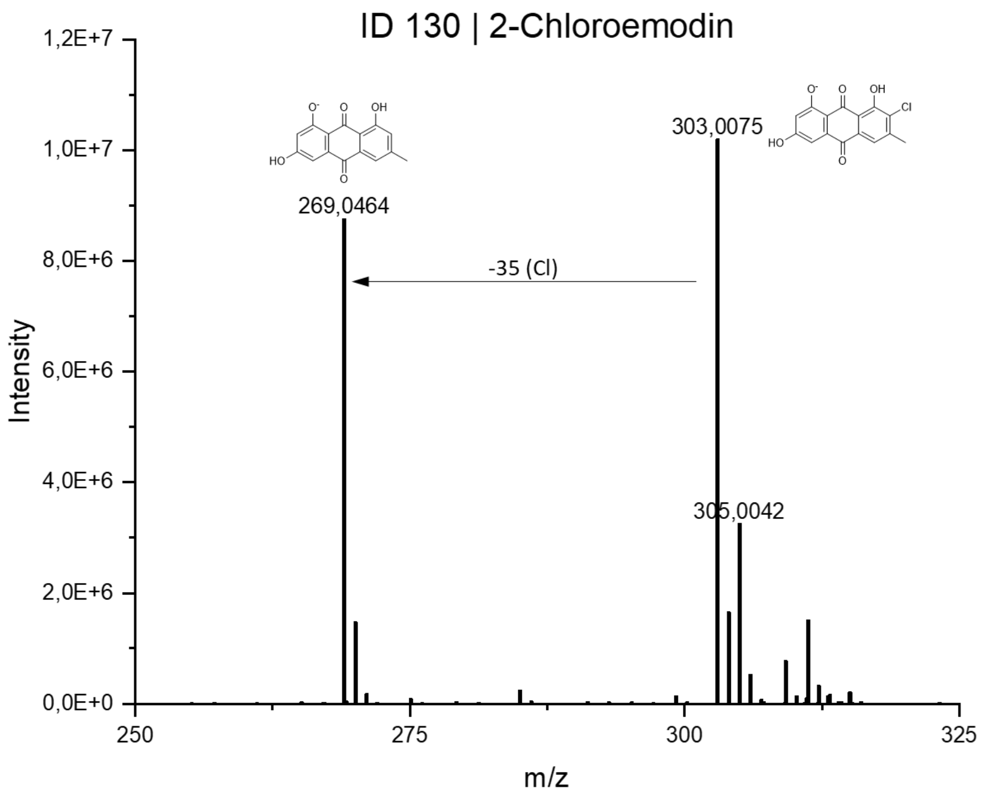

Figure S34. Mass spectrum of ID 130 (2-chloroemodin).

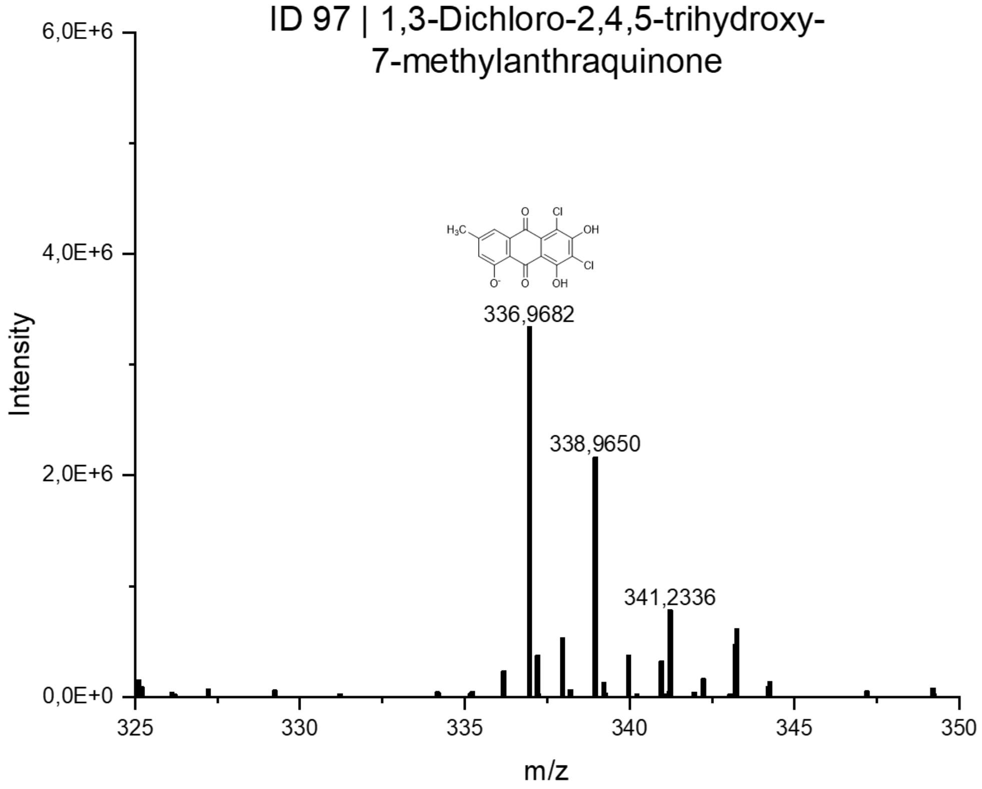

Figure S35. Mass spectrum of ID 97 (1,3-dichloro-2,4,5-trihydroxy-7-methylanthraquinone).

4 Literature

1. Siewert, B., Vrabl, P., Hammerle, F., Bingger, I., & Stuppner, H. (2019). A convenient workflow to spot photosensitizers revealed photo-activity in basidiomycetes. [10.1039/c8ra10181g]. *RSC Adv., 9*(8), 4545-4552, doi:10.1039/c8ra10181g.

2. Hammerle, F., Bingger, I., Pannwitz, A., Magnutzki, A., Gstir, R., Rutz, A., et al. (2021). Targeted Isolation of Photoactive Pigments from Mushrooms Yielded a Highly Potent New Photosensitizer: 7,7'-Biphyscion. *ChemRXiv*, doi:10.26434/chemrxiv.13721770.v1.

3. Chambers, M. C., Maclean, B., Burke, R., Amodei, D., Ruderman, D. L., Neumann, S., et al. (2012). A cross-platform toolkit for mass spectrometry and proteomics. *Nature Biotechnology, 30*(10), 918-920, doi:10.1038/nbt.2377.

4. Pluskal, T., Castillo, S., Villar-Briones, A., & Oresic, M. (2010). MZmine 2: modular framework for processing, visualizing, and analyzing mass spectrometry-based molecular profile data. [10.1186/1471-2105-11-395]. *BMC Bioinf., 11*, No pp. given, doi:10.1186/1471-2105-11-395.

5. Gill, M., & Steglich, W. (1987). Pigments of fungi (Macromycetes). *Fortschr Chem Org Naturst, 51*, 1-317.

6. Gill, M. (1994). Pigments of fungi (Macromycetes). [10.1039/np9941100067]. *Nat. Prod. Rep., 11*(1), 67-90, doi:10.1039/np9941100067.

7. Nothias, L.-F., Petras, D., Schmid, R., Duhrkop, K., Rainer, J., Sarvepalli, A., et al. (2020). Feature-based molecular networking in the GNPS analysis environment. [10.1038/s41592-020-0933-6]. *Nat. Methods, 17*(9), 905-908, doi:10.1038/s41592-020-0933-6.

8. Wang, M., Carver, J. J., Phelan, V. V., Sanchez, L. M., Garg, N., Peng, Y., et al. (2016). Sharing and community curation of mass spectrometry data with Global Natural Products Social Molecular Networking. [10.1038/nbt.3597]. *Nat. Biotechnol., 34*(8), 828-837, doi:10.1038/nbt.3597.

9. Allard, P.-M., Peresse, T., Bisson, J., Gindro, K., Marcourt, L., Pham, V. C., et al. (2016). Integration of Molecular Networking and In-Silico MS/MS Fragmentation for Natural Products Dereplication. [10.1021/acs.analchem.5b04804]. *Anal. Chem. (Washington, DC, U. S.), 88*(6), 3317-3323, doi:10.1021/acs.analchem.5b04804.

10. Rutz, A., Dounoue-Kubo, M., Ollivier, S., Bisson, J., Bagheri, M., Saesong, T., et al. (2019). Taxonomically Informed Scoring Enhances Confidence in Natural Products Annotation. [Original Research]. *Frontiers in Plant Science, 10*(1329), doi:10.3389/fpls.2019.01329.

11. Shannon, P., Markiel, A., Ozier, O., Baliga, N. S., Wang, J. T., Ramage, D., et al. (2003). Cytoscape: A software environment for integrated models of biomolecular interaction networks. [10.1101/gr.1239303]. *Genome Res., 13*(11), 2498-2504, doi:10.1101/gr.1239303.

12. Djoumbou Feunang, Y., Eisner, R., Knox, C., Chepelev, L., Hastings, J., Owen, G., et al. (2016). ClassyFire: automated chemical classification with a comprehensive, computable taxonomy. *Journal of Cheminformatics, 8*(1), 61, doi:10.1186/s13321-016-0174-y.

13. Dührkop, K., Fleischauer, M., Ludwig, M., Aksenov, A. A., Melnik, A. V., Meusel, M., et al. (2019). SIRIUS 4: a rapid tool for turning tandem mass spectra into metabolite structure information. *Nature Methods, 16*(4), 299-302, doi:10.1038/s41592-019-0344-8.

14. Dührkop, K., Shen, H., Meusel, M., Rousu, J., & Böcker, S. (2015). Searching molecular structure databases with tandem mass spectra using CSI:FingerID. *Proceedings of the National Academy of Sciences, 112*(41), 12580-12585, doi:10.1073/pnas.1509788112.

15. Sumner, L. W., Amberg, A., Barrett, D., Beale, M. H., Beger, R., Daykin, C. A., et al. (2007). Proposed minimum reporting standards for chemical analysis. *Metabolomics, 3*(3), 211-221, doi:10.1007/s11306-007-0082-2.
